# The effect of sulforaphane on autism spectrum disorder: systematic review and meta-analysis

**DOI:** 10.17179/excli2025-8239

**Published:** 2025-04-07

**Authors:** Rui Wang, Zhenhui Ren, Yamin Li

**Affiliations:** 1 Xiangya School of Nursing, Central South University, Changsha, Hunan, China; 2 Hunan Provincial People’s Hospital and The First-Affiliated Hospital of Hunan Normal University, Changsha, Hunan, China

**Keywords:** autism spectrum disorder, meta-analysis, randomized controlled trial, sulforaphane

## Abstract

Autism spectrum disorder (ASD) is a complex neurodevelopmental disorder lacking effective treatments. This systematic review and meta-analysis assesses the efficacy and safety of sulforaphane (SFN) for ASD. Eight databases were searched from inception to September 2024, identifying six randomized controlled trials for inclusion. Efficacy outcomes included ASD symptoms measured by the mean difference (MD) or standardized mean difference (SMD), while safety outcomes included adverse events measured by relative risk. Risk of bias was assessed using the Cochrane tool, and evidence certainty was evaluated via the Grade of Recommendations Assessment Development and Evaluation (GRADE). Results showed that SFN significantly improved total symptoms (SMD = -0.27, 95 % confidence interval (CI), -0.42, -0.12), aberrant behavior (SMD = -0.43, 95 % CI, -0.66, -0.19), hyperactivity (SMD = -0.58, 95 % CI, -1.03, -0.13), social interaction (SMD = -0.43, 95 % CI, -0.59, -0.27), social communication (SMD = -0.24, 95 % CI, -0.35, - 0.12), and restricted and repetitive behaviors (RRB) (SMD = -0.16, 95 % CI, -0.31, -0.00). Effects on irritability, anxiety, sensory sensitivity, total social skills, social awareness, social cognition, and social motivation were not statistically significant. Adverse events were similar between intervention and control groups. In conclusion, SFN shows potential in improving ASD symptoms without significant adverse effects. However, results should be interpreted cautiously due to potential influences from assessment tools, outcome assessors, and treatment duration. Further research is needed to confirm the long-term efficacy and safety of SFN for ASD.

## Introduction

Autism spectrum disorder (ASD) is a common neurodevelopmental disorder characterized by difficulties in social interaction and communication alongside restricted interests and repetitive behaviors (Battle, 2013[1]). The prevalence of ASD is increasing significantly. According to the latest data from the Centers for Disease Control and Prevention (CDC), 1 in 36 children aged 8 years are now diagnosed with ASD. Since 2000, the prevalence of ASD has increased by 317 % (Maenner et al., 2023[28]). ASD is typically recognized at age 3, and its symptoms can persist into adulthood, imposing a serious burden on individuals, their families, and society. Compared with those without ASD, individuals with ASD face a markedly higher rate of premature mortality (OR = 2.56; 95 % CI 2.38-2.76) (Hirvikoski et al., 2016[20]), with an average life expectancy of only 36 years (Guan and Li, 2017[18]). Parenting stress affects 70 % of parents of children with ASD, leading to significant psychiatric distress (Shepherd et al., 2021[43]) and financial burdens related to work-related exhaustion, as well as the costs associated with education and training for ASD (Rogge and Janssen, 2019[39]). Lifetime social cost analysis for the United States is projected to reach $ 15 trillion by 2029 (Cakir et al., 2020[6]). Consequently, ASD has emerged as a significant global public health issue. However, the pathogenesis of ASD remains unclear, and effective treatments are still unavailable (McCracken et al., 2021[32]). Therefore, it is essential to explore safe and effective treatments to improve ASD.

Several potential pathogenic mechanisms of ASD provide the possibility of discovering new therapeutic strategies (Liu et al., 2016[25]). First, abnormal synaptic junctions induced by mTOR signaling and improper synaptic pruning are associated with ASD (Ebert and Greenberg, 2013[10]; Paolicelli et al., 2011[37]). Second, neuroinflammation is observed in ASD and is characterized by the overexpression of inflammatory immune-related genes, increased levels of proinflammatory cytokines, and alterations in inflammatory markers (Hughes et al., 2023[22]; Gorji, 2022[15]). Third, oxidative stress, such as a low GSH/GSSG redox ratio and disruption of the balance of ROS/RNS, has been identified in individuals with ASD (Rossignol and Frye, 2014[41]). Fourth, epidemiological studies indicate that approximately 80 % of children with ASD exhibit reduced electron transport chain activity. Fifth, heat shock plays a key role in the development of ASD (Goh et al., 2014[14]). Studies have demonstrated that fever can temporarily alleviate behavioral abnormalities in individuals with ASD. Fever triggers the heat shock response (HSR), leading to the upregulation of heat shock proteins (HSPs). This response enhances suppressed long-range cortical synaptic activity and improves neural signaling among individuals with ASD (Grzadzinski et al., 2018[17]).

Sulforaphane (SFN) is an isothiocyanate derived from cruciferous plants, such as broccoli (Ruhee and Suzuki, 2020[42]). It can cross the blood‒brain barrier and directly affect the central nervous system, offering potential therapeutic benefits against the aforementioned pathological mechanisms. Studies have shown that SFN can reverse iron-induced synaptic alterations (Lavich et al., 2015[23]), inhibit mTOR-dependent neuronal apoptosis (Zhou et al., 2016[49]), and exert anti-neuroinflammatory effects on microglia by inhibiting the JNK/AP-1/NF-κB pathways and activating Nrf2/HO-1 (Subedi et al., 2019[48]). It also induces antioxidant enzymes by modulating the Keap1/Nrf2/ARE pathway (Hu et al., 2011[21]; Magesh et al., 2012[29]) and activates the heat shock response through the upregulation of Hsp27 (Gan et al., 2010[13]). Therefore, SFN represents a promising therapeutic agent for ASD. However, the conclusions regarding the efficacy of SFN against ASD in clinical trials have been inconsistent. Although a network meta-analysis explored the effects of SFN on ASD, only three studies were included, and little is known about the factors that moderate the effects of SFN on ASD (Siafis et al., 2022[44]). Thus, a comprehensive investigation of the effect of SFN on ASD is still needed.

This study aims to explore the efficacy and safety of SFN for treating ASD, and the quality of evidence on the efficacy of SFN against ASD. Also, we aim to perform the subgroup analysis to analyze moderators of the efficacy of SFN against ASD, including the country of study performed, the age of participants, measurement tool, assessor of outcome indicators, and the duration of the SFN intervention, thus to identify the characteristics of effective SFN interventions and provide a reference for clinical practice and future research.

## Methods

### Study protocol and pre-registration

The study followed the Preferred Reporting Items for Systematic Reviews and Meta-Analyses (PRISMA) statement (Haddaway et al., 2022[19]) and the Cochrane Handbook's Preferred Reporting Items for Meta-Analyses (Cumpston et al., 2019[8]). The protocol was registered with PROSPERO (CRD42024489376). Two researchers completed the processes independently and then reviewed the results together. Any disagreements were resolved through discussion with a third party or by mutual agreement.

### Search strategy and selection criteria

A comprehensive search was performed across 6 English databases (PubMed, Embase, CINAHL, ProQuest Dissertations & Theses Global, Web of Science, and Cochrane Library) and 2 Chinese databases (China National Knowledge Infrastructure and Wanfang) from inception to September 2024. The search terms were a combination of Medical Subject Heading terms and keywords, and the constructs were as follows: “autis*” AND Sulforaphane. The complete search strategy is shown in Supplementary Table 1 in the “Supplementary information”. In addition, the same search strategy was used to search for clinicaltrials.gov (https://www.clinicaltrials.gov/) and WHO-ICTRP (https://trialsearch.who.int/). We also screened the references of the included studies to avoid omissions. Studies were included if they met the PICOS criteria as follows: P (Participants): Participants were ASD patients diagnosed with at least one of the following tools: ① Diagnostic and Statistical Manual of Mental Disorders (4^th^ or 5^th^ edition) (Mandy et al., 2012[31]); ② Autism Diagnostic Interview or Autism Diagnostic Interview-Revised (De Bildt et al., 2004[9]); ③ Autism Diagnostic Observation Schedule (ADOS) (Gotham et al., 2007[16]); and ④ Childhood Autism Rating Scale (CARS) (Chlebowski et al., 2010[7]). I (Intervention): SFN. C (Comparison): Control conditions including placebo or a treatment plan that does not include SFN. O (Outcomes): ① Efficacy: The symptoms of ASD should be assessed with validated instruments and reported as continuous variables, including means and standard deviations (SDs) or other formats that can be converted to means and SDs. ② Safety: The incidence of adverse events (including insomnia, irritability, and headache). S (Study design): Randomized controlled trials (RCTs). Studies were excluded if they (1) were non-Chinese or non-English articles; (2) did not explore the independent role of SFN in improving the efficacy of ASD; or (3) were duplicate publications.

### Study selection and data extraction

All retrieved articles were imported into Endnote (version 20.0) to remove duplicates. Titles and abstracts were reviewed for initial screening, followed by a full-text review to identify the final included literature.

The following characteristics of the included studies were extracted via a predesigned worksheet: first author, year of publication, demographics of the participants (country, age, percentage of males, ethnicity, diagnostic criteria for autism, mental retardation, and gastrointestinal symptomatology), details of the intervention group (source, form, dose, frequency, and duration of SFN), details of the control group (form, dose, frequency and duration of placebo), assessment tools and assessors of the outcomes.

We only included data measured immediately after the intervention. Missing study information and data were obtained by contacting the corresponding author.

### Risk of bias and GRADE assessment

The Cochrane Risk of Bias tool 2 (ROB 2) (Sterne et al., 2019[47]), which contains five domains, including the randomization process, deviations from intended interventions, missing outcome data, measurement of the outcome, selection of the reported result, and, finally, overall bias, was used to assess the quality of the included studies. There are multiple different signaling questions under each domain, and each signaling question generally has 5 choices: Yes (Y), Probably Yes (PY), Probably No (PN), No (N), and No Information (NI). The risk of bias for each domain was categorized into three levels: “low risk of bias”, “some concerns”, and “high risk of bias” (Liu et al., 2021[26]).

The Grade of Recommendations Assessment Development and Evaluation (GRADE) system was used to evaluate the quality of evidence (Brozek et al., 2009[4][5], 2011[3]). The quality of evidence can be categorized as “high”, “medium”, “low” or “very low”. Given that all of the individual studies we analyzed were randomized controlled trials, the initial assessment was designated “high” and then downgraded to “moderate” (step 1) on the basis of various factors that reduce the quality of the evidence (e.g., high risk of bias), “low” (step 2) or “very low” (step 3). However, reductions in quality may be offset by other factors that may increase quality, such as large effect sizes and dose-effect response gradients.

### Statistical analysis

Review Manager 5.4 and Stata 14.0 software were used for data analysis. Changes in the means and SDs of ASD-related symptoms (referred to as changes in scores in subsequent articles) were calculated with the formulas recommended in the Cochran manual. For continuous variables, the standardized mean difference (SMD) and its corresponding 95 % confidence interval (CI) were chosen if the same outcome was measured with different assessment tools; otherwise, the weighted mean difference (WMD) and its 95 % CI were used (Nasser, 2020[35]). The effect size was categorized into 3 levels: an effect size < 0.2 was considered a small effect, an effect size = 0.2--0.5 was considered moderate, and an effect size > 0.5 was considered large. For dichotomous variables, the relative risk (RR) and 95 % CI were used as effect indicators. Heterogeneity was tested via the Cochran Q chi-square test and *I*^2^ statistic. The fixed-effects model (*P* ≥ 0.1 and *I*^2^ < 50 %) or a random-effects model (*P* < 0.1 or *I*^2 ^≥ 50 %) was chosen based on the *P* and *I*^2^ values (Nasser, 2020[35]). Subgroup analyses were performed to analyze the moderating effects of drug efficacy. The combined estimates of the different subgroups were compared by examining whether the 95 % CIs of the subgroups overlapped. If the 95 % CIs overlapped, the differences between subgroups were considered statistically insignificant; otherwise, they were considered statistically significant (Nasser, 2020[35]).

### Sensitivity analysis

Sensitivity analysis was used to assess the robustness of the meta-analysis results. If effect sizes remained relatively stable after excluding a particular study, the results were considered robust. Conversely, if the results change significantly or if diametrically opposite conclusions emerge, the findings may be less stable and should be interpreted with caution.

### Publication bias

Publication bias occurred when statistically significant results were more likely to be published, leading to an unrepresentative view of the overall research in the field. To assess whether publication bias affected the reliability of the results, if there were more than ten studies, we conducted publication bias assessment via a funnel plot and Egger's test (Sterne et al., 2011[46]). If the funnel plot displayed a symmetrical inverted funnel shape or if Egger's regression results were not significant, publication bias had a minimal effect on the meta-analysis results (Egger et al., 1997[11]; Light and Pillemer, 1984[24]; Rosenthal, 1979[40]).

## Results

### Study selection

**Figure 1:**
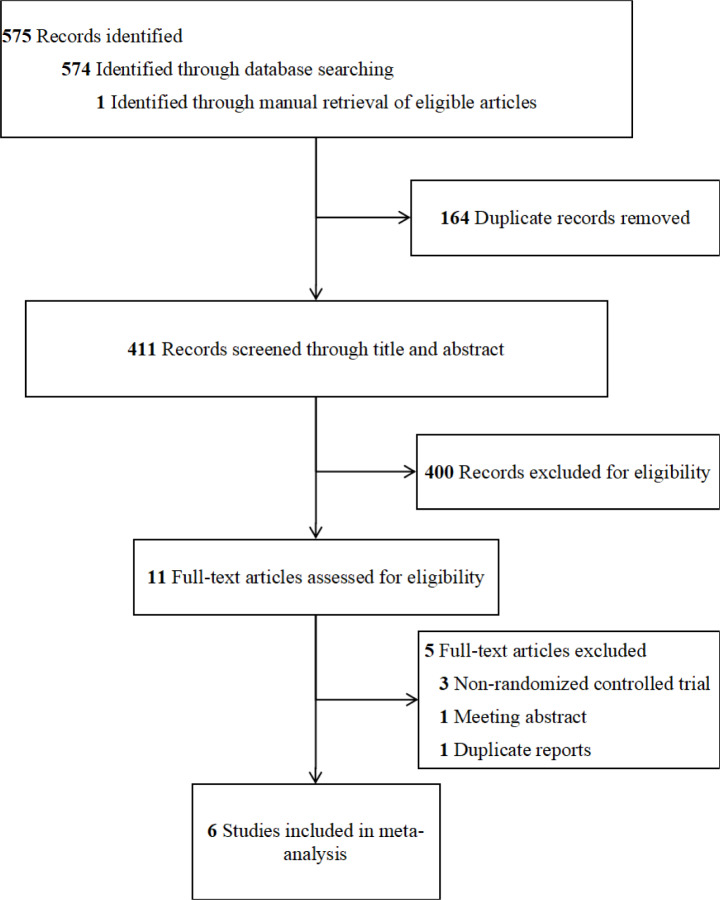
Flowchart for study selection

A total of 574 articles were retrieved from 8 databases, and the other one (Study Registration Date: 2016-09-21; Clinical trial number: NCT02909959) was from clinicaltrials.gov (https://www.clinicaltrials.gov/) (Politte, 2020[38]). After 164 duplicates were removed and 411 titles and abstracts were screened, 11 full texts were assessed for eligibility. Finally, 6 studies were included in the meta-analysis. The process of literature screening and the results are shown in Figure 1(Fig. 1).

### Study characteristics

The specific characteristics of the included studies were listed in Table 1(Tab. 1). The six included studies were published from 2014-2023; three studies were from the United States (Singh et al., 2014[45]; Zimmerman et al., 2021[50]; Politte, 2020[38]), one was from the Czech Republic (Magner et al., 2023[30]), one was from Iran (Momtazmanesh et al., 2020[34]), and one was from China (Ou et al., 2024[36]). The majority of participants were male (63.3 %-100 %) and aged 3 to 30 years, with four studies involving minors (Momtazmanesh et al., 2020[34]; Magner et al., 2023[30]; Zimmerman et al., 2021[50]; Ou et al., 2024[36]) and two involving adults (Singh et al., 2014[45]; Politte, 2020[38]). The frequency of intervention for each study was once daily. The intervention duration varied across studies (from 10 to 36 weeks). Except for one study with an intervention dose of 50 μmol (Magner et al., 2023[30]), the other studies were according to the participants' body weight. The intervention dose, period, and frequency of the control group were the same as those of the corresponding intervention groups. Zimmerman et al. (2021[50]) changed the assessment of the OACIS-I scale in the following way: for the OACIS-I, the general score and subscale values were recoded as follows: a score of 4 (indicating no change) was changed to 0; scores from 3 to 1 were changed to +1 to +3 to indicate improvement; and scores from 5 to 7 were changed to -1 to -3 to indicate worsening.

### Risk of bias and GRADE assessment

**Figure 2:**
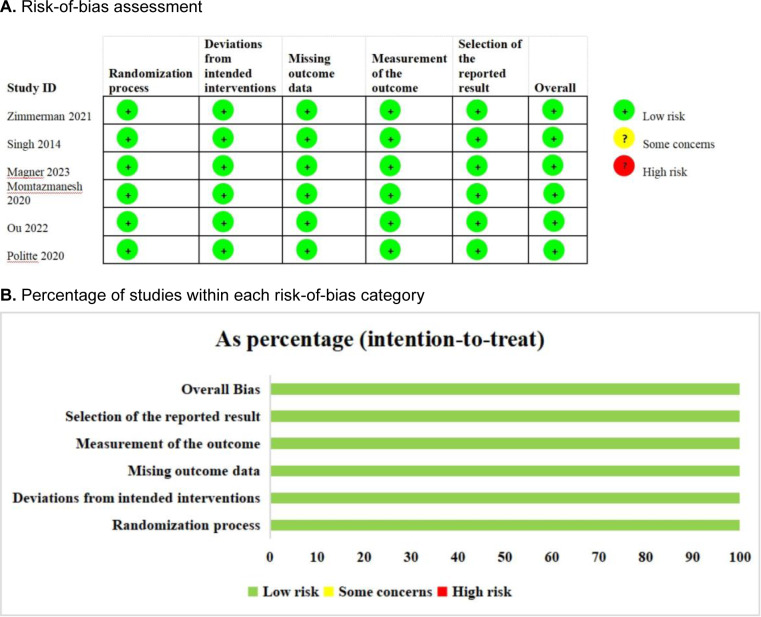
Risk-of-bias summary of the included studies

All six studies (Momtazmanesh et al., 2020[34]; Singh et al., 2014[45]; Magner et al., 2023[30]; Zimmerman et al., 2021[50]; Ou et al., 2024[36]; Politte, 2020[38]) (100 %) were categorized as low risk (Figure 2(Fig. 2)). The details of the assessment of the risk of bias are shown in Supplementary Table 2. We used the GRADE method to assess the quality of the study outcomes (Table 2(Tab. 2)), in which the quality of 3 outcome indicators was assessed as high, 10 as moderate, 2 as low, and 1 as very low. The detailed results are presented in Supplementary Table 3.

### Publication bias

Owing to the small number of studies (< 10) that were ultimately included, no test for publication bias was performed.

## Meta-Analysis Results

### Efficacy

Table 2(Tab. 2) illustrated the meta-analysis summary for all the outcomes. SFN had a significant positive effect on total symptoms (5 studies (Politte, 2020[38]; Singh et al., 2014[45]; Zimmerman et al., 2021[50]; Magner et al., 2023[30]; Ou et al., 2024[36]), containing 27 data and 1508 participants; SMD = -0.27, 95 % CI, -0.42, -0.12; *P *= 0.002, *I*^2 ^= 49 %), aberrant behavior (2 studies (Singh et al., 2014[45]; Zimmerman et al., 2021[50]), containing 8 data and 308 participants; SMD = -0.43, 95 % CI, -0.66, -0.19;* P *= 0.28, *I*^2 ^= 19 %), hyperactivity (4 studies (Politte, 2020[38]; Singh et al., 2014[45]; Zimmerman et al., 2021[50]; Momtazmanesh et al., 2020[34]) with 14 data and 586 participants; SMD = -0.58, 95 % CI, -1.03, -0.13; *P *< 0.001, *I*^2 ^= 85 %), social interaction (4 studies (Singh et al., 2014[45]; Zimmerman et al., 2021[50]; Magner et al., 2023[30]; Ou et al., 2024[36]), containing 13 data and 646 participants; SMD = -0.43, 95 % CI, -0.59, -0.27; *P *= 0.31, *I*^2 ^= 13 %), social communication (5 studies (Politte, 2020[38]; Singh et al., 2014[45]; Zimmerman et al., 2021[50]; Magner et al., 2023[30]; Ou et al., 2024[36]), containing 28 data and 1206 participants; SMD = -0.24, 95 % CI, -0.35, - 0.12; *P *= 0.15, *I*^2 ^= 22 %), restricted and repetitive behaviors (RRB) (6 studies (Momtazmanesh et al., 2020[34]; Singh et al., 2014[45]; Magner et al., 2023[30]; Zimmerman et al., 2021[50]; Ou et al., 2024[36]; Politte, 2020[38]), containing 37 data and 1801 participants; SMD = -0.16, 95 % CI, -0.31, -0.00; *P *< 0.001, *I*^2 ^= 60 %). The effect sizes of hyperactivity were large; those of total symptoms, aberrant behavior, social interaction and social communication were medium; and those of RRB were small.

SFN had nonsignificant effect on the irritability (3 studies (Politte, 2020[38]; Zimmerman et al., 2021[50]; Momtazmanesh et al., 2020[34]), containing 7 data and 318 participants; SMD = -1.17, 95 % CI, -2.65, 0.31; *P* < 0.001, *I*^2 ^= 95 %), anxiety (2 studies (Singh et al., 2014[45]; Zimmerman et al., 2021[50]), containing 7 data and 268 participants; SMD = -0.35, 95 % CI, -0.77, 0.07; *P *= 0.01, *I*^2 ^= 63 %); sensory sensitivity (2 studies (Singh et al., 2014[45]; Zimmerman et al., 2021[50]), containing 8 data and 308 participants; SMD = -0.12, 95 % CI, -0.35, 0.12; *P *= 0.93, *I*^2 ^= 0 %), total social skills (5 studies (Singh et al., 2014[45]; Magner et al., 2023[30]; Zimmerman et al., 2021[50]; Ou et al., 2024[36]; Politte, 2020[38]), containing 16 data and 914 participants; SMD = -0.03, 95 % CI, -0.16, 0.10; *P *= 0.43, *I*^2 ^= 2 %), social awareness (2 studies (Politte, 2020[38]; Zimmerman et al., 2021[50]), containing 5 data and 198 participants; MD = -0.46, 95 % CI, -1.62, 0.70; *P *= 0.92, *I*^2 ^= 0 %), social cognition (2 studies (Politte, 2020[38]; Zimmerman et al., 2021[50]), containing 5 data and 198 participants; MD = 0.80, 95 % CI, -1.31, 2.91; *P *= 0.44, *I*^2 ^= 0 %), social motivation (3 studies (Politte, 2020[38]; Zimmerman et al., 2021[50]; Momtazmanesh et al., 2020[34]), containing 12 data and 516 participants; SMD = -0.02, 95 % CI, -0.31, 0.27; *P *= 0.002, *I*^2 ^= 63 %). The forest plots for each study metric are shown in Supplementary Figure 1.

### Adverse effects

Three studies reported the adverse effects of SFN interventions on ASD, including insomnia (3 studies (Politte, 2020[38]; Singh et al., 2014[45]; Zimmerman et al., 2021[50]), containing 3 data and 143 participants; RR = 0.80, 95 % CI, 0.27, 2.33, *P *= 0.68; *P *= 0.17, *I*^2 ^= 43 %), irritability (3 studies (Politte, 2020[38]; Singh et al., 2014[45]; Zimmerman et al., 2021[50]), containing 3 data and 143 participants; RR = 1.46, 95 % CI, 0.49, 4.34, *P *= 0.50; *P *= 0.43, *I*^2 ^= 0 %, and headache (3 studies (Politte, 2020[38]; Singh et al., 2014[45]; Momtazmanesh et al., 2020[34]), containing 3 data and 148 participants; RR = 1.34, 95 % CI, 0.45, 3.95, *P *= 0.60; *P *= 0.96, *I*^2 ^= 0 %). There were no statistically significant differences in the incidence of the three adverse events between the intervention group and the control group (Details shown in Supplementary Figure 2).

### Sensitivity and subgroup analysis

The leave-one-out sensitivity analyses did not significantly change the results, suggesting the robustness of the outcomes (Supplementary Figure 3).

Subgroup analyses were conducted according to the age of the participants, measurement tool, assessor of outcome indicators, and duration of intervention in the efficiency of SNF against ASD.

Among them, the measurement tool, the assessor of the outcome indicator, and the duration of intervention significantly affected the efficiency of SFN against ASD, with the following results: (1) Measurement tool. SFN significantly improved social communication in the OACIS-I subgroup (MD, -0.32; 95 % CI, -0.44, -0.20) but not in the SRS subgroup (MD, 2.03; 95 % CI, -0.67, 4.73). SFN significantly improved total symptoms in the OACIS-I subgroup (MD, -0.18; 95 % CI. -0.35, -0.01) but not in the ABC (MD, -8.20; 95 % CI, -17.32, 0.93) and OACIS-S (MD, -0.06; 95 % CI, -0.23, 0.10) subgroups; SFN significantly improved RRB in the ABC subgroup (MD, -0.53; 95 % CI, -0.93, - 0.14) but not in the RBS-R (MD, 2.80; 95 % CI, -0.19, 5.78), SRS (MD, -0.34; 95 % CI, -3.05, 2.38), or OACIS-I (MD, -0.10; 95 % CI, -0.22, 0.03) subgroups. (2) Assessors of outcome indicators. The results indicated that SFN significantly improved social communication in the professional subgroup (SMD, -0.33; 95 % CI, -0.46, -0.20) but not in the caregiver subgroup (SMD, 0.10; 95 % CI, -0.15, 0.34). (3) Duration of the intervention. The results indicated that SFN significantly improved aberrant behavior in the ≥ 10-week subgroup (SMD, -0.50; 95 % CI, -0.80, -0.20) but not in the < 10-week subgroup (SMD, -0.31; 95 % CI, -0.70, 0.07), and SFN significantly improved hyperactivity in the ≥ 10-week subgroup (SMD, -0.73; 95 % CI, -1.42, -0.03) but not in the < 10-week subgroup (SMD, -0.41; 95 % CI, -1.00, 0.19).

The remaining subgroup analyses did not reveal statistically significant differences across subgroups. The results of the subgroup analyses are shown in Supplementary Figure 4.

## Discussion

To the best of our knowledge, this study is the first meta-analysis to systematically explore the efficiency and safety of SFN for treating ASD. The results demonstrated that SFN safely improves the core symptoms of ASD, including total symptoms, RRB, social interactions, social communication, hyperactivity, and aberrant behavior, but it did not increase the irritability, anxiety, sensory sensitivity, total social skills, social awareness, social cognition, or social motivation of ASD patients. Moreover, the conclusions were well founded because of the high-quality evidence. These findings provide a new choice for healthcare personnel to treat ASD. 

SFN has the potential to treat ASD mainly because it can improve abnormal synaptic function (Lavich et al., 2015[23]), inhibit neuronal apoptosis (Zhou et al., 2016[49]), reduce neuroinflammation (Subedi et al., 2019[48]) and oxidative stress (Hu et al., 2011[21]; Magesh et al., 2012[29]), and activate the heat shock response (Gan et al., 2010[13]); however, these potential mechanisms need to be revealed by further RCTs. Among the six RCTs included in our study, only Zimmerman et al. (2021[50]) with their small sample size and cross-sectional design, explored biomarkers (including glutathione redox status, mitochondrial respiration, inflammatory markers and heat shock), which limits our understanding of the mechanisms by which SFN affects ASD. Future research should employ robust and scientific experimental designs and analytical strategies to identify biodiagnostic markers with high specificity, sensitivity, and reliability of SFN against ASD. Additionally, none of the included studies measured the participants' gut flora. Importantly, the composition of gut microorganisms can influence the bioavailability of broccoli sprouts, which in turn affects the efficacy of SFN against ASD (Bouranis et al., 2021[2]). Therefore, future research should consider the impact of participants' gut flora profiles on the efficacy of SFN against ASD.

Notably, we found that the SFN has different effects on different types of social behaviors. The SFN can improve social communication and social interaction, which are the final steps in social behavior involving choosing actions or responses that maximize rewards or minimize disadvantages, while it does not improve social awareness, social cognition, or social motivation-the early stage of social behavior that involves recognizing, perceiving, and valuing social information. Further research is needed to explore the reasons for this difference. Meanwhile, when evaluating the efficacy of SFN against ASD, it is important not to treat all ASD symptoms as a monolithic group.

Additionally, the incidence of adverse effects of SFN against ASD did not significantly differ between the intervention and control groups, suggesting that SFN is safe. These findings are consistent with the previous systematic review, which indicates that SFN is a safe and effective therapeutic option for ASD (McGuinness and Kim, 2020[33]). Currently, aripiprazole and risperidone are the two FDA-approved medications for ASD; however, they may cause serious side effects, such as obesity, diabetes, and movement disorders (Lynch et al., 2017[27]; Fung et al., 2016[12]). Therefore, the findings of our study provide a new option for treating ASD.

Subgroup analyses revealed that the assessment tools and assessors of the outcome indicators significantly influence the efficacy of SFN against ASD. These findings suggest that the method used to assess ASD is the main factor influencing the efficacy of SFN against ASD. Given the lack of biomarkers for ASD, the evaluation of ASD mainly depends on the scales being subjectively rated. The differences between the results of different scales highlight the limitations of subjective assessments and underscore the need to identify biomarkers for ASD. Additionally, the duration of the intervention is another factor affecting the efficacy of SFN against ASD. These results indicated that SFN improves aberrant behavior and hyperactivity of ASD only when the duration of SFN is 10 weeks or more. Notably, this meta-analysis only examined the immediate efficacy of SFN against ASD and did not assess the long-term impact of SFN on ASD. Currently, it is unclear whether and how the duration of SFN intervention affects its efficacy. Future studies should compare the impact of different length of SFN against ASD to identify the minimum duration required for achieving long-term benefits.

### Limitations

There are several limitations of this study. First, only the Chinese and English literature was searched in this study, which may introduce selection bias and limit the comprehensiveness of the search results. Second, the outcome indicators were measured via various scales, and despite the use of the recommended SMD, some indicators exhibited high heterogeneity, which reduced the interpretability and applicability of the results. Finally, only post-intervention data were analyzed because of inconsistent and inadequate reporting of follow-up data in the included studies.

## Conclusion

This meta-analysis finds high-quality evidence that SFN can safely and effectively improve the core symptoms of ASD. Only performing SFN no less than 10 weeks can affect aberrant behavior and hyperactivity of ASD. Additionally, the efficacy of SFN against ASD may be influenced by the assessment tool and the assessor of the outcome indicator, which highlights the necessity of exploring biomarkers of ASD. Our findings provide guidance for the healthcare personnel to implement SFN for improving ASD. High-quality RCTs with follow-up studies are recommended to provide more robust evidence for these findings.

## Notes

Rui Wang and Zhenhui Ren contributed equally to this work.

## Declaration

### Acknowledgments and funding information

This study was supported by the Natural Science Foundation of Hunan Province of China (grant number 2023JJ30764) and the National Natural Science Foundation of China (grant number 82371553).

### Declaration of competing interests

The authors declare that they have no competing interests.

### Authors' contributions

Rui Wang: Conceptualization, methodology, formal analysis, investigation, writing - original draft, visualization; Zhenhui Ren: Conceptualization, methodology, formal analysis, investigation, writing - original draft, visualization; Yamin Li: Methodology, resources, writing - review & editing, project administration, funding acquisition. All the authors approved the final submitted version.

### Using Artificial Intelligence (AI)

No artificial intelligence (AI)-assisted technologies (e.g., Large Language Models [LLMs], chatbots, or image creations) were used in the submission process to complete the manuscript.

## Supplementary Material

Supplementary information

## Figures and Tables

**Table 1:**
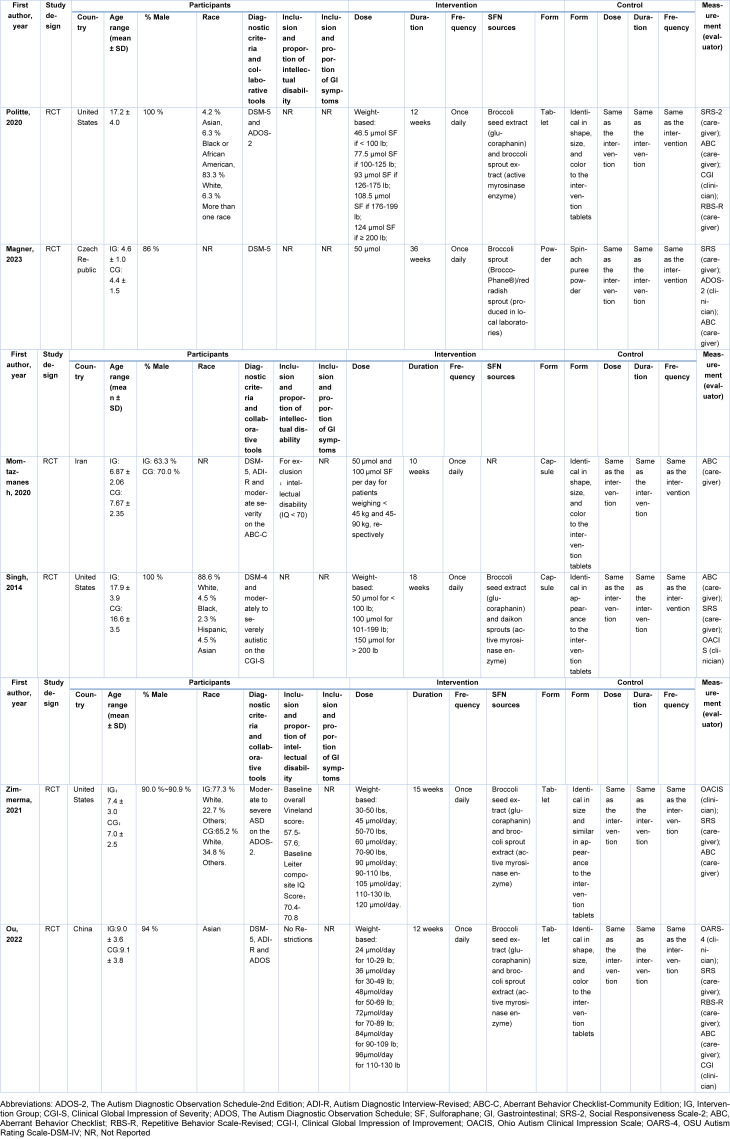
Characteristics of the included studies

**Table 2:**
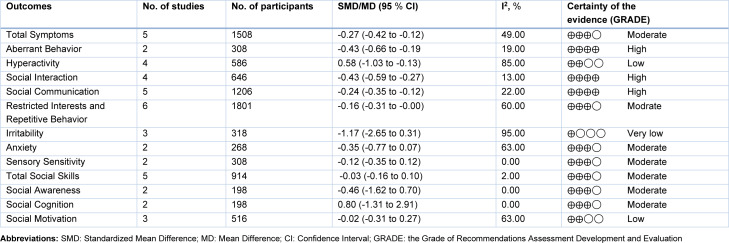
Meta-analysis summary of the included outcomes
