# Supplementary material for: The effect of sulforaphane on autism spectrum disorder: systematic review and meta-analysis
Source: EXCLI J. 2025 Apr 7;24:542–57. doi: 10.17179/excli2025-8239 (PMC12127520; doi:10.17179/excli2025-8239)
Supplement: Supplementary information [file EXCLI-24-542-s-001.pdf]

EXCLI Journal 2025;24:542-557 – ISSN 1611-2156
Received: January 31, 2025, accepted: April 02, 2025, published: April 07, 2025

Supplementary information to:

Review article:

THE EFFECT OF SULFORAPHANE ON AUTISM SPECTRUM DISORDER: SYSTEMATIC REVIEW AND META-ANALYSIS

Rui Wang[2#], Zhenhui Ren[2#], Yamin Li[1, 2*]

[1] Hunan Provincial People's Hospital and The First-Affiliated Hospital of Hunan Normal University, Changsha, Hunan, China
[2] Xiangya School of Nursing, Central South University, Changsha, Hunan, China

[#] These authors contributed equally to this work.

* **Corresponding author:** Yamin Li, PhD, RN, Professor, Hunan Provincial People's Hospital and The first-affiliated hospital of Hunan Normal University, Changsha, Hunan 410005, China. 61 Jiefang West Road of Furong District, Changsha 410011, China. Phone: +86 159-7313-3336; E-mail: amin5433@163.com

https://dx.doi.org/10.17179/excli2025-8239

This is an Open Access article distributed under the terms of the Creative Commons Attribution License (https://creativecommons.org/licenses/by/4.0/).

EXCLI Journal 2025;24:542-557 – ISSN 1611-2156
Received: January 31, 2025, accepted: April 02, 2025, published: April 07, 2025

Content:

Supplementary Figure 1: **Forest plot meta-analyses for different outcomes** S2

Supplementary Figure 2: **Forest plot for adverse effects** S8

Supplementary Figure 3: **Leave-one-out sensitivity analyses** S9

Supplementary Figure 4: **Subgroup analyses of each outcome according to national income levels, ages, measurement tools, assessors of outcome indicators and intervention duration** S16

Supplementary Figure 1: Forest plot meta-analyses for different outcomes

Study or Subgroup	Experimental Mean	SD	Total	Control Mean	SD	Total	Weight	Std. Mean Difference IV, Random, 95% CI
Magner 2023 week 18 (ABC)	-4.9	16.27	15	-8.1	22.35	13	2.7%	0.16 [-0.58, 0.90]
Magner 2023 week 36 (ABC)	1.6	12.39	15	-1	14.06	13	2.7%	0.19 [-0.55, 0.94]
Ou 2022 week 12 (OACIS-I)	2.89	0.63	49	3.51	0.58	42	4.8%	-1.01 [-1.45, -0.57]
Ou 2022 week 12 (OACIS-S)	-0.17	0.71	51	0.01	0.79	43	5.0%	-0.24 [-0.65, 0.17]
Ou 2022 week 12 (OARS-4)	-0.33	0.36	51	-0.09	0.39	43	5.0%	-0.64 [-1.05, -0.22]
Ou 2022 week 4 (OACIS-I)	3.61	0.56	49	3.79	0.52	42	5.0%	-0.33 [-0.74, 0.09]
Ou 2022 week 4 (OACIS-S)	0.13	0.71	51	0.17	0.72	43	5.0%	-0.06 [-0.46, 0.35]
Ou 2022 week 4 (OARS-4)	-0.12	0.36	51	-0.01	0.33	43	5.0%	-0.31 [-0.72, 0.09]
Ou 2022 week 8 (OACIS-I)	3.26	0.56	49	3.71	0.52	42	4.8%	-0.82 [-1.25, -0.39]
Ou 2022 week 8 (OACIS-S)	0.1	0.71	51	0.09	0.72	43	5.0%	0.01 [-0.39, 0.42]
Ou 2022 week 8 (OARS-4)	-0.23	0.36	51	-0.03	0.33	43	5.0%	-0.57 [-0.99, -0.16]
Politte 2020 week 12 (OACIS-S)	-0.02	2.94	24	0	1.47	24	3.7%	-0.01 [-0.57, 0.56]
Politte 2020 week 4 (OACIS-S)	-0.04	2.94	24	0.04	1.47	24	3.7%	-0.03 [-0.60, 0.53]
Politte 2020 week 8 (OACIS-S)	-0.03	2.94	24	0.02	1.47	24	3.7%	-0.02 [-0.59, 0.54]
Singh 2014 week 10 (ABC)	-19.61	27.91	22	3.31	12.62	13	2.8%	-0.95 [-1.68, -0.23]
Singh 2014 week 10 (OACIS-I)	3.88	0.33	26	3.86	0.36	14	3.2%	0.06 [-0.59, 0.71]
Singh 2014 week 10 (OACIS-S)	-0.07	3.42	26	-0.1	2.99	14	3.2%	0.01 [-0.64, 0.66]
Singh 2014 week 18 (ABC)	-21.44	21.7	25	-2	14.52	10	2.6%	-0.95 [-1.72, -0.18]
Singh 2014 week 18 (OACIS-I)	3.81	0.4	26	3.93	0.27	14	3.2%	-0.33 [-0.98, 0.33]
Singh 2014 week 18 (OACIS-S)	0.04	3.42	26	-0.03	2.81	14	3.2%	0.02 [-0.63, 0.67]
Singh 2014 week 4 (ABC)	-12.69	21.26	26	0.77	6.63	13	3.0%	-0.74 [-1.43, -0.05]
Singh 2014 week 4 (OACIS-I)	4.04	0.2	26	4.14	0.53	14	3.2%	-0.28 [-0.93, 0.37]
Singh 2014 week 4 (OACIS-S)	0	3.62	26	-0.17	2.96	14	3.2%	0.05 [-0.60, 0.70]
Zimmerman 2021 week 15 (ABC)	0.29	0.59	17	0.24	0.56	17	3.1%	0.08 [-0.59, 0.76]
Zimmerman 2021 week 15 (OACIS-I)	-19.5	18.47	12	-2.92	16.1	12	2.3%	-0.92 [-1.77, -0.07]
Zimmerman 2021 week 7 (ABC)	-2	18.68	15	-11	16.99	15	2.8%	0.49 [-0.24, 1.22]
Zimmerman 2021 week 7 (OACIS-I)	0.29	0.69	17	0.18	0.39	17	3.1%	0.19 [-0.48, 0.87]
**Total (95% CI)**			**845**			**663**	**100.0%**	**-0.27 [-0.42, -0.12]**

Heterogeneity: Tau² = 0.07; Chi² = 51.11, df = 26 (P = 0.002); I² = 49%
Test for overall effect: Z = 3.56 (P = 0.0004)

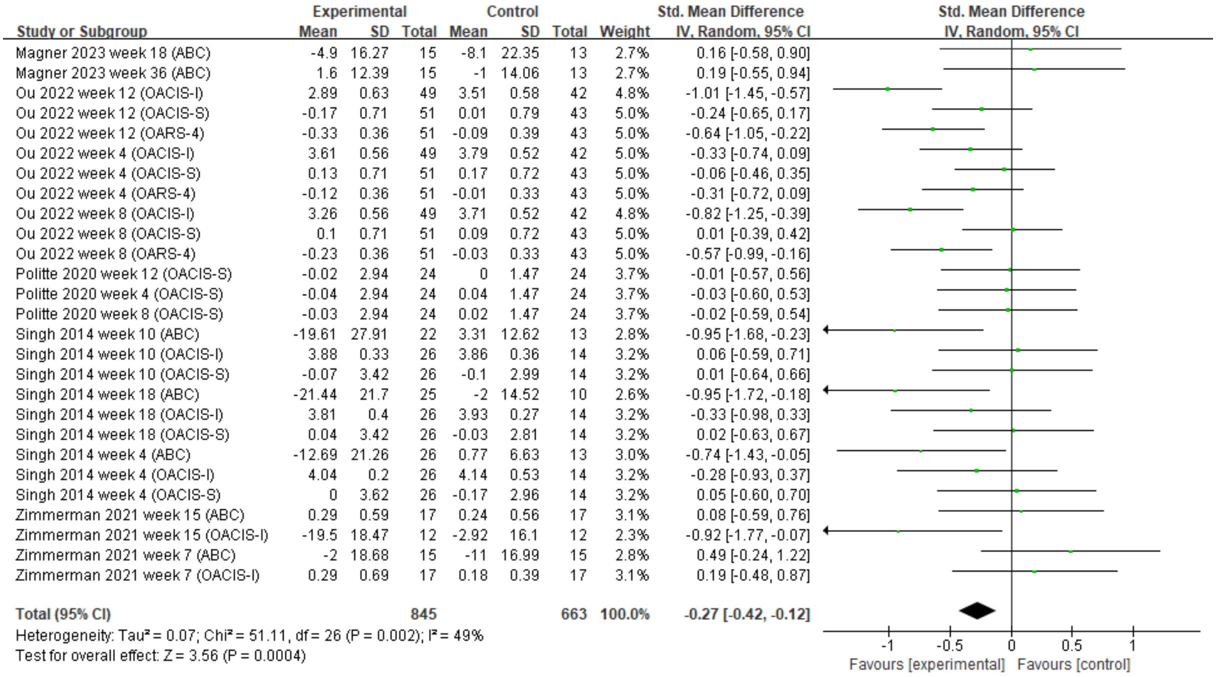

Supplementary Figure 1.1: Forest plot of total symptoms with autism spectrum disorder

EXCLI Journal 2025;24:542-557 – ISSN 1611-2156
Received: January 31, 2025, accepted: April 02, 2025, published: April 07, 2025

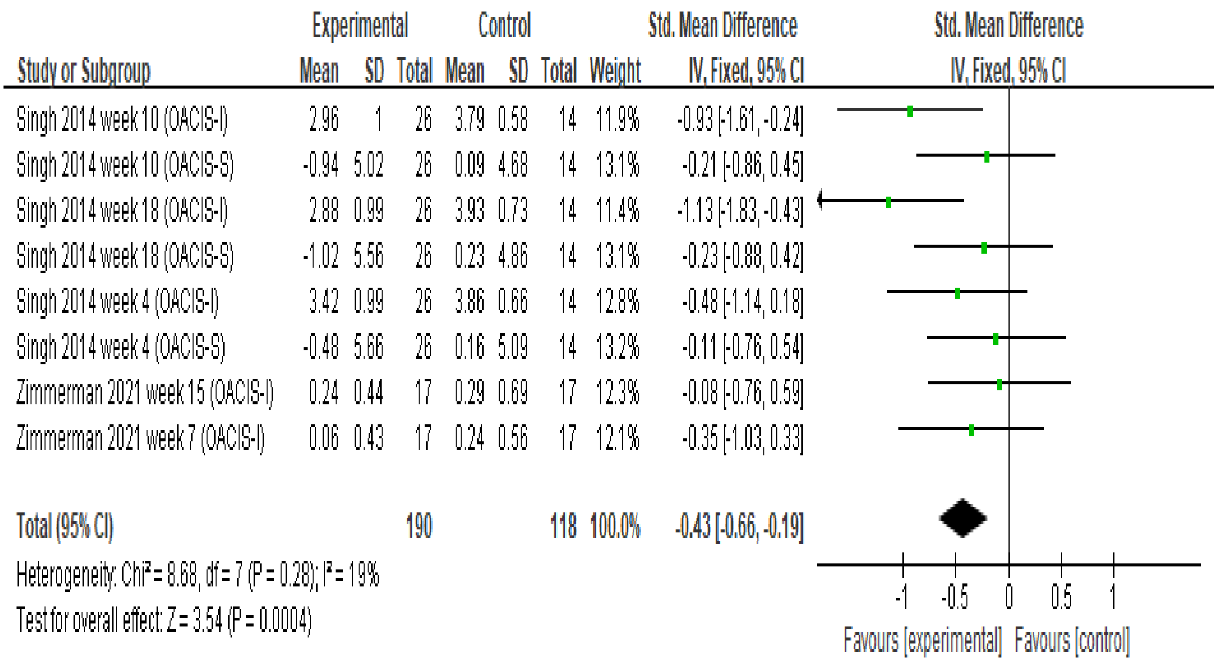

Supplementary Figure 1.2: Forest plot of aberrant behavior with autism spectrum disorder

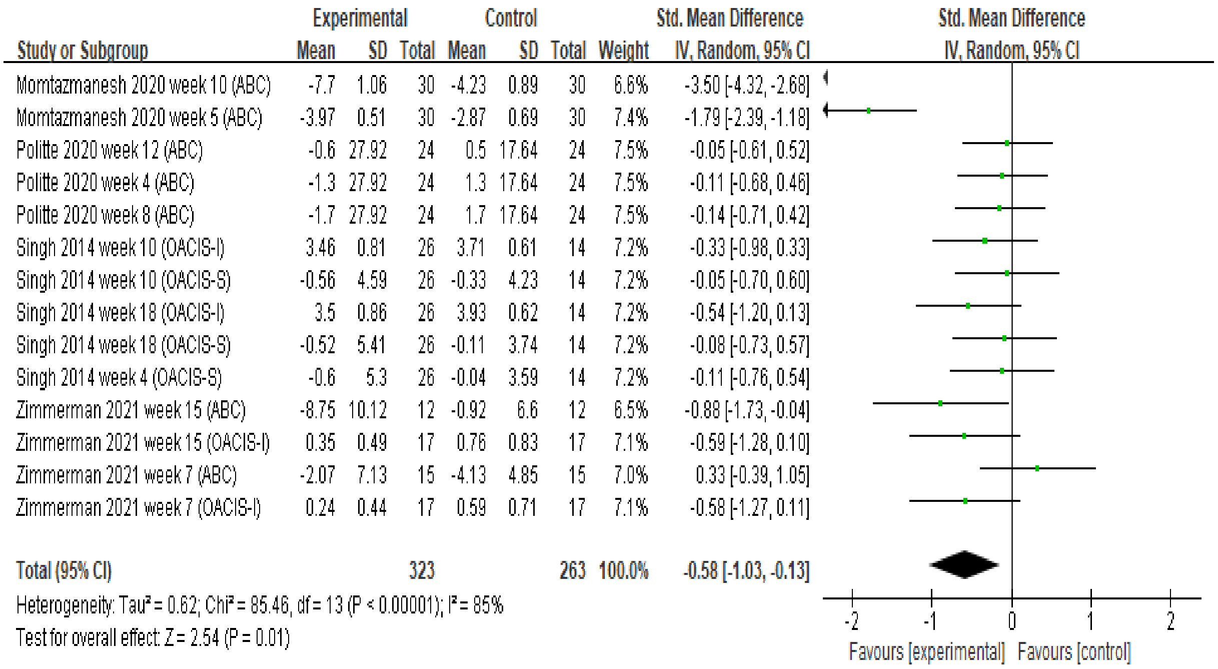

Supplementary Figure 1.3: Forest plot of hyperactivity with autism spectrum disorder

EXCLI Journal 2025;24:542-557 – ISSN 1611-2156
Received: January 31, 2025, accepted: April 02, 2025, published: April 07, 2025

Study or Subgroup	Experimental Mean	Experimental SD	Experimental Total	Control Mean	Control SD	Control Total	Weight	Std. Mean Difference IV, Fixed, 95% CI
Magner 2023 week 18 (ADOS)	-2.4	6.2	15	-1.3	7.57	13	4.6%	-0.16 [-0.90, 0.59]
Magner 2023 week 36 (ADOS)	-0.2	4.26	15	-0.5	5.05	13	4.6%	0.06 [-0.68, 0.81]
Ou 2022 week 12 (OARS-4)	-0.64	0.5	51	-0.24	0.46	43	14.3%	-0.82 [-1.25, -0.40]
Ou 2022 week 4 (OARS-4)	-0.25	0.43	51	-0.12	0.39	43	15.3%	-0.31 [-0.72, 0.10]
Ou 2022 week 8 (OARS-4)	-0.43	0.43	51	-0.16	0.46	43	14.8%	-0.60 [-1.02, -0.19]
Singh 2014 week 10 (OACIS-I)	3.08	0.89	26	3.79	0.58	14	5.5%	-0.87 [-1.55, -0.19]
Singh 2014 week 10 (OACIS-S)	-1.05	4.64	26	-0.23	4.38	14	6.0%	-0.18 [-0.83, 0.47]
Singh 2014 week 18 (OACIS-I)	3.08	1.02	26	3.92	0.47	14	5.4%	-0.94 [-1.63, -0.26]
Singh 2014 week 18 (OACIS-S)	-1.05	5.05	26	-0.09	4.34	14	6.0%	-0.20 [-0.85, 0.46]
Singh 2014 week 4 (OACIS-I)	3.38	0.85	26	3.71	0.61	14	5.9%	-0.42 [-1.07, 0.24]
Singh 2014 week 4 (OACIS-S)	-0.74	4.49	26	-0.3	4.08	14	6.1%	-0.10 [-0.75, 0.55]
Zimmerman 2021 week 15 (OACIS-I)	0.41	0.71	17	0.47	0.51	17	5.7%	-0.09 [-0.77, 0.58]
Zimmerman 2021 week 7 (OACIS-I)	0.35	0.7	17	0.47	0.62	17	5.6%	-0.18 [-0.85, 0.50]
Total (95% CI)			373			273	100.0%	-0.43 [-0.59, -0.27]

Heterogeneity: Chi² = 13.87, df = 12 (P = 0.31); I² = 13%
Test for overall effect: Z = 5.24 (P < 0.00001)

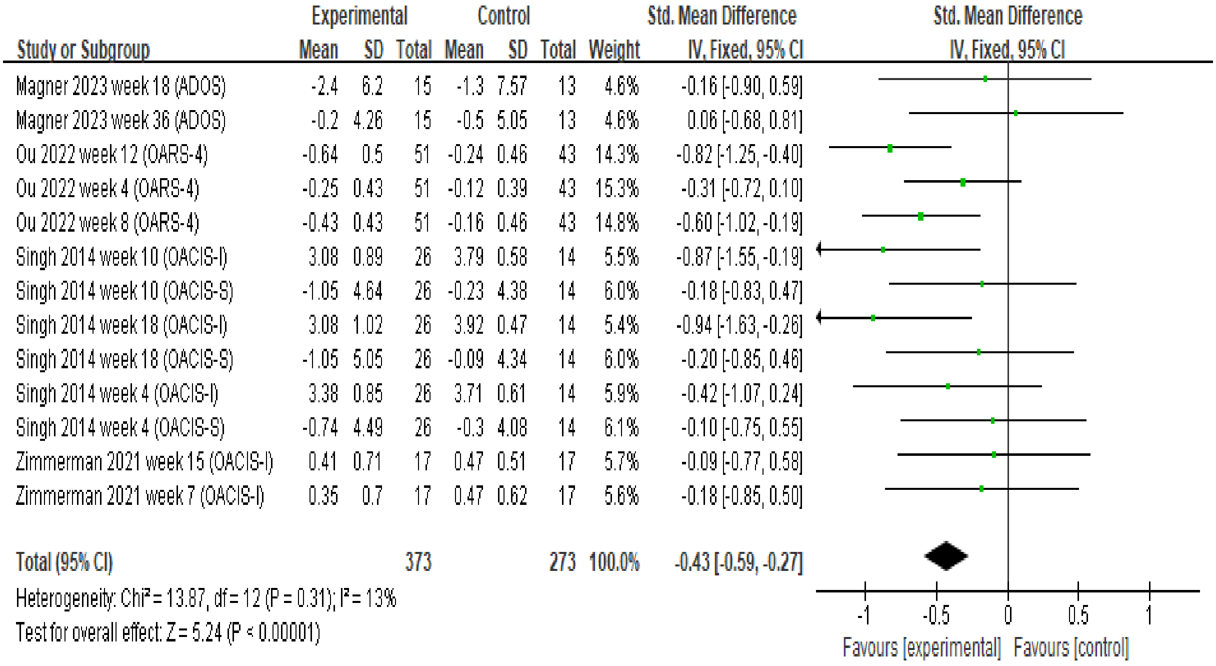

Supplementary Figure 1.4: Forest plot of social interaction with autism spectrum disorder

Study or Subgroup	Experimental Mean	Experimental SD	Experimental Total	Control Mean	Control SD	Control Total	Weight	Std. Mean Difference IV, Fixed, 95% CI
Magner 2023 week 18 (ADOS)	-0.7	1.16	15	-1.3	1.8	13	2.4%	0.39 [-0.36, 1.14]
Magner 2023 week 36 (ADOS)	0.2	1.55	15	-0.1	1.8	13	2.4%	0.17 [-0.57, 0.92]
Ou 2022 week 12 (OARS-4)	-0.37	0.5	51	-0.05	0.46	43	7.8%	-0.66 [-1.08, -0.24]
Ou 2022 week 4 (OARS-4)	-0.06	0.43	51	0.08	0.46	43	8.1%	-0.31 [-0.72, 0.10]
Ou 2022 week 8 (OARS-4)	-0.21	0.57	51	-0.01	0.46	43	8.1%	-0.38 [-0.79, 0.03]
Politte 2020 week 12 (SRS)	0.1	32.33	24	0	20.09	24	4.2%	0.00 [-0.56, 0.57]
Politte 2020 week 4 (SRS)	-1.6	32.33	24	1.6	20.09	24	4.2%	-0.12 [-0.68, 0.45]
Politte 2020 week 8 (SRS)	-1.4	32.33	24	1.5	20.09	24	4.2%	-0.11 [-0.67, 0.46]
Singh 2014 week 10 (OACIS-I) N	3.58	0.76	26	3.92	0.28	14	3.1%	-0.52 [-1.18, 0.14]
Singh 2014 week 10 (OACIS-I) V	3.16	0.83	26	3.36	0.84	14	3.2%	-0.24 [-0.89, 0.42]
Singh 2014 week 10 (OACIS-S) N	-0.37	4.69	26	-0.04	3.7	14	3.2%	-0.07 [-0.72, 0.58]
Singh 2014 week 10 (OACIS-S) V	-0.72	5.2	26	-0.32	6.21	14	3.2%	-0.07 [-0.72, 0.58]
Singh 2014 week 18 (OACIS-I) N	3.54	0.764	26	4.07	0.27	14	3.0%	-0.81 [-1.49, -0.14]
Singh 2014 week 18 (OACIS-I) V	2.96	0.92	26	3.86	0.53	14	2.8%	-1.09 [-1.79, -0.39]
Singh 2014 week 18 (OACIS-S) N	-0.41	5	26	0.16	3.55	14	3.2%	-0.12 [-0.77, 0.53]
Singh 2014 week 18 (OACIS-S) V	-0.91	5.25	26	0.18	5.05	14	3.2%	-0.21 [-0.86, 0.45]
Singh 2014 week 4 (OACIS-I) N	3.69	0.62	26	3.93	0.27	14	3.1%	-0.45 [-1.10, 0.21]
Singh 2014 week 4 (OACIS-I) V	3.15	0.92	26	3.57	0.76	14	3.1%	-0.47 [-1.13, 0.18]
Singh 2014 week 4 (OACIS-S) N	-0.25	4.59	26	0.02	3.67	14	3.2%	-0.06 [-0.71, 0.59]
Singh 2014 week 4 (OACIS-S) V	-0.72	5.76	26	-0.1	5.84	14	3.2%	-0.10 [-0.76, 0.55]
Zimmerman 2021 week 15 (OACIS-I) N	0.29	0.69	17	0.24	0.44	17	3.0%	0.08 [-0.59, 0.76]
Zimmerman 2021 week 15 (OACIS-I) V	0.41	0.71	17	0.79	0.77	17	2.9%	-0.50 [-1.19, 0.18]
Zimmerman 2021 week 15 (SRS) C	-4.25	4.77	12	-3.92	7.27	12	2.1%	-0.05 [-0.85, 0.75]
Zimmerman 2021 week 15 (SRS) I	-9.75	12.34	12	-9	12.2	12	2.1%	-0.06 [-0.86, 0.74]
Zimmerman 2021 week 7 (OACIS-I) N	0.18	0.53	17	0.29	0.47	17	3.0%	-0.21 [-0.89, 0.46]
Zimmerman 2021 week 7 (OACIS-I) V	0.24	0.56	17	0.59	0.62	17	2.9%	-0.58 [-1.27, 0.11]
Zimmerman 2021 week 7 (SRS) C	0	5.62	15	-3.47	5.9	15	2.5%	0.59 [-0.15, 1.32]
Zimmerman 2021 week 7 (SRS) I	1.8	12.18	15	-7.07	11.68	15	2.5%	0.72 [-0.02, 1.47]
Total (95% CI)			689			517	100.0%	-0.24 [-0.35, -0.12]

Heterogeneity: Chi² = 34.74, df = 27 (P = 0.15); I² = 22%
Test for overall effect: Z = 3.96 (P < 0.0001)

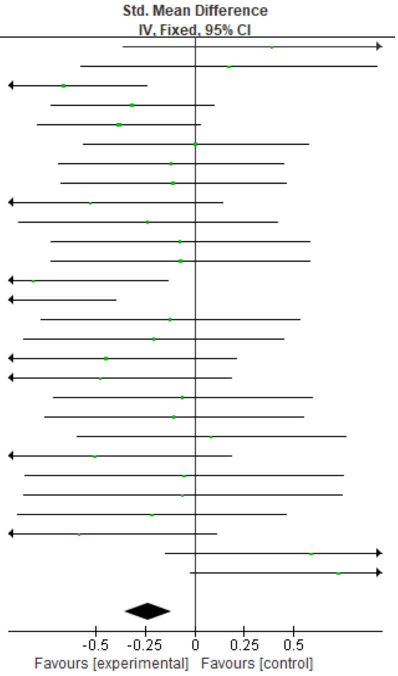

Supplementary Figure 1.5: Forest plot of social communication with autism spectrum disorder

EXCLI Journal 2025;24:542-557 – ISSN 1611-2156
Received: January 31, 2025, accepted: April 02, 2025, published: April 07, 2025

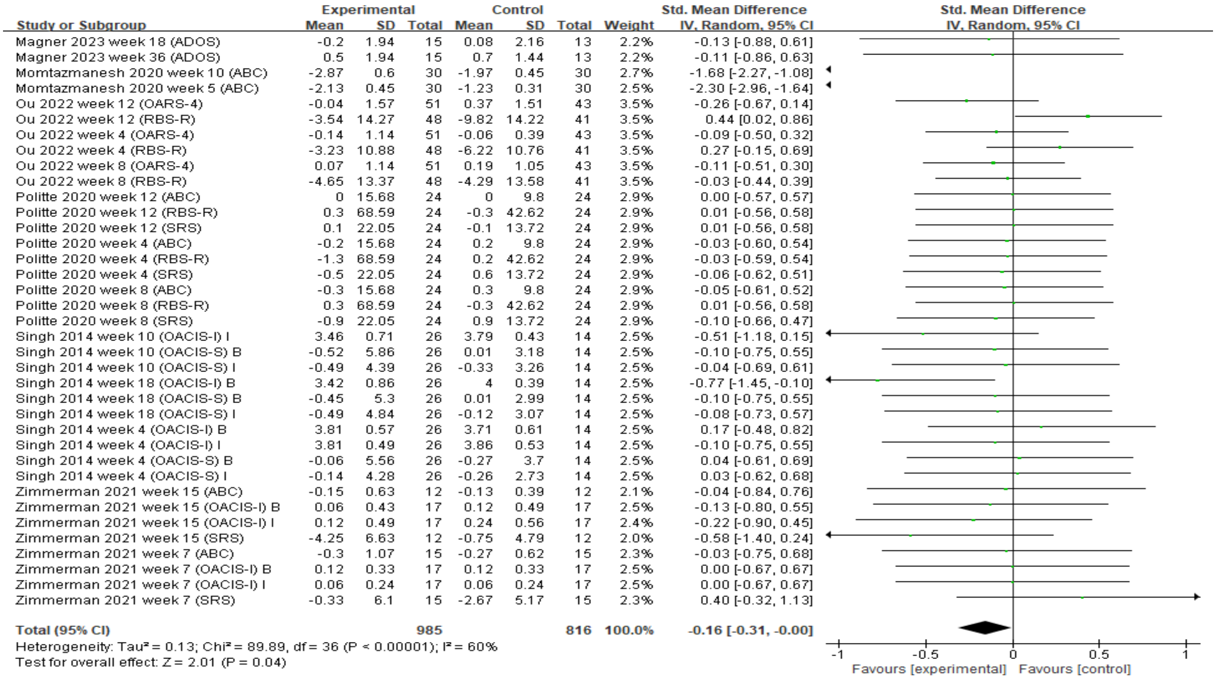

Supplementary Figure 1.6: Forest plot of restricted interests and repetitive behavior with autism spectrum disorder

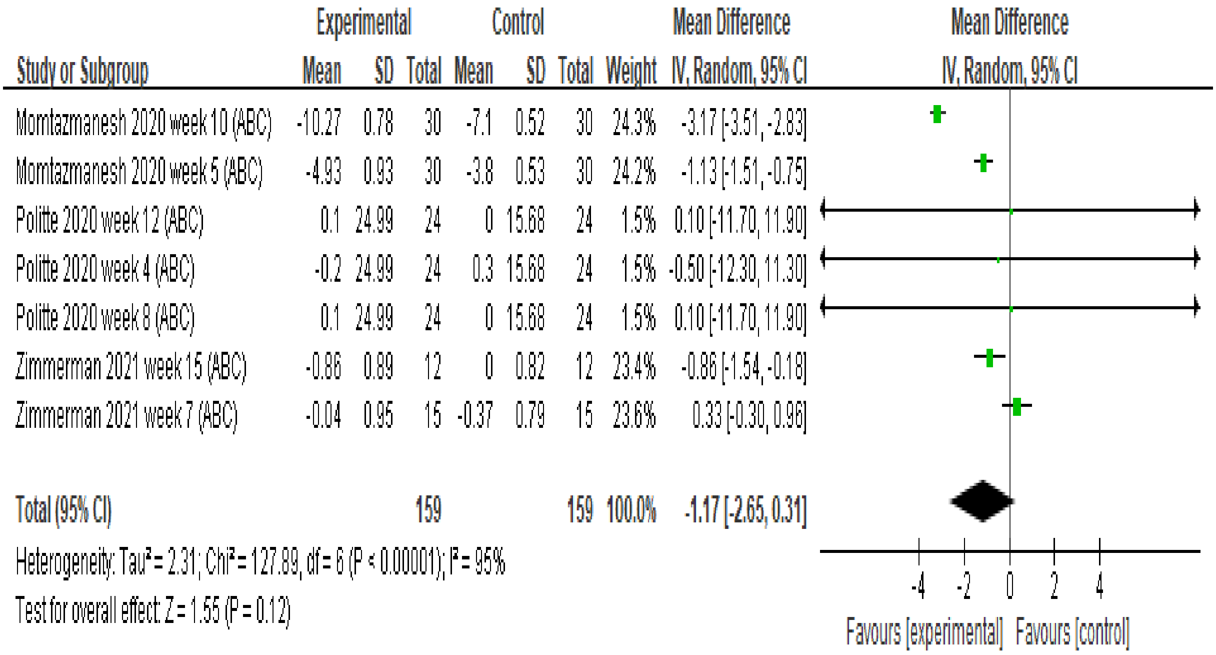

Supplementary Figure 1.7: Forest plot of irritability with autism spectrum disorder

EXCLI Journal 2025;24:542-557 – ISSN 1611-2156
Received: January 31, 2025, accepted: April 02, 2025, published: April 07, 2025

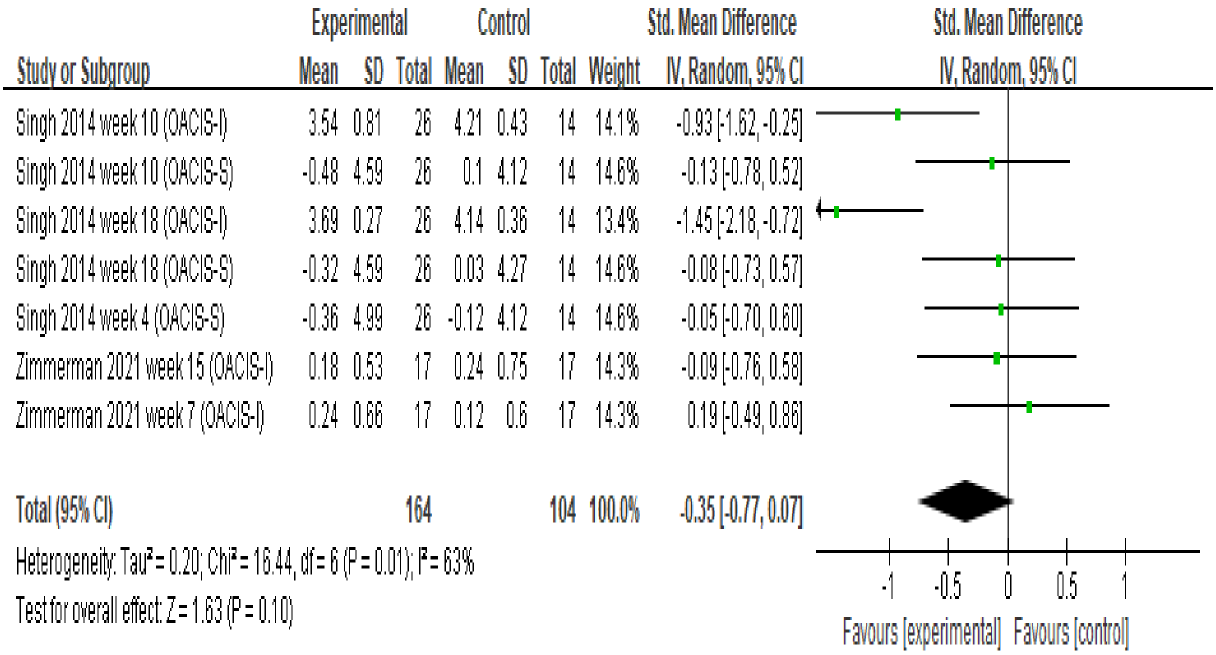

Supplementary Figure 1.8: Forest plot of anxiety with autism spectrum disorder

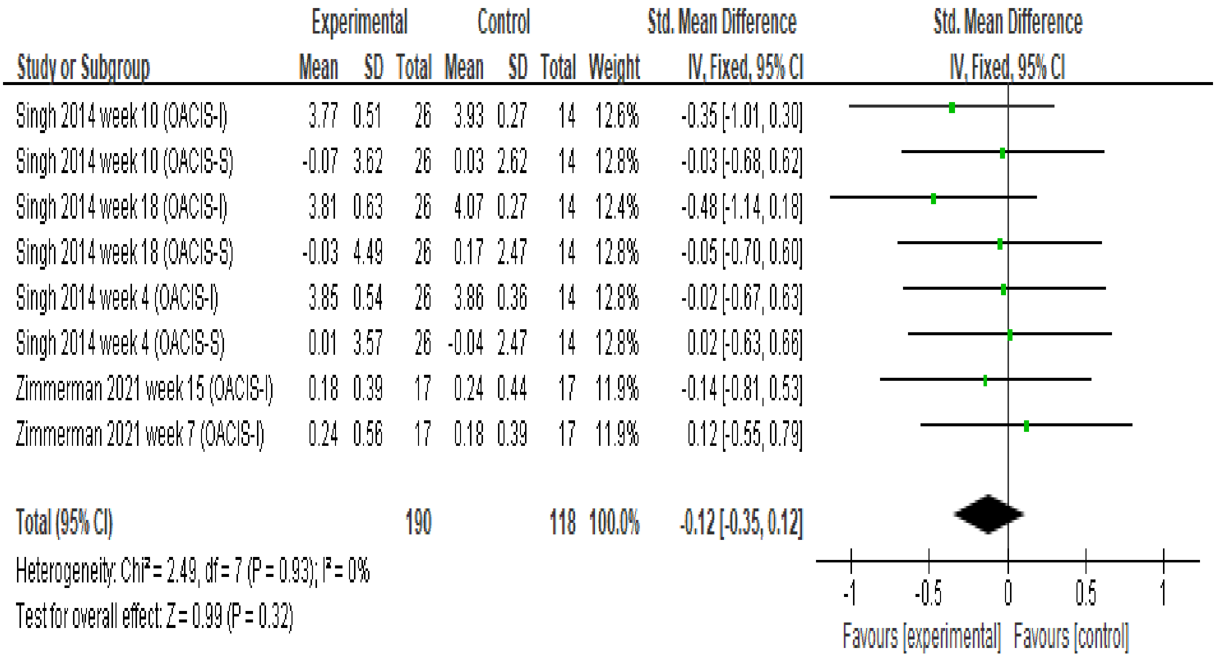

Supplementary Figure 1.9: Forest plot of sensory sensitivity with autism spectrum disorder

EXCLI Journal 2025;24:542-557 – ISSN 1611-2156
Received: January 31, 2025, accepted: April 02, 2025, published: April 07, 2025

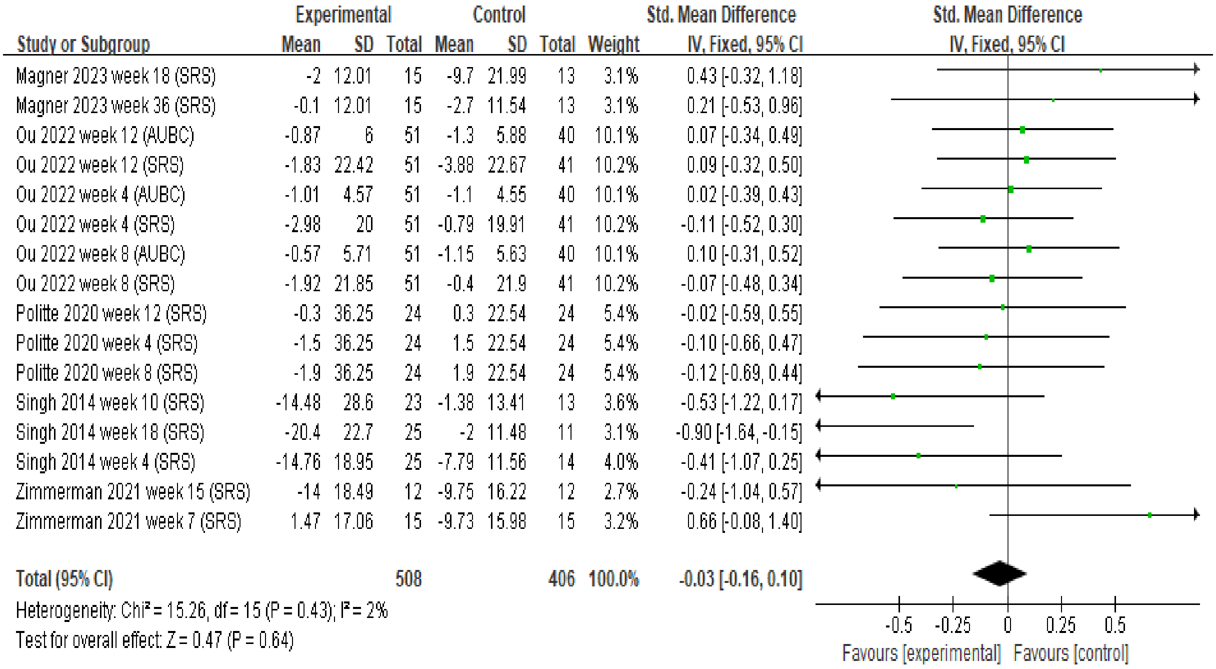

Supplementary Figure 1.10: Forest plot of total social skills with autism spectrum disorder

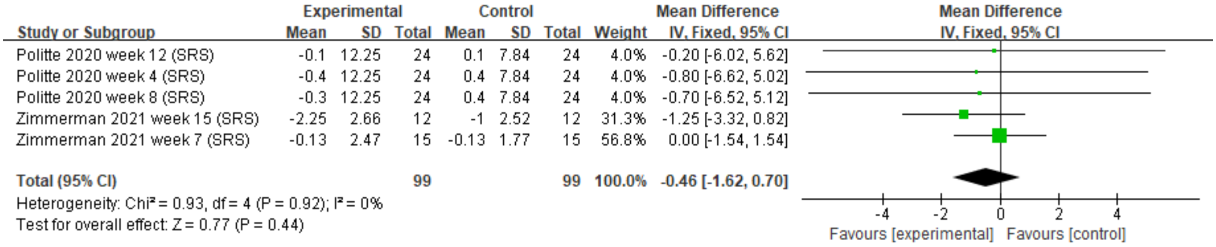

Supplementary Figure 1.11: Forest plot of social awareness with autism spectrum disorder

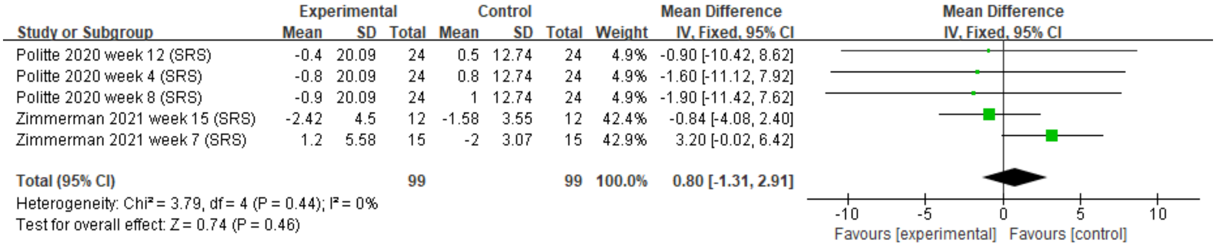

Supplementary Figure 1.12: Forest plot of social cognition with autism spectrum disorder

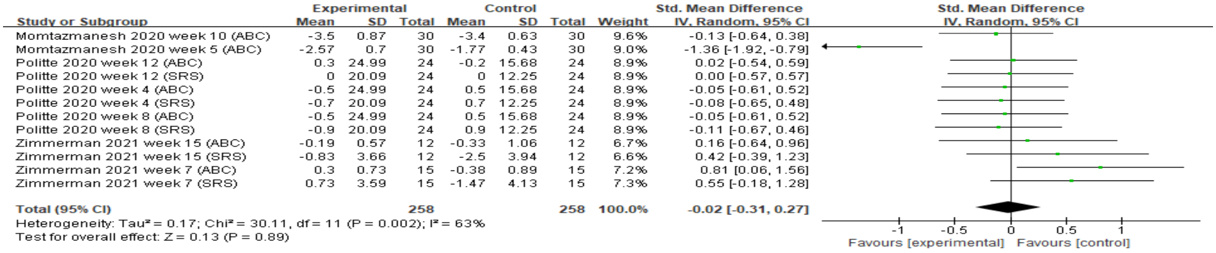

Supplementary Figure 1.13: Forest plot of social motivation with autism spectrum disorder

EXCLI Journal 2025;24:542-557 – ISSN 1611-2156
Received: January 31, 2025, accepted: April 02, 2025, published: April 07, 2025

Supplementary Figure 2: Forest plot for adverse effects

Study or Subgroup	Experimental Events	Experimental Total	Control Events	Control Total	Weight	Risk Ratio M-H, Fixed, 95% CI
Politte 2020	1	24	0	24	8.0%	3.00 [0.13, 70.16]
Singh 2014	2	26	4	14	82.8%	0.27 [0.06, 1.29]
Zimmerman 2021	2	32	0	23	9.2%	3.64 [0.18, 72.35]
**Total (95% CI)**		**82**		**61**	**100.0%**	**0.80 [0.27, 2.33]**
Total events	5		4			
Heterogeneity: Chi² = 3.51, df = 2 (P = 0.17); I² = 43%						
Test for overall effect: Z = 0.41 (P = 0.68)						

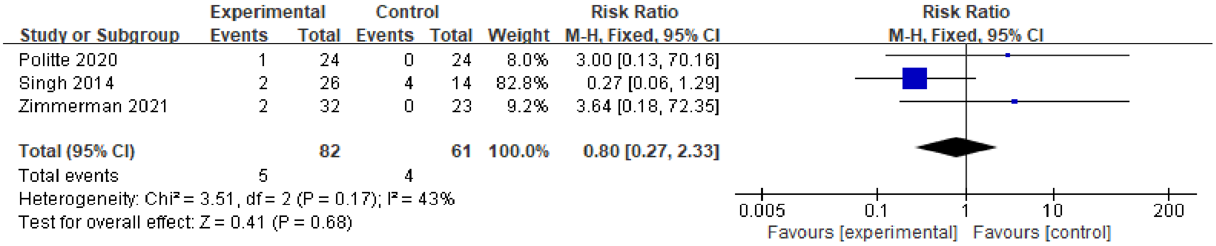

Supplementary Figure 2.1: Forest plot for adverse effects of insomnia

Study or Subgroup	Experimental Events	Experimental Total	Control Events	Control Total	Weight	Risk Ratio M-H, Fixed, 95% CI
Politte 2020	3	24	4	24	76.6%	0.75 [0.19, 3.00]
Singh 2014	3	26	0	14	12.3%	3.89 [0.22, 70.34]
Zimmerman 2021	2	32	0	23	11.1%	3.64 [0.18, 72.35]
**Total (95% CI)**		**82**		**61**	**100.0%**	**1.46 [0.49, 4.34]**
Total events	8		4			
Heterogeneity: Chi² = 1.68, df = 2 (P = 0.43); I² = 0%						
Test for overall effect: Z = 0.67 (P = 0.50)						

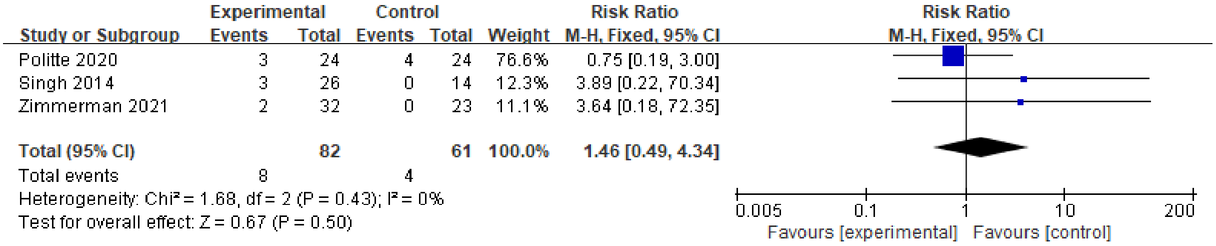

Supplementary Figure 2.2: Forest plot for adverse effects of irritability

Study or Subgroup	Experimental Events	Experimental Total	Control Events	Control Total	Weight	Risk Ratio M-H, Fixed, 95% CI
Momtazmanesh 2020	4	30	3	30	56.6%	1.33 [0.33, 5.45]
Politte 2020	1	24	1	24	18.9%	1.00 [0.07, 15.08]
Singh 2014	3	26	1	14	24.5%	1.62 [0.18, 14.12]
**Total (95% CI)**		**80**		**68**	**100.0%**	**1.34 [0.45, 3.95]**
Total events	8		5			
Heterogeneity: Chi² = 0.07, df = 2 (P = 0.96); I² = 0%						
Test for overall effect: Z = 0.53 (P = 0.60)						

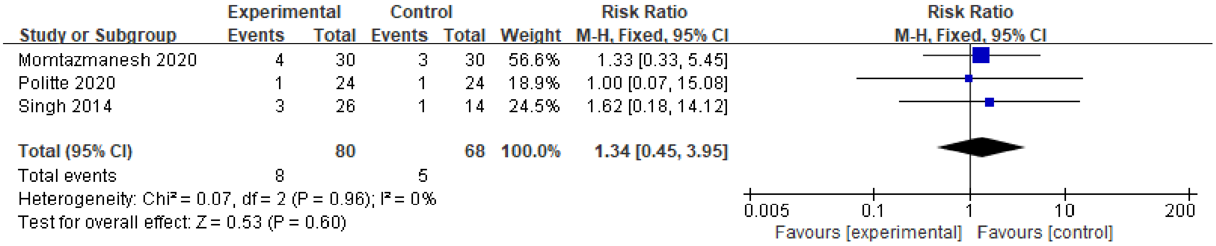

Supplementary Figure 2.3: Forest plot for adverse effects of headache

EXCLI Journal 2025;24:542-557 – ISSN 1611-2156
Received: January 31, 2025, accepted: April 02, 2025, published: April 07, 2025

Supplementary Figure 3: Leave-one-out sensitivity analyses

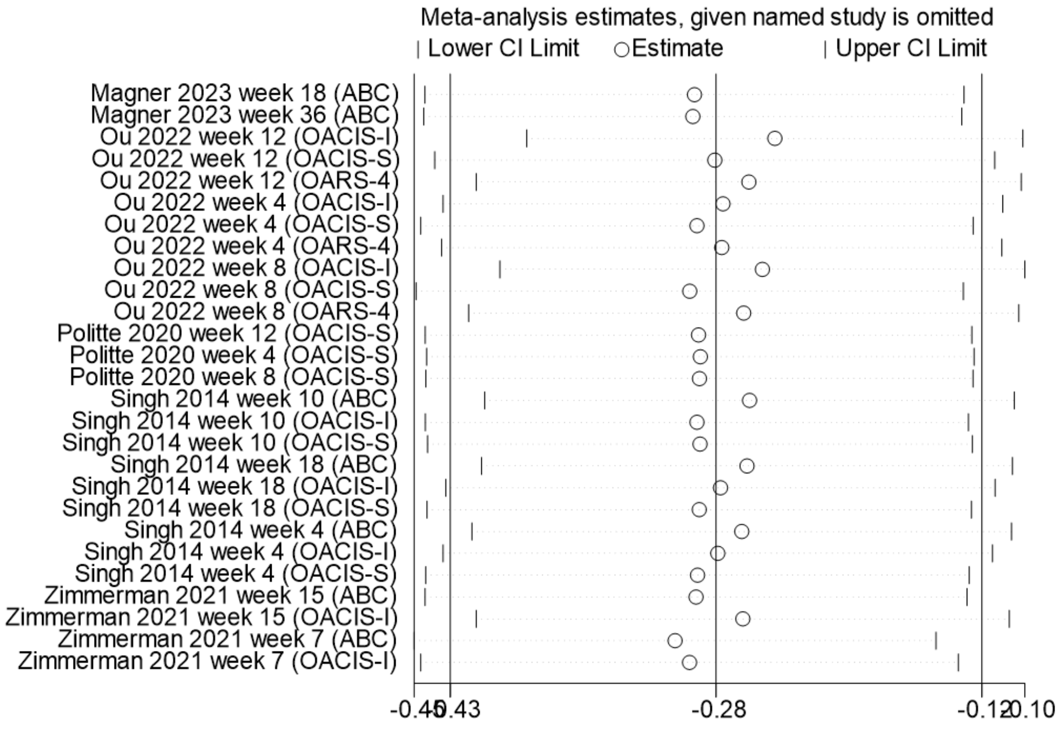

Supplementary Figure 3.1: Leave-one-out sensitivity analyses for total symptoms

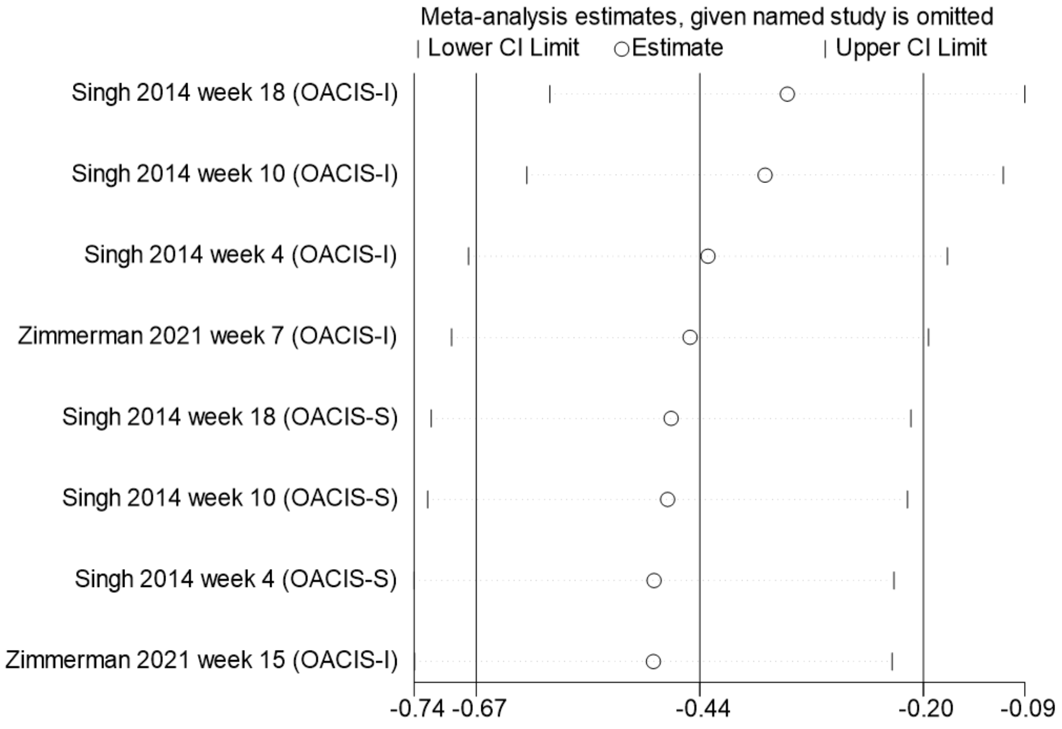

Supplementary Figure 3.2: Leave-one-out sensitivity analyses for aberrant behavior

EXCLI Journal 2025;24:542-557 – ISSN 1611-2156
Received: January 31, 2025, accepted: April 02, 2025, published: April 07, 2025

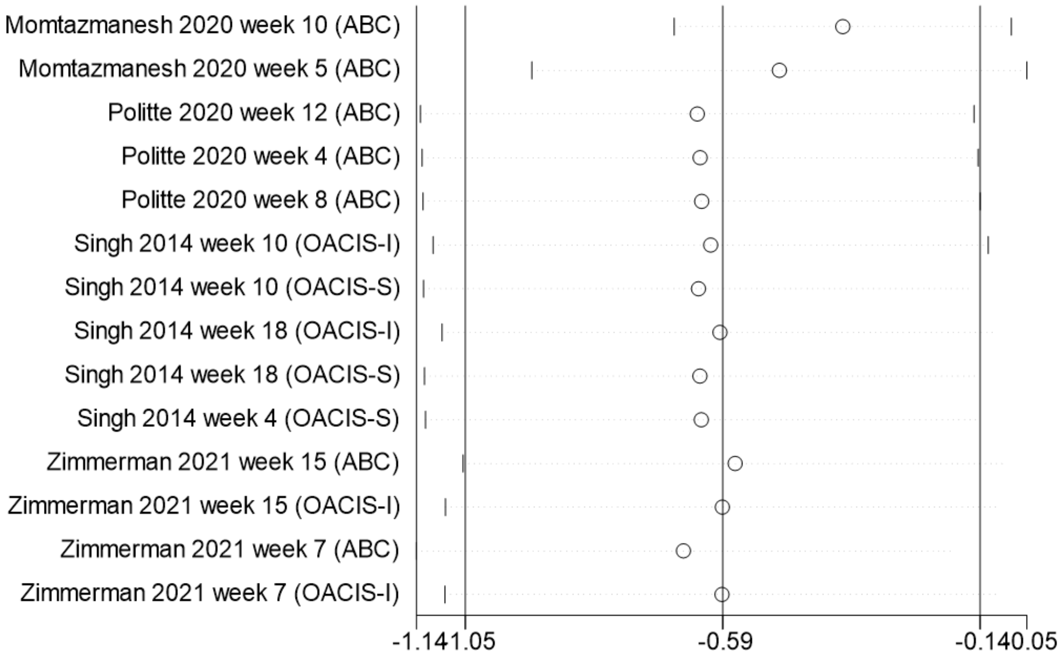

Supplementary Figure 3.3: Leave-one-out sensitivity analyses for hyperactivity

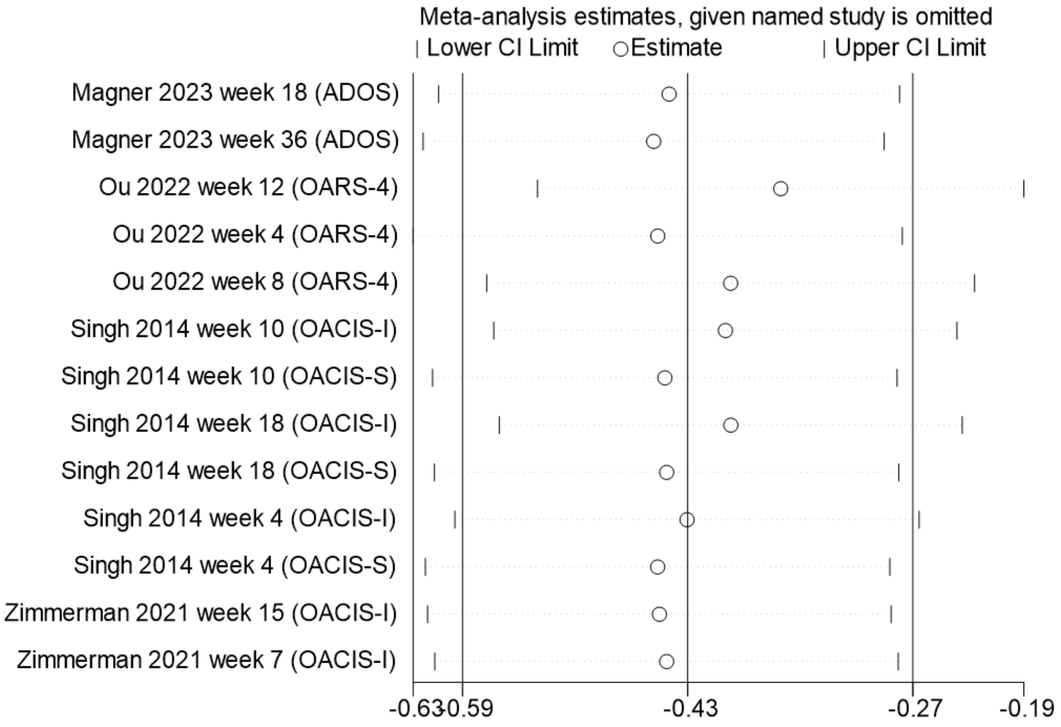

Supplementary Figure 3.4: Leave-one-out sensitivity analyses for social interaction

EXCLI Journal 2025;24:542-557 – ISSN 1611-2156
Received: January 31, 2025, accepted: April 02, 2025, published: April 07, 2025

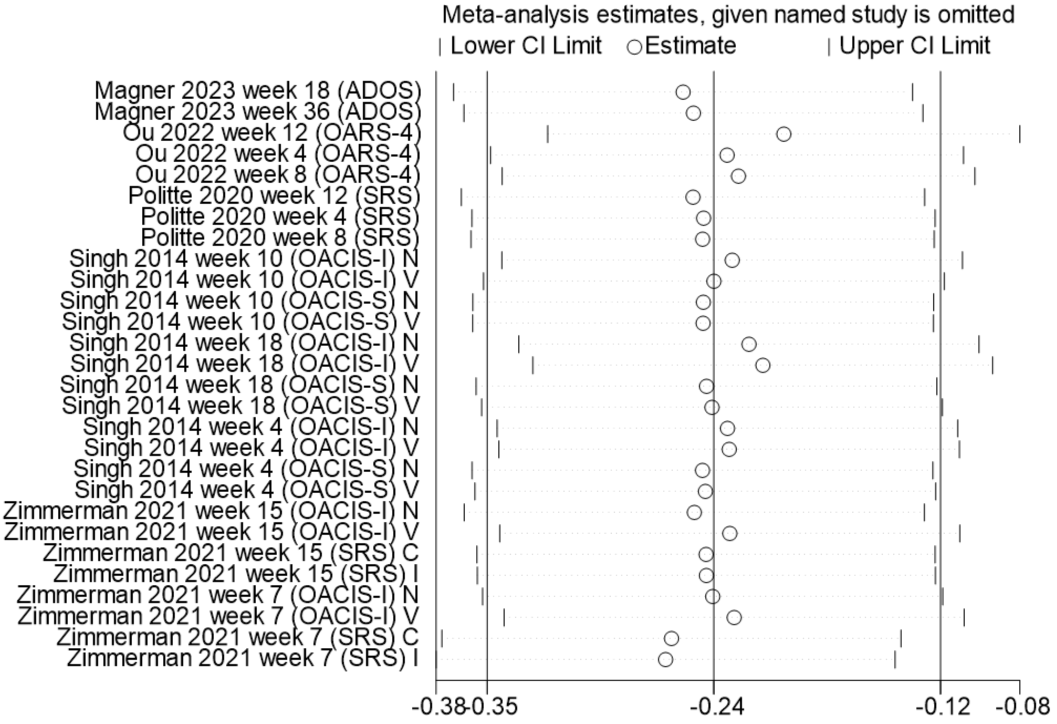

Supplementary Figure 3.5: Leave-one-out sensitivity analyses for social communication

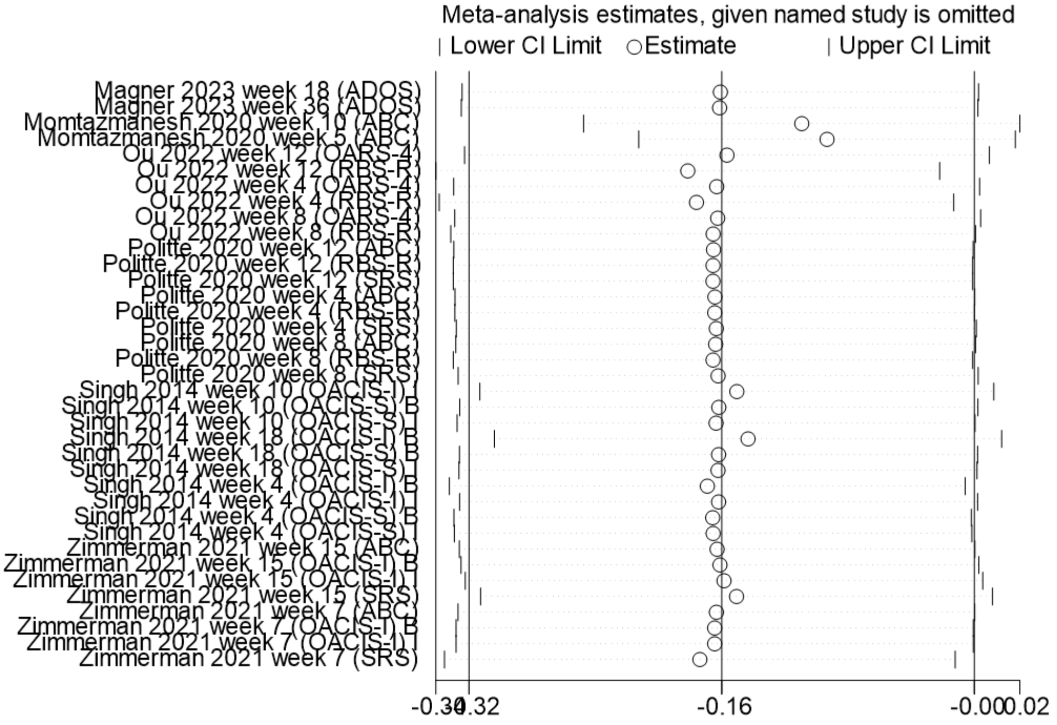

Supplementary Figure 3.6: Leave-one-out sensitivity analyses for restricted interests and repetitive behavior

EXCLI Journal 2025;24:542-557 – ISSN 1611-2156
Received: January 31, 2025, accepted: April 02, 2025, published: April 07, 2025

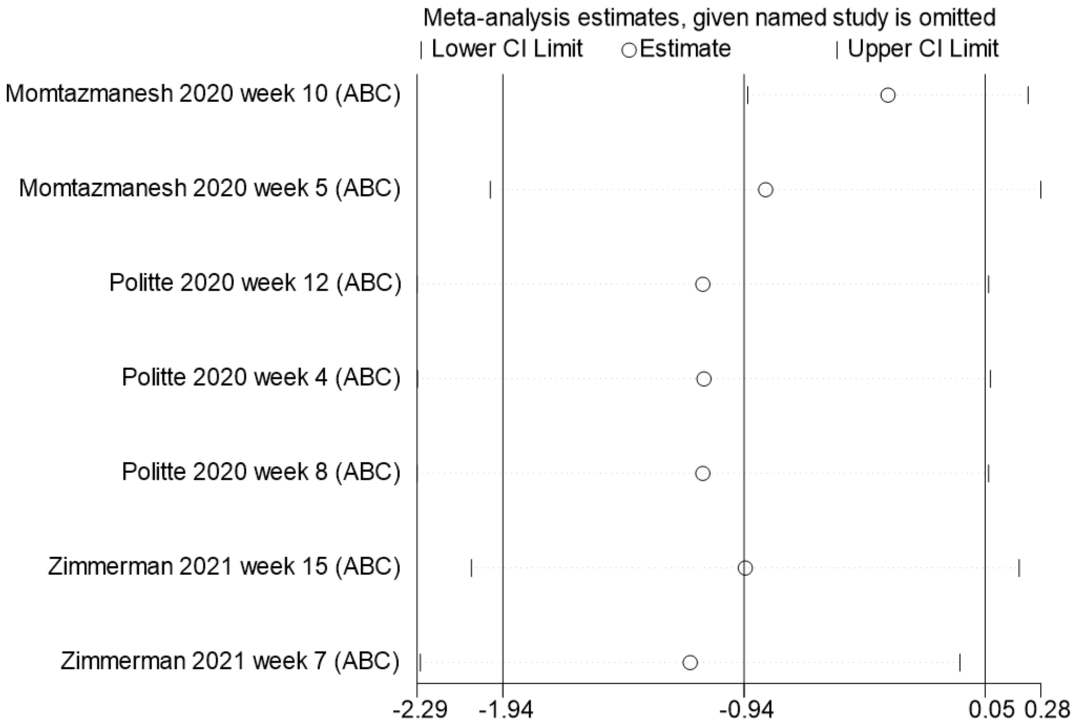

Supplementary Figure 3.7: Leave-one-out sensitivity analyses for irritability

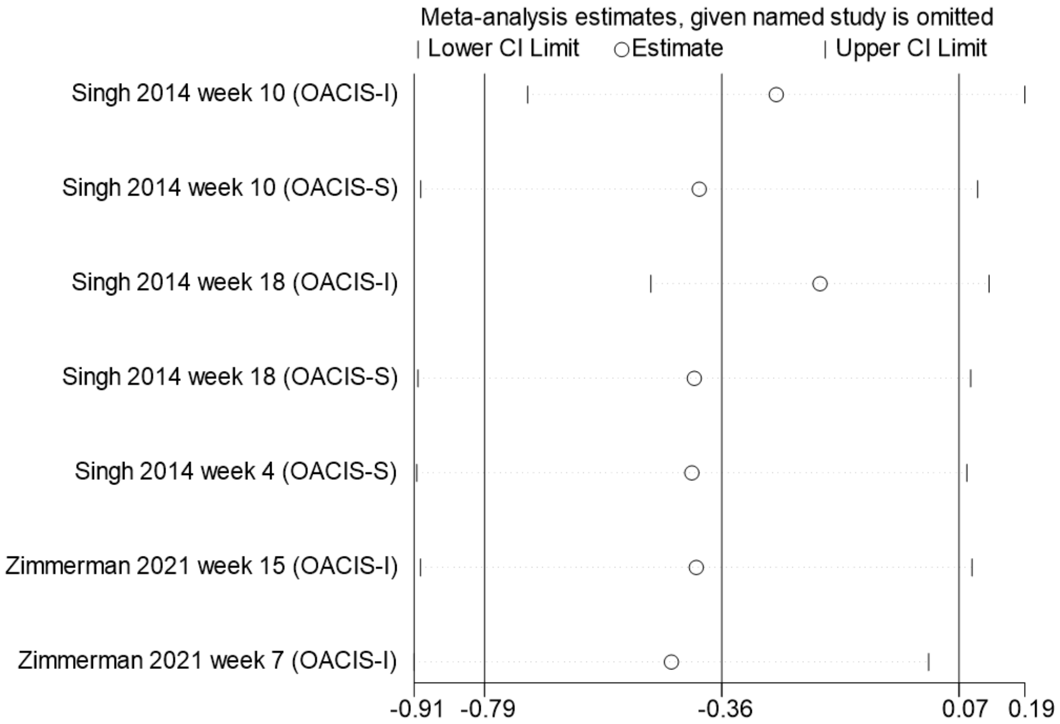

Supplementary Figure 3.8: Leave-one-out sensitivity analyses for anxiety

EXCLI Journal 2025;24:542-557 – ISSN 1611-2156
Received: January 31, 2025, accepted: April 02, 2025, published: April 07, 2025

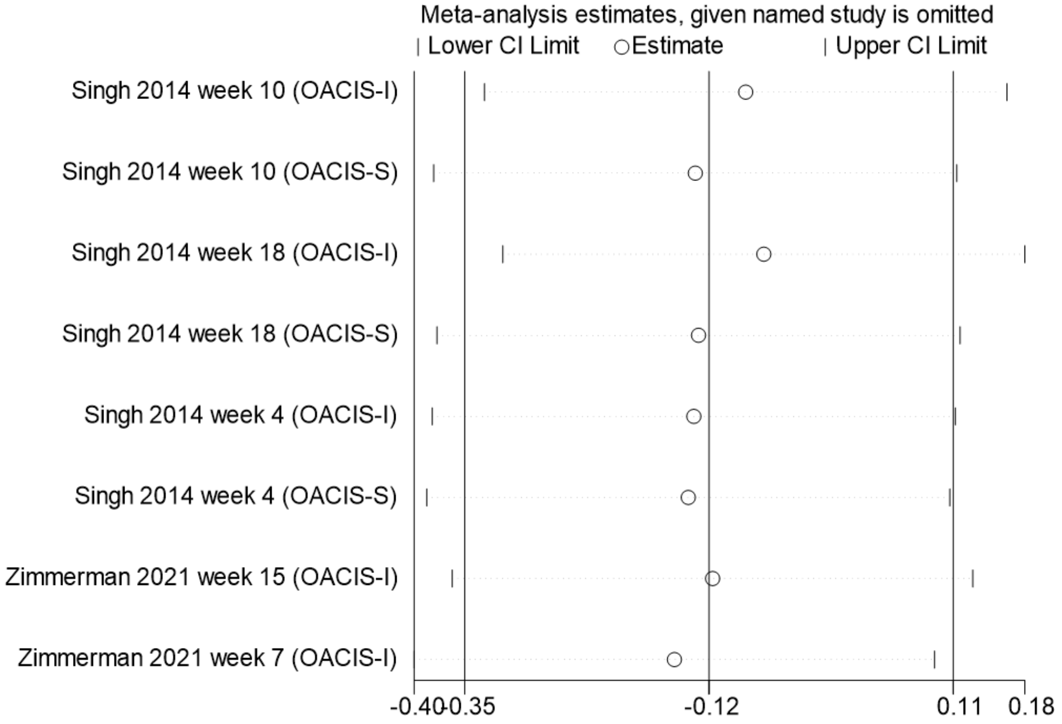

Supplementary Figure 3.9: Leave-one-out sensitivity analyses for sensory sensitivity

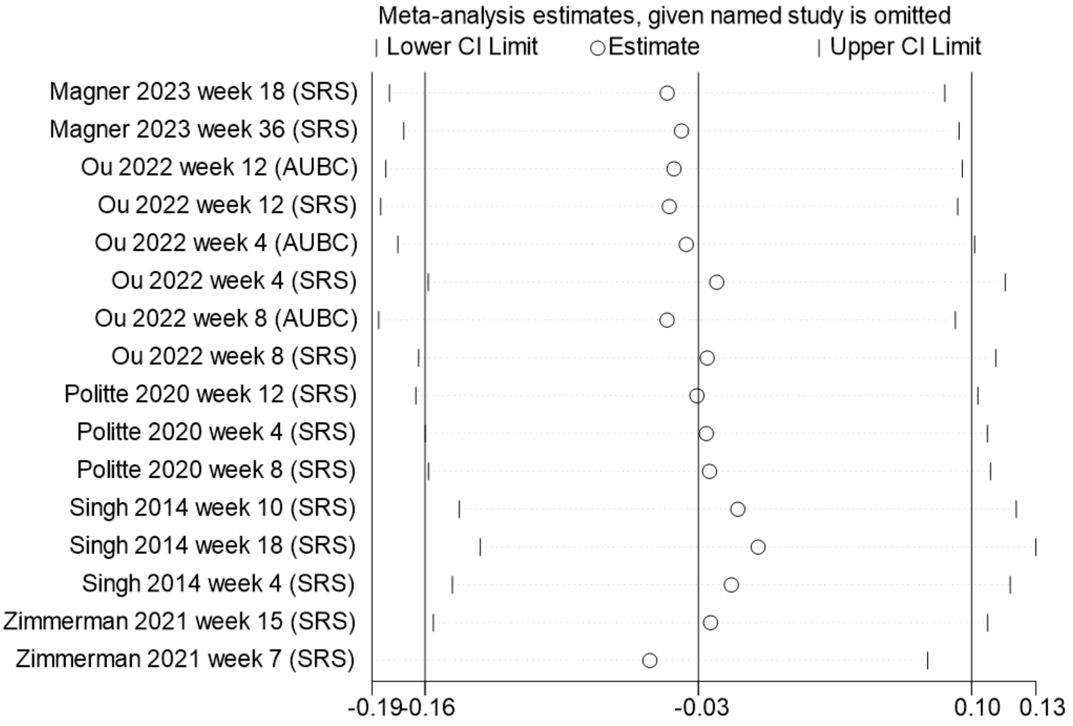

Supplementary Figure 3.10: Leave-one-out sensitivity analyses for total social skills

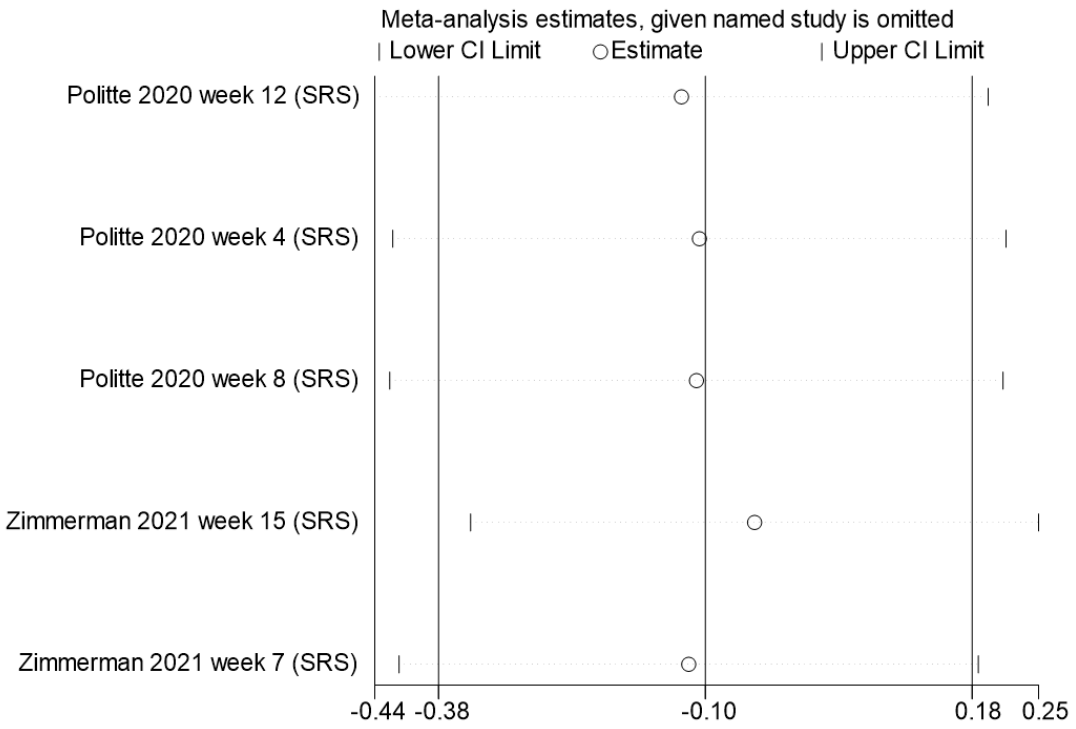

Supplementary Figure 3.11: Leave-one-out sensitivity analyses for social awareness

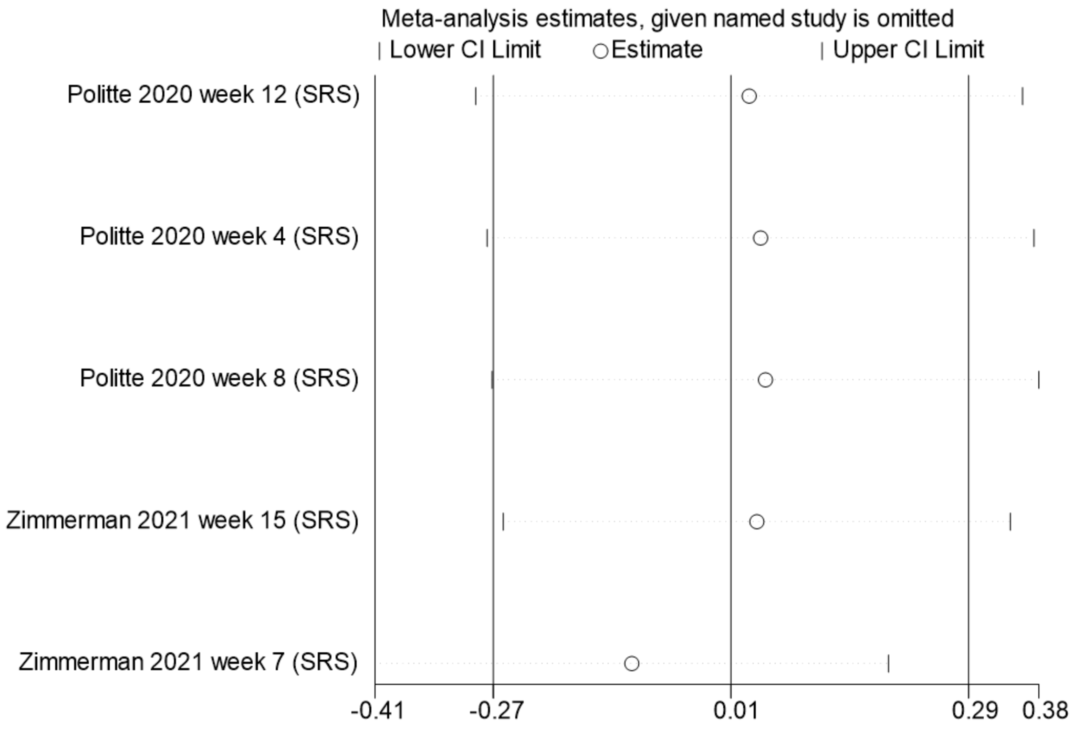

Supplementary Figure 3.12: Leave-one-out sensitivity analyses for social cognition

EXCLI Journal 2025;24:542-557 – ISSN 1611-2156
Received: January 31, 2025, accepted: April 02, 2025, published: April 07, 2025

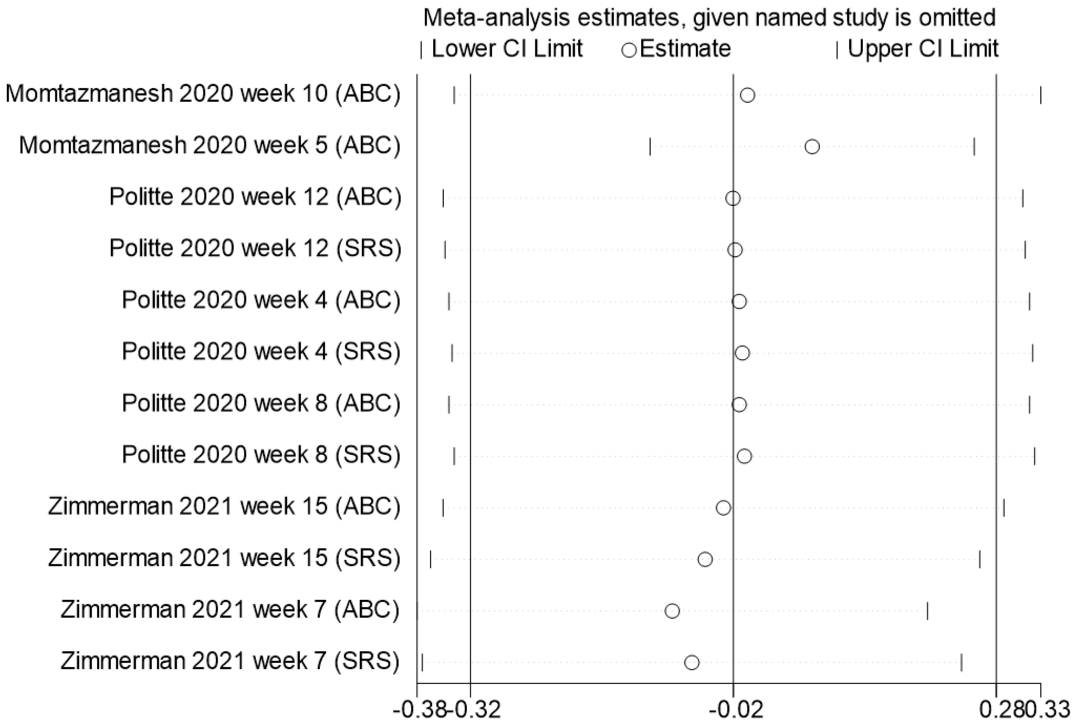

Supplementary Figure 3.13: Leave-one-out sensitivity analyses for social motivation

EXCLI Journal 2025;24:542-557 – ISSN 1611-2156
Received: January 31, 2025, accepted: April 02, 2025, published: April 07, 2025

Supplementary Figure 4: Subgroup analyses of each outcome according to national income levels, ages, measurement tools, assessors of outcome indicators and intervention duration

Supplementary Figure 4.1: Subgroup analyses of different national income levels

Study or Subgroup	Experimental Mean	Experimental SD	Experimental Total	Control Mean	Control SD	Control Total	Weight	Std. Mean Difference IV, Random, 95% CI
**5.1.1 High-income economies**								
Magner 2023 week 18 (ABC)	-4.9	16.27	15	-8.1	22.35	13	2.7%	0.16 [-0.58, 0.90]
Magner 2023 week 36 (ABC)	1.6	12.39	15	-1	14.06	13	2.7%	0.19 [-0.55, 0.94]
Politte 2020 week 12 (OACIS-S)	-0.02	2.94	24	0	1.47	24	3.7%	-0.01 [-0.57, 0.56]
Politte 2020 week 4 (OACIS-S)	-0.04	2.94	24	0.04	1.47	24	3.7%	-0.03 [-0.60, 0.53]
Politte 2020 week 8 (OACIS-S)	-0.03	2.94	24	0.02	1.47	24	3.7%	-0.02 [-0.59, 0.54]
Singh 2014 week 10 (ABC)	-19.61	27.91	22	3.31	12.62	13	2.8%	-0.95 [-1.68, -0.23]
Singh 2014 week 10 (OACIS-I)	3.88	0.33	26	3.86	0.36	14	3.2%	0.06 [-0.59, 0.71]
Singh 2014 week 10 (OACIS-S)	-0.07	3.42	26	-0.1	2.99	14	3.2%	0.01 [-0.64, 0.66]
Singh 2014 week 18 (ABC)	-21.44	21.7	25	-2	14.52	10	2.6%	-0.95 [-1.72, -0.18]
Singh 2014 week 18 (OACIS-I)	3.81	0.4	26	3.93	0.27	14	3.2%	-0.33 [-0.98, 0.33]
Singh 2014 week 18 (OACIS-S)	0.04	3.42	26	-0.03	2.81	14	3.2%	0.02 [-0.63, 0.67]
Singh 2014 week 4 (ABC)	-12.69	21.26	26	0.77	6.63	13	3.0%	-0.74 [-1.43, -0.05]
Singh 2014 week 4 (OACIS-I)	4.04	0.2	26	4.14	0.53	14	3.2%	-0.28 [-0.93, 0.37]
Singh 2014 week 4 (OACIS-S)	0	3.62	26	-0.17	2.96	14	3.2%	0.05 [-0.60, 0.70]
Zimmerman 2021 week 15 (ABC)	0.29	0.59	17	0.24	0.56	17	3.1%	0.08 [-0.59, 0.76]
Zimmerman 2021 week 15 (OACIS-I)	-19.5	18.47	12	-2.92	16.1	12	2.3%	-0.92 [-1.77, -0.07]
Zimmerman 2021 week 7 (ABC)	-2	18.68	15	-11	16.99	15	2.8%	0.49 [-0.24, 1.22]
Zimmerman 2021 week 7 (OACIS-I)	0.29	0.69	17	0.18	0.39	17	3.1%	0.19 [-0.48, 0.87]
**Subtotal (95% CI)**			**392**			**279**	**55.4%**	**-0.14 [-0.32, 0.04]**
Heterogeneity: Tau² = 0.04; Chi² = 22.96, df = 17 (P = 0.15); I² = 26%								
Test for overall effect: Z = 1.49 (P = 0.14)								
**5.1.2 Upper-middle-income economies**								
Ou 2022 week 12 (OACIS-I)	2.89	0.63	49	3.51	0.58	42	4.8%	-1.01 [-1.45, -0.57]
Ou 2022 week 12 (OACIS-S)	-0.17	0.71	51	0.01	0.79	43	5.0%	-0.24 [-0.65, 0.17]
Ou 2022 week 12 (OARS-4)	-0.33	0.36	51	-0.09	0.39	43	5.0%	-0.64 [-1.05, -0.22]
Ou 2022 week 4 (OACIS-I)	3.61	0.56	49	3.79	0.52	42	5.0%	-0.33 [-0.74, 0.09]
Ou 2022 week 4 (OACIS-S)	0.13	0.71	51	0.17	0.72	43	5.0%	-0.06 [-0.46, 0.35]
Ou 2022 week 4 (OARS-4)	-0.12	0.36	51	-0.01	0.33	43	5.0%	-0.31 [-0.72, 0.09]
Ou 2022 week 8 (OACIS-I)	3.26	0.56	49	3.71	0.52	42	4.8%	-0.82 [-1.25, -0.39]
Ou 2022 week 8 (OACIS-S)	0.1	0.71	51	0.09	0.72	43	5.0%	0.01 [-0.39, 0.42]
Ou 2022 week 8 (OARS-4)	-0.23	0.36	51	-0.03	0.33	43	5.0%	-0.57 [-0.99, -0.16]
**Subtotal (95% CI)**			**453**			**384**	**44.6%**	**-0.43 [-0.66, -0.21]**
Heterogeneity: Tau² = 0.07; Chi² = 20.62, df = 8 (P = 0.008); I² = 61%								
Test for overall effect: Z = 3.83 (P = 0.0001)								
**Total (95% CI)**			**845**			**663**	**100.0%**	**-0.27 [-0.42, -0.12]**
Heterogeneity: Tau² = 0.07; Chi² = 51.11, df = 26 (P = 0.002); I² = 49%								
Test for overall effect: Z = 3.56 (P = 0.0004)								
Test for subgroup differences: Chi² = 4.02, df = 1 (P = 0.04), I² = 75.1%								

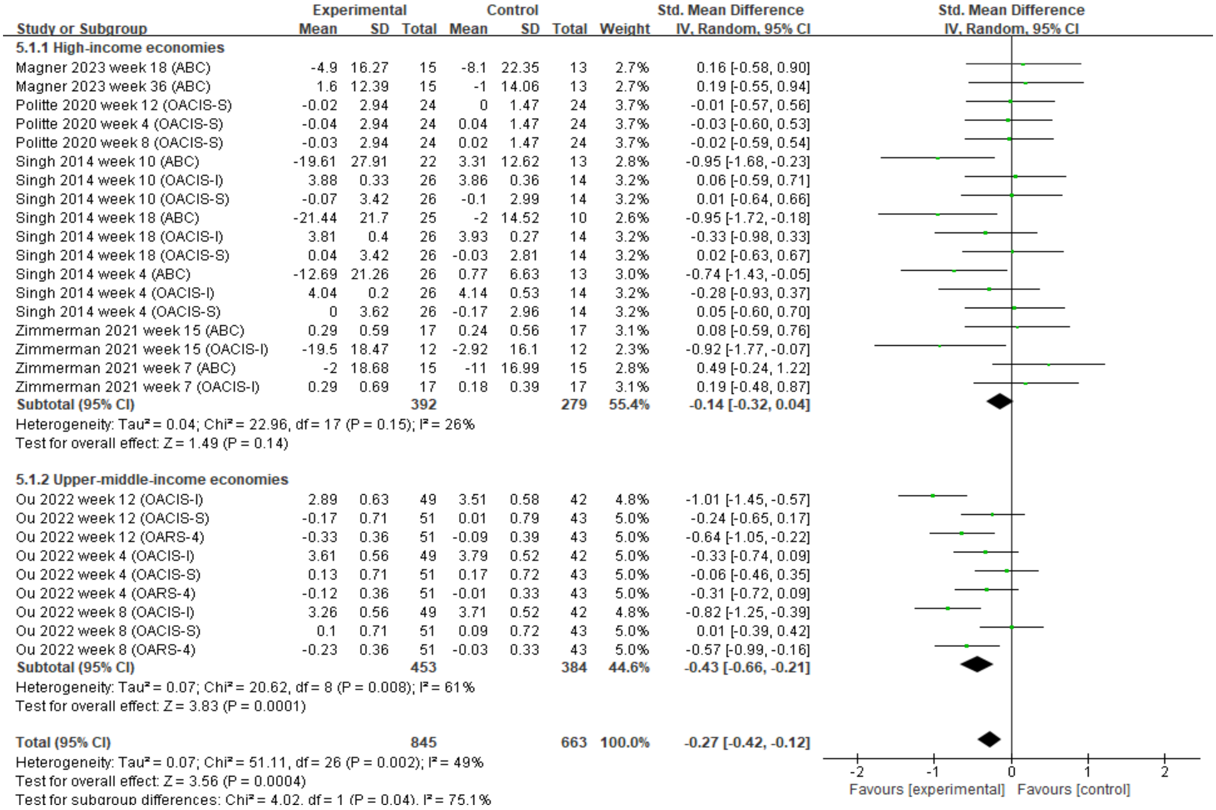

Supplementary Figure 4.1.1: Subgroup analyses of different national income levels on total symptoms

EXCLI Journal 2025;24:542-557 – ISSN 1611-2156
Received: January 31, 2025, accepted: April 02, 2025, published: April 07, 2025

Study or Subgroup	Experimental Mean	Experimental SD	Experimental Total	Control Mean	Control SD	Control Total	Weight	Std. Mean Difference IV, Random, 95% CI
**5.6.1 High-income economies**								
Magner 2023 week 18 (ADOS)	-0.2	1.94	15	0.08	2.16	13	2.2%	-0.13 [-0.88, 0.61]
Magner 2023 week 36 (ADOS)	0.5	1.94	15	0.7	1.44	13	2.2%	-0.11 [-0.86, 0.63]
Politte 2020 week 12 (ABC)	0	15.68	24	0	9.8	24	2.9%	0.00 [-0.57, 0.57]
Politte 2020 week 12 (RBS-R)	0.3	68.59	24	-0.3	42.62	24	2.9%	0.01 [-0.56, 0.58]
Politte 2020 week 12 (SRS)	0.1	22.05	24	-0.1	13.72	24	2.9%	0.01 [-0.56, 0.58]
Politte 2020 week 4 (ABC)	-0.2	15.68	24	0.2	9.8	24	2.9%	-0.03 [-0.60, 0.54]
Politte 2020 week 4 (RBS-R)	-1.3	68.59	24	0.2	42.62	24	2.9%	-0.03 [-0.59, 0.54]
Politte 2020 week 4 (SRS)	-0.5	22.05	24	0.6	13.72	24	2.9%	-0.06 [-0.62, 0.51]
Politte 2020 week 8 (ABC)	-0.3	15.68	24	0.3	9.8	24	2.9%	-0.05 [-0.61, 0.52]
Politte 2020 week 8 (RBS-R)	0.3	68.59	24	-0.3	42.62	24	2.9%	0.01 [-0.56, 0.58]
Politte 2020 week 8 (SRS)	-0.9	22.05	24	0.9	13.72	24	2.9%	-0.10 [-0.66, 0.47]
Singh 2014 week 10 (OACIS-I) I	3.46	0.71	26	3.79	0.43	14	2.5%	-0.51 [-1.18, 0.15]
Singh 2014 week 10 (OACIS-S) B	-0.52	5.86	26	0.01	3.18	14	2.5%	-0.10 [-0.75, 0.55]
Singh 2014 week 10 (OACIS-S) I	-0.49	4.39	26	-0.33	3.26	14	2.5%	-0.04 [-0.69, 0.61]
Singh 2014 week 18 (OACIS-I) B	3.42	0.86	26	4	0.39	14	2.5%	-0.77 [-1.45, -0.10]
Singh 2014 week 18 (OACIS-S) B	-0.45	5.3	26	0.01	2.99	14	2.5%	-0.10 [-0.75, 0.55]
Singh 2014 week 18 (OACIS-S) I	-0.49	4.84	26	-0.12	3.07	14	2.5%	-0.08 [-0.73, 0.57]
Singh 2014 week 4 (OACIS-I) B	3.81	0.57	26	3.71	0.61	14	2.5%	0.17 [-0.48, 0.82]
Singh 2014 week 4 (OACIS-I) I	3.81	0.49	26	3.86	0.53	14	2.5%	-0.10 [-0.75, 0.55]
Singh 2014 week 4 (OACIS-S) B	-0.06	5.56	26	-0.27	3.7	14	2.5%	0.04 [-0.61, 0.69]
Singh 2014 week 4 (OACIS-S) I	-0.14	4.28	26	-0.26	2.73	14	2.5%	0.03 [-0.62, 0.68]
Zimmerman 2021 week 15 (ABC)	-0.15	0.63	12	-0.13	0.39	12	2.1%	-0.04 [-0.84, 0.76]
Zimmerman 2021 week 15 (OACIS-I) B	0.06	0.43	17	0.12	0.49	17	2.5%	-0.13 [-0.80, 0.55]
Zimmerman 2021 week 15 (OACIS-I) I	0.12	0.49	17	0.24	0.56	17	2.4%	-0.22 [-0.90, 0.45]
Zimmerman 2021 week 15 (SRS)	-4.25	6.63	12	-0.75	4.79	12	2.0%	-0.58 [-1.40, 0.24]
Zimmerman 2021 week 7 (ABC)	-0.3	1.07	15	-0.27	0.62	15	2.3%	-0.03 [-0.75, 0.68]
Zimmerman 2021 week 7 (OACIS-I) B	0.12	0.33	17	0.12	0.33	17	2.5%	0.00 [-0.67, 0.67]
Zimmerman 2021 week 7 (OACIS-I) I	0.06	0.24	17	0.06	0.24	17	2.5%	0.00 [-0.67, 0.67]
Zimmerman 2021 week 7 (SRS)	-0.33	6.1	15	-2.67	5.17	15	2.3%	0.40 [-0.32, 1.13]
**Subtotal (95% CI)**			**628**			**504**	**73.8%**	**-0.08 [-0.20, 0.04]**
Heterogeneity: $Tau^2 = 0.00$; $Chi^2 = 10.54$, df = 28 (P = 1.00); $I^2 = 0\%$								
Test for overall effect: Z = 1.27 (P = 0.20)								
**5.6.2 Upper-middle-income economies**								
Momtazmanesh 2020 week 10 (ABC)	-2.87	0.6	30	-1.97	0.45	30	2.7%	-1.68 [-2.27, -1.08]
Momtazmanesh 2020 week 5 (ABC)	-2.13	0.45	30	-1.23	0.31	30	2.5%	-2.30 [-2.96, -1.64]
Ou 2022 week 12 (OARS-4)	-0.04	1.57	51	0.37	1.51	43	3.5%	-0.26 [-0.67, 0.14]
Ou 2022 week 12 (RBS-R)	-3.54	14.27	48	-9.82	14.22	41	3.5%	0.44 [0.02, 0.86]
Ou 2022 week 4 (OARS-4)	-0.14	1.14	51	-0.06	0.39	43	3.5%	-0.09 [-0.50, 0.32]
Ou 2022 week 4 (RBS-R)	-3.23	10.88	48	-6.22	10.76	41	3.5%	0.27 [-0.15, 0.69]
Ou 2022 week 8 (OARS-4)	0.07	1.14	51	0.19	1.05	43	3.5%	-0.11 [-0.51, 0.30]
Ou 2022 week 8 (RBS-R)	-4.65	13.37	48	-4.29	13.58	41	3.5%	-0.03 [-0.44, 0.39]
**Subtotal (95% CI)**			**357**			**312**	**26.2%**	**-0.43 [-0.96, 0.10]**
Heterogeneity: $Tau^2 = 0.52$; $Chi^2 = 77.28$, df = 7 (P < 0.00001); $I^2 = 91\%$								
Test for overall effect: Z = 1.60 (P = 0.11)								
**Total (95% CI)**			**985**			**816**	**100.0%**	**-0.16 [-0.31, -0.00]**
Heterogeneity: $Tau^2 = 0.13$; $Chi^2 = 89.89$, df = 36 (P < 0.00001); $I^2 = 60\%$								
Test for overall effect: Z = 2.01 (P = 0.04)								
Test for subgroup differences: $Chi^2 = 1.65$, df = 1 (P = 0.20), $I^2 = 39.4\%$								

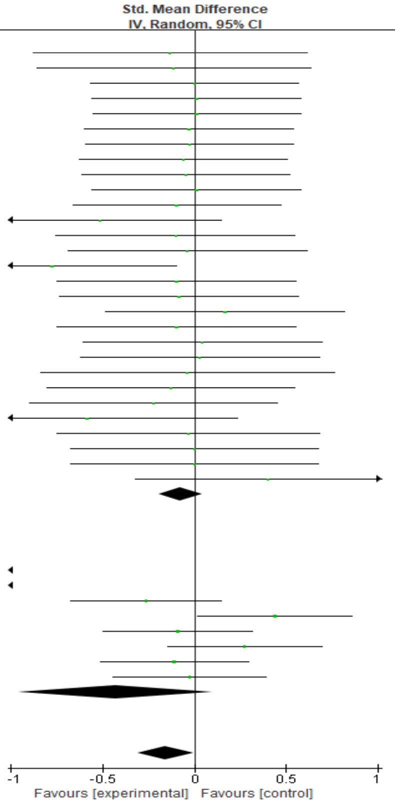

Supplementary Figure 4.1.2: Subgroup analyses of different national income levels on restricted interests and repetitive behavior

Received: January 31, 2025, accepted: April 02, 2025, published: April 07, 2025

Supplementary Figure 4.2: Subgroup analyses of different ages

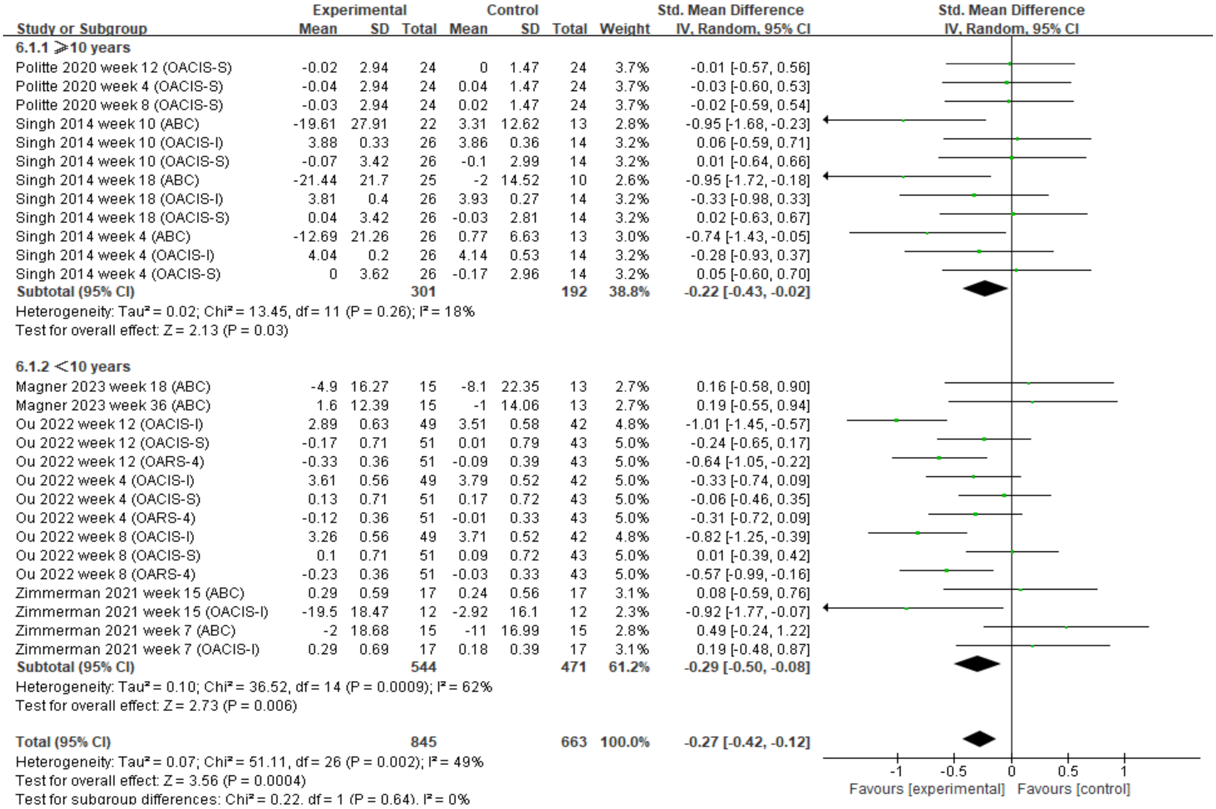

Supplementary Figure 4.2.1: Subgroup analyses of different ages on total symptoms

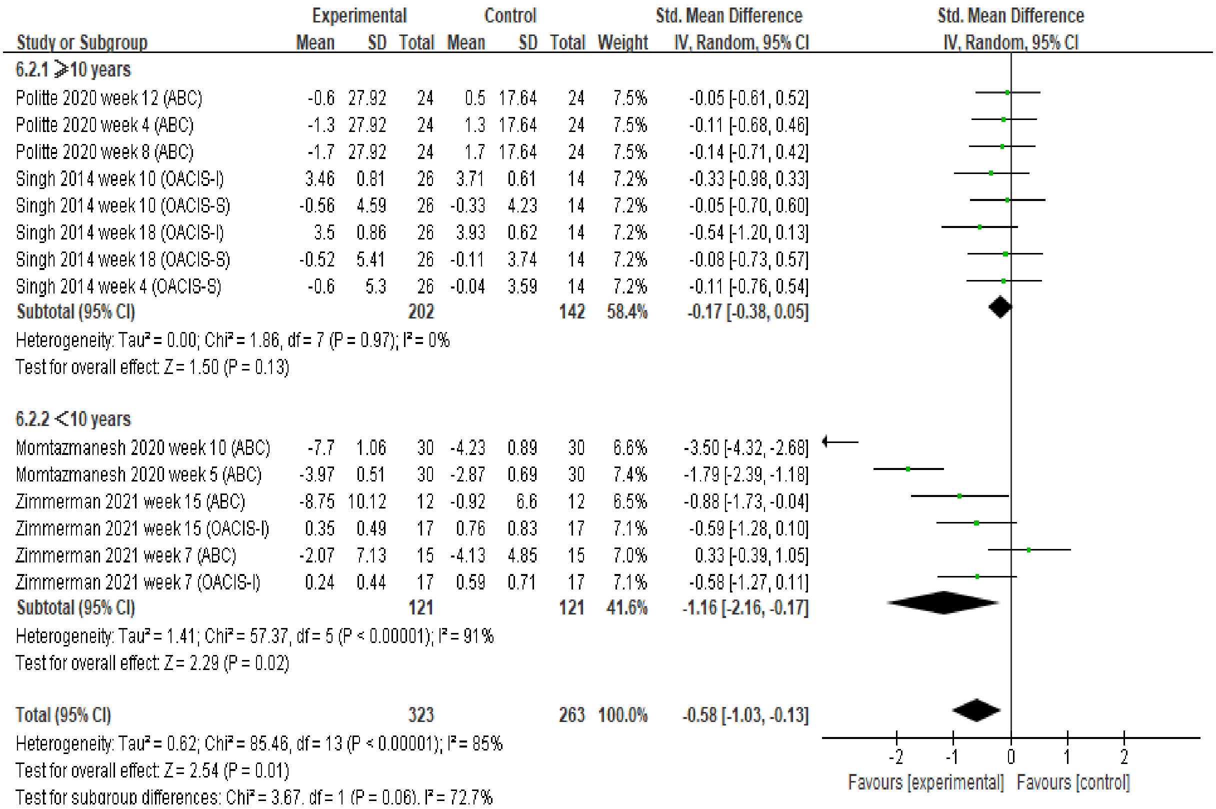

Supplementary Figure 4.2.2: Subgroup analyses of different ages on hyperactivity

EXCLI Journal 2025;24:542-557 – ISSN 1611-2156
Received: January 31, 2025, accepted: April 02, 2025, published: April 07, 2025

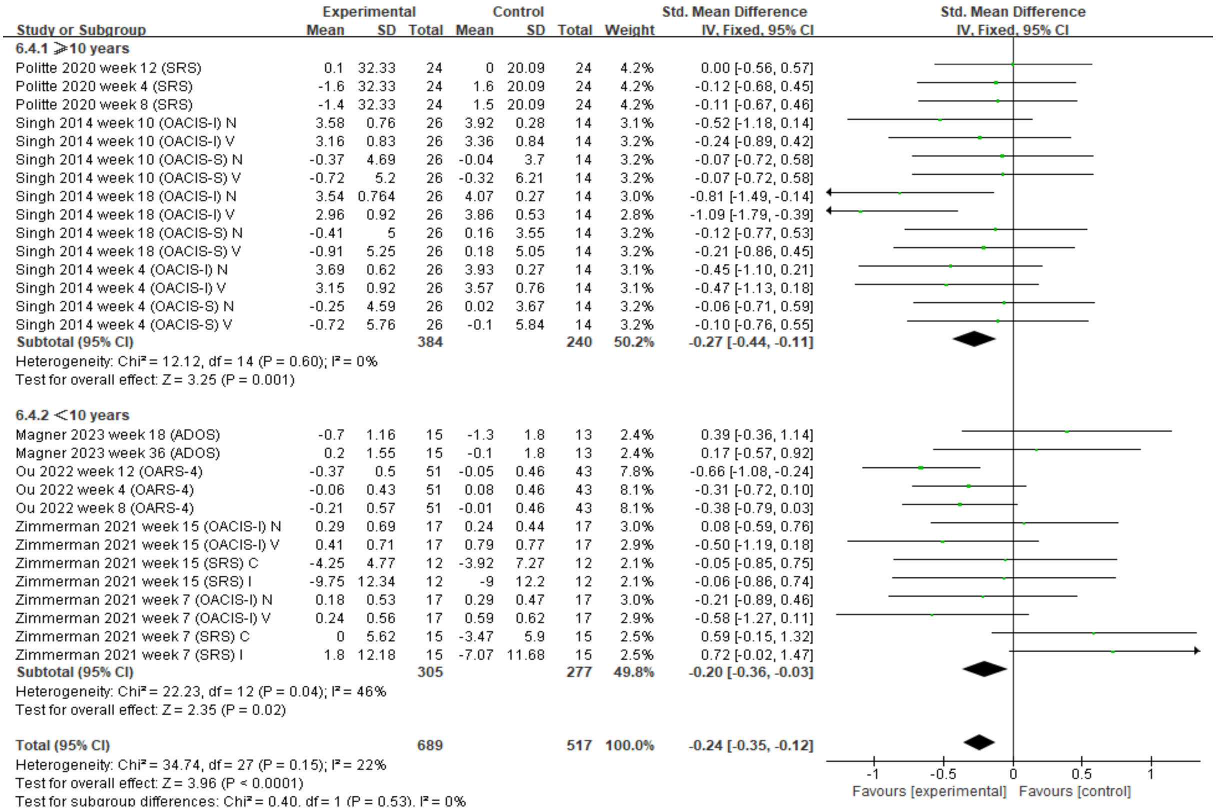

Supplementary Figure 4.2.3: Subgroup analyses of different ages on social communication

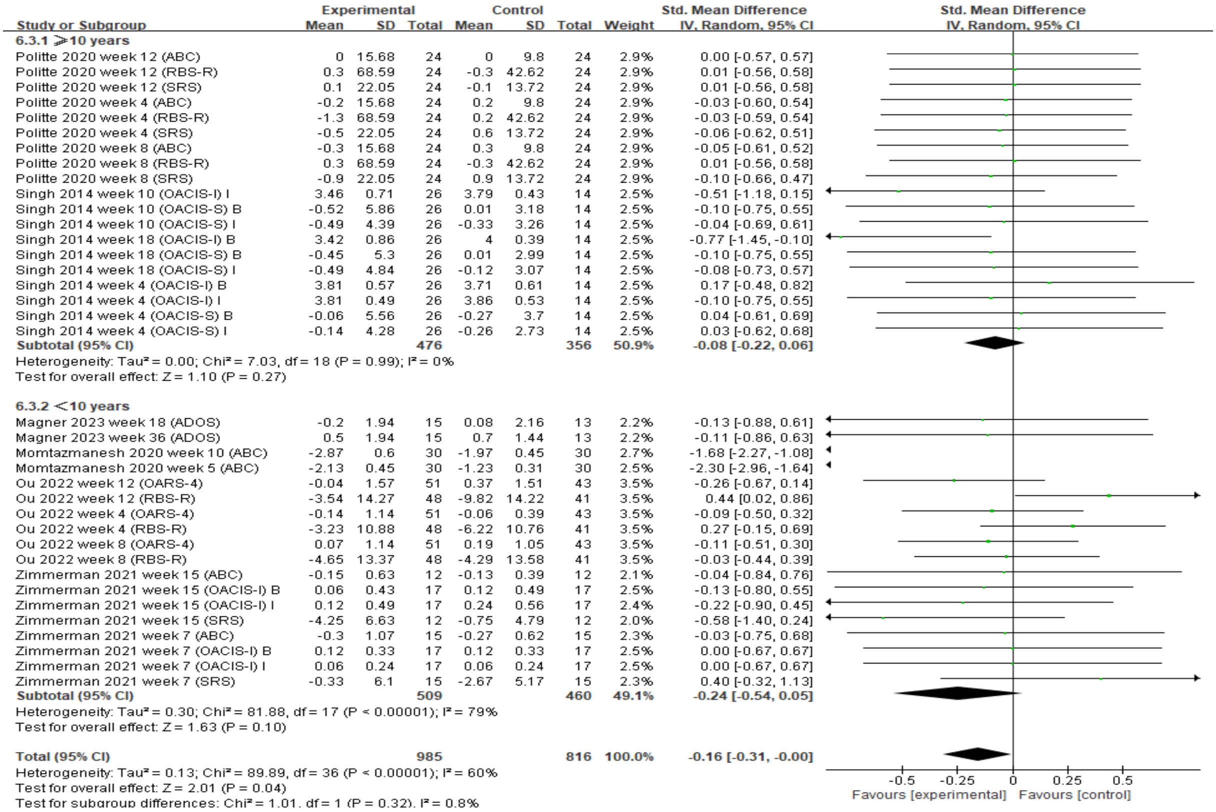

Supplementary Figure 4.2.4: Subgroup analyses of different ages on restricted interests and repetitive behavior

EXCLI Journal 2025;24:542-557 – ISSN 1611-2156
Received: January 31, 2025, accepted: April 02, 2025, published: April 07, 2025

	Experimental			Control				Std. Mean Difference
Study or Subgroup	Mean	SD	Total	Mean	SD	Total	Weight	IV, Fixed, 95% CI
**6.5.1 ≥10 years**								
Politte 2020 week 12 (SRS)	-0.3	36.25	24	0.3	22.54	24	5.4%	-0.02 [-0.59, 0.55]
Politte 2020 week 4 (SRS)	-1.5	36.25	24	1.5	22.54	24	5.4%	-0.10 [-0.66, 0.47]
Politte 2020 week 8 (SRS)	-1.9	36.25	24	1.9	22.54	24	5.4%	-0.12 [-0.69, 0.44]
Singh 2014 week 10 (SRS)	-14.48	28.6	23	-1.38	13.41	13	3.6%	-0.53 [-1.22, 0.17]
Singh 2014 week 18 (SRS)	-20.4	22.7	25	-2	11.48	11	3.1%	-0.90 [-1.64, -0.15]
Singh 2014 week 4 (SRS)	-14.76	18.95	25	-7.79	11.56	14	4.0%	-0.41 [-1.07, 0.25]
**Subtotal (95% CI)**			**145**			**110**	**26.9%**	**-0.28 [-0.54, -0.03]**
Heterogeneity: Chi² = 4.78, df = 5 (P = 0.44); I² = 0%								
Test for overall effect: Z = 2.19 (P = 0.03)								
**6.5.2 <10 years**								
Magner 2023 week 18 (SRS)	-2	12.01	15	-9.7	21.99	13	3.1%	0.43 [-0.32, 1.18]
Magner 2023 week 36 (SRS)	-0.1	12.01	15	-2.7	11.54	13	3.1%	0.21 [-0.53, 0.96]
Ou 2022 week 12 (AUBC)	-0.87	6	51	-1.3	5.88	40	10.1%	0.07 [-0.34, 0.49]
Ou 2022 week 12 (SRS)	-1.83	22.42	51	-3.88	22.67	41	10.2%	0.09 [-0.32, 0.50]
Ou 2022 week 4 (AUBC)	-1.01	4.57	51	-1.1	4.55	40	10.1%	0.02 [-0.39, 0.43]
Ou 2022 week 4 (SRS)	-2.98	20	51	-0.79	19.91	41	10.2%	-0.11 [-0.52, 0.30]
Ou 2022 week 8 (AUBC)	-0.57	5.71	51	-1.15	5.63	40	10.1%	0.10 [-0.31, 0.52]
Ou 2022 week 8 (SRS)	-1.92	21.85	51	-0.4	21.9	41	10.2%	-0.07 [-0.48, 0.34]
Zimmerman 2021 week 15 (SRS)	-14	18.49	12	-9.75	16.22	12	2.7%	-0.24 [-1.04, 0.57]
Zimmerman 2021 week 7 (SRS)	1.47	17.06	15	-9.73	15.98	15	3.2%	0.66 [-0.08, 1.40]
**Subtotal (95% CI)**			**363**			**296**	**73.1%**	**0.06 [-0.09, 0.22]**
Heterogeneity: Chi² = 5.28, df = 9 (P = 0.81); I² = 0%								
Test for overall effect: Z = 0.78 (P = 0.43)								
**Total (95% CI)**			**508**			**406**	**100.0%**	**-0.03 [-0.16, 0.10]**
Heterogeneity: Chi² = 15.26, df = 15 (P = 0.43); I² = 2%								
Test for overall effect: Z = 0.47 (P = 0.64)								
Test for subgroup differences: Chi² = 5.20, df = 1 (P = 0.02), I² = 80.8%								

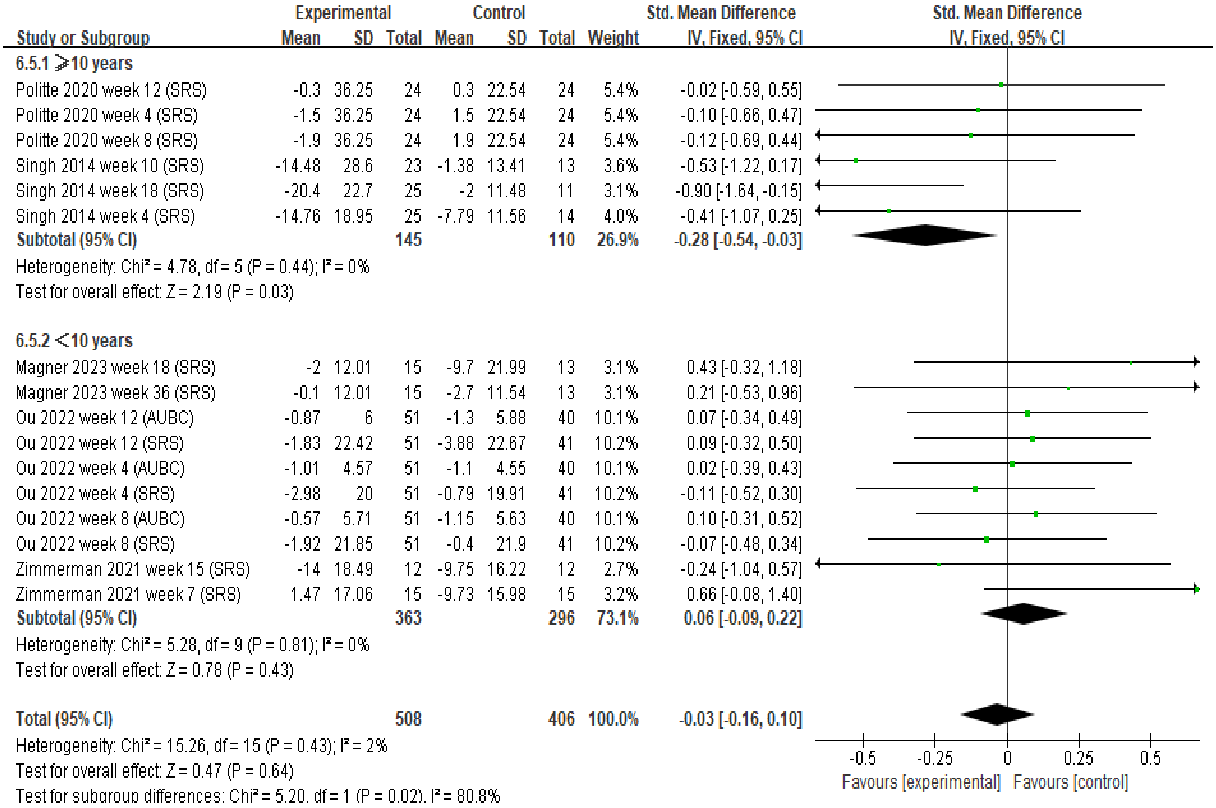

Supplementary Figure 4.2.5: Subgroup analyses of different ages on total social skills

EXCLI Journal 2025;24:542-557 – ISSN 1611-2156
Received: January 31, 2025, accepted: April 02, 2025, published: April 07, 2025

Supplementary Figure 4.3: Subgroup analyses of different measurement tools

Study or Subgroup	Experimental Mean	Experimental SD	Experimental Total	Control Mean	Control SD	Control Total	Weight	Mean Difference IV, Random, 95% CI
**3.1.1 ABC**								
Magner 2023 week 18 (ABC)	-4.9	16.27	15	-8.1	22.35	13	0.0%	3.20 [-11.48, 17.88]
Magner 2023 week 36 (ABC)	1.6	12.39	15	-1	14.06	13	0.0%	2.60 [-7.29, 12.49]
Singh 2014 week 10 (ABC)	-19.61	27.91	22	3.31	12.62	13	0.0%	-22.92 [-36.45, -9.39]
Singh 2014 week 18 (ABC)	-21.44	21.7	25	-2	14.52	10	0.0%	-19.44 [-31.82, -7.06]
Singh 2014 week 4 (ABC)	-12.69	21.26	26	0.77	6.63	13	0.0%	-13.46 [-22.39, -4.53]
Zimmerman 2021 week 15 (ABC)	-19.5	18.47	12	-2.92	16.1	12	0.0%	-16.58 [-30.44, -2.72]
Zimmerman 2021 week 7 (ABC)	-2	18.68	15	-11	16.99	15	0.0%	9.00 [-3.78, 21.78]
**Subtotal (95% CI)**			**130**			**89**	**0.2%**	**-8.20 [-17.32, 0.93]**
Heterogeneity: Tau² = 112.31; Chi² = 24.31, df = 6 (P = 0.0005); I² = 75%								
Test for overall effect: Z = 1.76 (P = 0.08)								
**3.1.2 OACIS-I**								
Ou 2022 week 12 (OACIS-I)	2.89	0.63	49	3.51	0.58	42	8.9%	-0.62 [-0.87, -0.37]
Ou 2022 week 4 (OACIS-I)	3.61	0.56	49	3.79	0.52	42	9.2%	-0.18 [-0.40, 0.04]
Ou 2022 week 8 (OACIS-I)	3.26	0.56	49	3.71	0.52	42	9.2%	-0.45 [-0.67, -0.23]
Singh 2014 week 10 (OACIS-I)	3.88	0.33	26	3.86	0.36	14	9.1%	0.02 [-0.21, 0.25]
Singh 2014 week 18 (OACIS-I)	3.81	0.4	26	3.93	0.27	14	9.4%	-0.12 [-0.33, 0.09]
Singh 2014 week 4 (OACIS-I)	4.04	0.2	26	4.14	0.53	14	8.4%	-0.10 [-0.39, 0.19]
Zimmerman 2021 week 15 (OACIS-I)	0.29	0.59	17	0.24	0.56	17	7.1%	0.05 [-0.34, 0.44]
Zimmerman 2021 week 7 (OACIS-I)	0.29	0.69	17	0.18	0.39	17	7.2%	0.11 [-0.27, 0.49]
**Subtotal (95% CI)**			**259**			**202**	**68.4%**	**-0.18 [-0.35, -0.01]**
Heterogeneity: Tau² = 0.04; Chi² = 24.68, df = 7 (P = 0.0009); I² = 72%								
Test for overall effect: Z = 2.04 (P = 0.04)								
**3.1.3 OACIS-S**								
Ou 2022 week 12 (OACIS-S)	-0.17	0.71	51	0.01	0.79	43	8.1%	-0.18 [-0.49, 0.13]
Ou 2022 week 4 (OACIS-S)	0.13	0.71	51	0.17	0.72	43	8.3%	-0.04 [-0.33, 0.25]
Ou 2022 week 8 (OACIS-S)	0.1	0.71	51	0.09	0.72	43	8.3%	0.01 [-0.28, 0.30]
Politte 2020 week 12 (OACIS-S)	-0.02	2.94	24	0	1.47	24	1.5%	-0.02 [-1.34, 1.30]
Politte 2020 week 4 (OACIS-S)	-0.04	2.94	24	0.04	1.47	24	1.5%	-0.08 [-1.40, 1.24]
Politte 2020 week 8 (OACIS-S)	-0.03	2.94	24	0.02	1.47	24	1.5%	-0.05 [-1.37, 1.27]
Singh 2014 week 10 (OACIS-S)	-0.07	3.42	26	-0.1	2.99	14	0.7%	0.03 [-2.01, 2.07]
Singh 2014 week 18 (OACIS-S)	0.04	3.42	26	-0.03	2.81	14	0.7%	0.07 [-1.90, 2.04]
Singh 2014 week 4 (OACIS-S)	0	3.62	26	-0.17	2.96	14	0.7%	0.17 [-1.91, 2.25]
**Subtotal (95% CI)**			**303**			**243**	**31.4%**	**-0.06 [-0.23, 0.10]**
Heterogeneity: Tau² = 0.00; Chi² = 0.91, df = 8 (P = 1.00); I² = 0%								
Test for overall effect: Z = 0.74 (P = 0.46)								
**Total (95% CI)**			**692**			**534**	**100.0%**	**-0.15 [-0.32, 0.03]**
Heterogeneity: Tau² = 0.07; Chi² = 64.26, df = 23 (P < 0.00001); I² = 64%								
Test for overall effect: Z = 1.67 (P = 0.09)								
Test for subgroup differences: Chi² = 3.94, df = 2 (P = 0.14), I² = 49.2%								

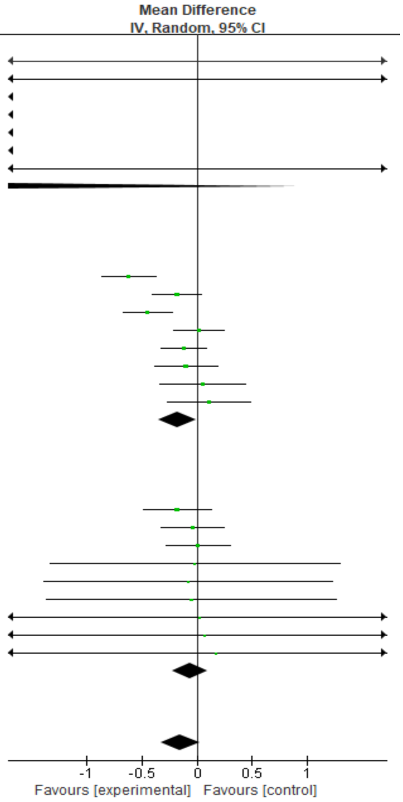

Supplementary Figure 4.3.1: Subgroup analyses of different measurement tools on total symptoms

Study or Subgroup	Experimental Mean	Experimental SD	Experimental Total	Control Mean	Control SD	Control Total	Weight	Mean Difference IV, Random, 95% CI
**3.2.1 ABC**								
Momtazmanesh 2020 week 10 (ABC)	-7.7	1.06	30	-4.23	0.89	30	15.5%	-3.47 [-3.97, -2.97]
Momtazmanesh 2020 week 5 (ABC)	-3.97	0.51	30	-2.87	0.69	30	16.1%	-1.10 [-1.41, -0.79]
Politte 2020 week 12 (ABC)	-0.6	27.92	24	0.5	17.64	24	0.4%	-1.10 [-14.31, 12.11]
Politte 2020 week 4 (ABC)	-1.3	27.92	24	1.3	17.64	24	0.4%	-2.60 [-15.81, 10.61]
Politte 2020 week 8 (ABC)	-1.7	27.92	24	1.7	17.64	24	0.4%	-3.40 [-16.61, 9.81]
Zimmerman 2021 week 15 (ABC)	-8.75	10.12	12	-0.92	6.6	12	1.4%	-7.83 [-14.67, -0.99]
Zimmerman 2021 week 7 (ABC)	-2.07	7.13	15	-4.13	4.85	15	3.0%	2.06 [-2.30, 6.42]
**Subtotal (95% CI)**			**159**			**159**	**37.2%**	**-2.09 [-4.07, -0.10]**
Heterogeneity: Tau² = 2.74; Chi² = 69.60, df = 6 (P < 0.00001); I² = 91%								
Test for overall effect: Z = 2.06 (P = 0.04)								
**3.2.2 OACIS-I**								
Singh 2014 week 10 (OACIS-I)	3.46	0.81	26	3.71	0.61	14	15.7%	-0.25 [-0.70, 0.20]
Singh 2014 week 18 (OACIS-I)	3.5	0.86	26	3.93	0.62	14	15.6%	-0.43 [-0.89, 0.03]
Zimmerman 2021 week 15 (OACIS-I)	0.35	0.49	17	0.76	0.83	17	15.6%	-0.41 [-0.87, 0.05]
Zimmerman 2021 week 7 (OACIS-I)	0.24	0.44	17	0.59	0.71	17	15.8%	-0.35 [-0.75, 0.05]
**Subtotal (95% CI)**			**86**			**62**	**62.8%**	**-0.36 [-0.58, -0.14]**
Heterogeneity: Tau² = 0.00; Chi² = 0.37, df = 3 (P = 0.95); I² = 0%								
Test for overall effect: Z = 3.19 (P = 0.001)								
**Total (95% CI)**			**245**			**221**	**100.0%**	**-1.02 [-1.86, -0.17]**
Heterogeneity: Tau² = 1.13; Chi² = 134.66, df = 10 (P < 0.00001); I² = 93%								
Test for overall effect: Z = 2.36 (P = 0.02)								
Test for subgroup differences: Chi² = 2.89, df = 1 (P = 0.09), I² = 65.4%								

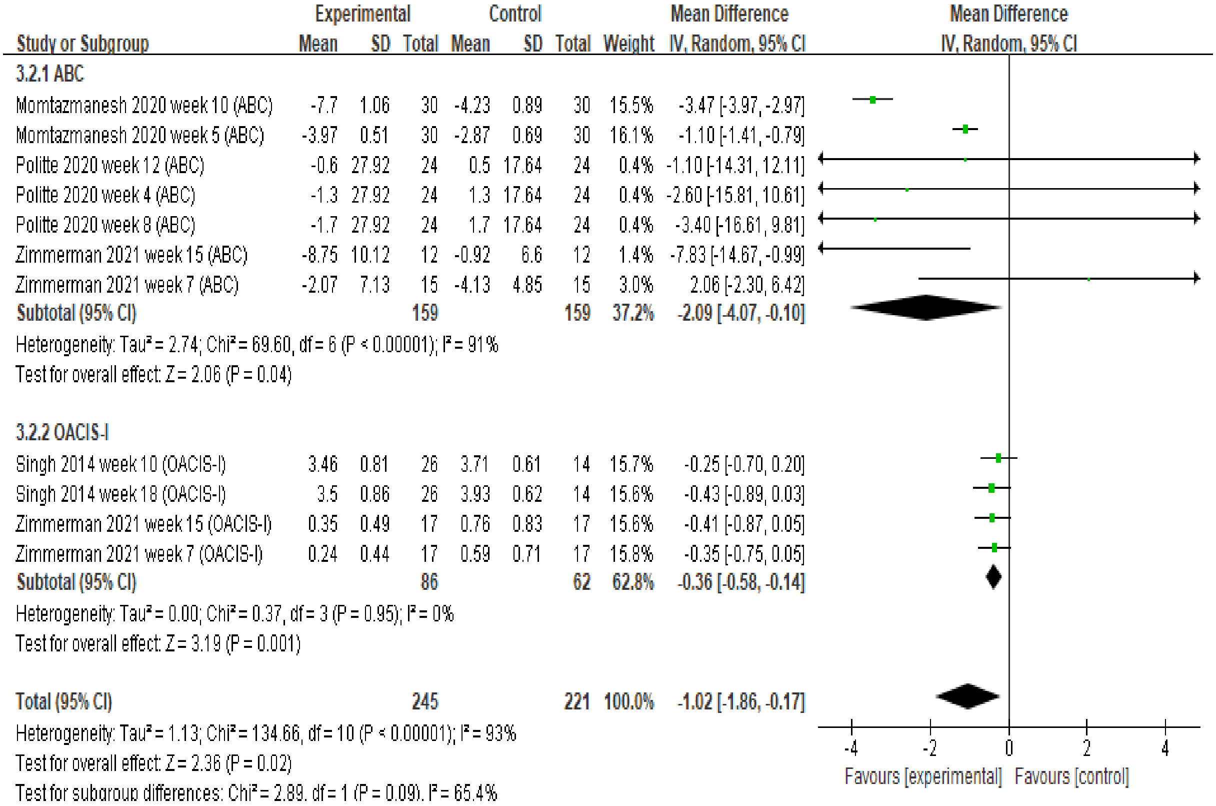

Supplementary Figure 4.3.2: Subgroup analyses of different measurement tools on hyperactivity

EXCLI Journal 2025;24:542-557 – ISSN 1611-2156
Received: January 31, 2025, accepted: April 02, 2025, published: April 07, 2025

Study or Subgroup	Experimental Mean	Experimental SD	Experimental Total	Control Mean	Control SD	Control Total	Weight	Mean Difference IV, Fixed, 95% CI
**3.5.1 SRS**								
Politte 2020 week 12 (SRS)	0.1	32.33	24	0	20.09	24	0.0%	0.10 [-15.13, 15.33]
Politte 2020 week 4 (SRS)	-1.6	32.33	24	1.6	20.09	24	0.0%	-3.20 [-18.43, 12.03]
Politte 2020 week 8 (SRS)	-1.4	32.33	24	1.5	20.09	24	0.0%	-2.90 [-18.13, 12.33]
Zimmerman 2021 week 15 (SRS) C	-4.25	4.77	12	-3.92	7.27	12	0.1%	-0.33 [-5.25, 4.59]
Zimmerman 2021 week 15 (SRS) I	-9.75	12.34	12	-9	12.2	12	0.0%	-0.75 [-10.57, 9.07]
Zimmerman 2021 week 7 (SRS) C	0	5.62	15	-3.47	5.9	15	0.1%	3.47 [-0.65, 7.59]
Zimmerman 2021 week 7 (SRS) I	1.8	12.18	15	-7.07	11.68	15	0.0%	8.87 [0.33, 17.41]
**Subtotal (95% CI)**			**126**			**126**	**0.2%**	**2.03 [-0.67, 4.73]**
Heterogeneity: Chi² = 5.04, df = 6 (P = 0.54); I² = 0%								
Test for overall effect: Z = 1.47 (P = 0.14)								
**3.5.2 OACIS-I**								
Singh 2014 week 10 (OACIS-I) N	3.58	0.76	26	3.92	0.28	14	13.4%	-0.34 [-0.67, -0.01]
Singh 2014 week 10 (OACIS-I) V	3.16	0.83	26	3.36	0.84	14	4.9%	-0.20 [-0.74, 0.34]
Singh 2014 week 18 (OACIS-I) N	3.54	0.764	26	4.07	0.27	14	13.5%	-0.53 [-0.86, -0.20]
Singh 2014 week 18 (OACIS-I) V	2.96	0.92	26	3.86	0.53	14	7.1%	-0.90 [-1.35, -0.45]
Singh 2014 week 4 (OACIS-I) N	3.69	0.62	26	3.93	0.27	14	18.7%	-0.24 [-0.52, 0.04]
Singh 2014 week 4 (OACIS-I) V	3.15	0.92	26	3.57	0.76	14	5.1%	-0.42 [-0.95, 0.11]
Zimmerman 2021 week 15 (OACIS-I) N	0.29	0.69	17	0.24	0.44	17	9.5%	0.05 [-0.34, 0.44]
Zimmerman 2021 week 15 (OACIS-I) V	0.41	0.71	17	0.79	0.77	17	5.8%	-0.38 [-0.88, 0.12]
Zimmerman 2021 week 7 (OACIS-I) N	0.18	0.53	17	0.29	0.47	17	12.7%	-0.11 [-0.45, 0.23]
Zimmerman 2021 week 7 (OACIS-I) V	0.24	0.56	17	0.59	0.62	17	9.1%	-0.35 [-0.75, 0.05]
**Subtotal (95% CI)**			**224**			**152**	**99.8%**	**-0.32 [-0.44, -0.20]**
Heterogeneity: Chi² = 13.69, df = 9 (P = 0.13); I² = 34%								
Test for overall effect: Z = 5.24 (P < 0.00001)								
**Total (95% CI)**			**350**			**278**	**100.0%**	**-0.32 [-0.44, -0.20]**
Heterogeneity: Chi² = 21.64, df = 16 (P = 0.16); I² = 26%								
Test for overall effect: Z = 5.17 (P < 0.00001)								
Test for subgroup differences: Chi² = 2.91, df = 1 (P = 0.09), I² = 65.6%								

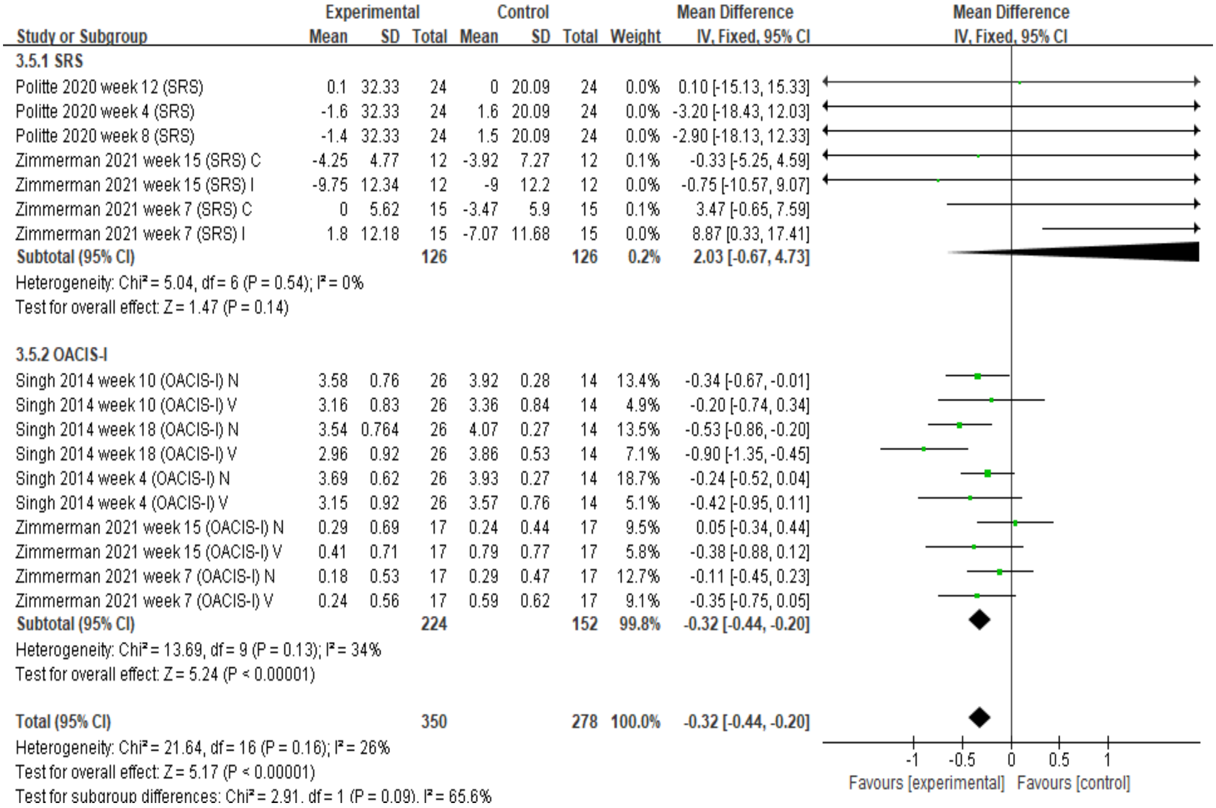

Supplementary Figure 4.3.3: Subgroup analyses of different measurement tools on social communication

Study or Subgroup	Experimental Mean	Experimental SD	Experimental Total	Control Mean	Control SD	Control Total	Weight	Mean Difference IV, Random, 95% CI
**3.4.1 ABC**								
Momtazmanesh 2020 week 10 (ABC)	-2.87	0.6	30	-1.97	0.45	30	8.8%	-0.90 [-1.17, -0.63]
Momtazmanesh 2020 week 5 (ABC)	-2.13	0.45	30	-1.23	0.31	30	9.3%	-0.90 [-1.10, -0.70]
Politte 2020 week 12 (ABC)	0	15.68	24	0	9.8	24	0.1%	0.00 [-7.40, 7.40]
Politte 2020 week 4 (ABC)	-0.2	15.68	24	0.2	9.8	24	0.1%	-0.40 [-7.80, 7.00]
Politte 2020 week 8 (ABC)	-0.3	15.68	24	0.3	9.8	24	0.1%	-0.60 [-8.00, 6.80]
Zimmerman 2021 week 15 (ABC)	-0.15	0.63	12	-0.13	0.39	12	7.5%	-0.02 [-0.44, 0.40]
Zimmerman 2021 week 7 (ABC)	-0.3	1.07	15	-0.27	0.62	15	5.8%	-0.03 [-0.66, 0.60]
**Subtotal (95% CI)**			**159**			**159**	**31.7%**	**-0.53 [-0.93, -0.14]**
Heterogeneity: Tau² = 0.13; Chi² = 20.24, df = 6 (P = 0.003); I² = 70%								
Test for overall effect: Z = 2.65 (P = 0.008)								
**3.4.2 RBS-R**								
Ou 2022 week 12 (RBS-R)	-3.54	14.27	48	-9.82	14.22	41	0.2%	6.28 [0.34, 12.22]
Ou 2022 week 4 (RBS-R)	-3.23	10.88	48	-6.22	10.76	41	0.3%	2.99 [-1.52, 7.50]
Ou 2022 week 8 (RBS-R)	-4.65	13.37	48	-4.29	13.58	41	0.2%	-0.36 [-5.98, 5.26]
Politte 2020 week 12 (RBS-R)	0.3	68.59	24	-0.3	42.62	24	0.0%	0.60 [-31.71, 32.91]
Politte 2020 week 4 (RBS-R)	-1.3	68.59	24	0.2	42.62	24	0.0%	-1.50 [-33.81, 30.81]
Politte 2020 week 8 (RBS-R)	0.3	68.59	24	-0.3	42.62	24	0.0%	0.60 [-31.71, 32.91]
**Subtotal (95% CI)**			**216**			**195**	**0.6%**	**2.80 [-0.19, 5.78]**
Heterogeneity: Tau² = 0.00; Chi² = 2.65, df = 5 (P = 0.75); I² = 0%								
Test for overall effect: Z = 1.84 (P = 0.07)								
**3.4.3 SRS**								
Politte 2020 week 12 (SRS)	0.1	22.05	24	-0.1	13.72	24	0.1%	0.20 [-10.19, 10.59]
Politte 2020 week 4 (SRS)	-0.5	22.05	24	0.6	13.72	24	0.1%	-1.10 [-11.49, 9.29]
Politte 2020 week 8 (SRS)	-0.9	22.05	24	0.9	13.72	24	0.1%	-1.80 [-12.19, 8.59]
Zimmerman 2021 week 15 (SRS)	-4.25	6.63	12	-0.75	4.79	12	0.2%	-3.50 [-8.13, 1.13]
Zimmerman 2021 week 7 (SRS)	-0.33	6.1	15	-2.67	5.17	15	0.3%	2.34 [-1.71, 6.39]
**Subtotal (95% CI)**			**99**			**99**	**0.7%**	**-0.34 [-3.05, 2.38]**
Heterogeneity: Tau² = 0.00; Chi² = 3.58, df = 4 (P = 0.47); I² = 0%								
Test for overall effect: Z = 0.24 (P = 0.81)								
**3.4.4 OACIS-I**								
Singh 2014 week 10 (OACIS-I) I	3.46	0.71	26	3.79	0.43	14	8.1%	-0.33 [-0.68, 0.02]
Singh 2014 week 18 (OACIS-I) B	3.42	0.86	26	4	0.39	14	7.8%	-0.58 [-0.97, -0.19]
Singh 2014 week 4 (OACIS-I) B	3.81	0.57	26	3.71	0.61	14	7.8%	0.10 [-0.29, 0.49]
Singh 2014 week 4 (OACIS-I) I	3.81	0.49	26	3.86	0.53	14	8.2%	-0.05 [-0.39, 0.29]
Zimmerman 2021 week 15 (OACIS-I) B	0.06	0.43	17	0.12	0.49	17	8.4%	-0.06 [-0.37, 0.25]
Zimmerman 2021 week 15 (OACIS-I) I	0.12	0.49	17	0.24	0.56	17	8.1%	-0.12 [-0.47, 0.23]
Zimmerman 2021 week 7 (OACIS-I) B	0.12	0.33	17	0.12	0.33	17	9.1%	0.00 [-0.22, 0.22]
Zimmerman 2021 week 7 (OACIS-I) I	0.06	0.24	17	0.06	0.24	17	9.5%	0.00 [-0.16, 0.16]
**Subtotal (95% CI)**			**172**			**124**	**67.0%**	**-0.10 [-0.22, 0.03]**
Heterogeneity: Tau² = 0.01; Chi² = 10.63, df = 7 (P = 0.16); I² = 34%								
Test for overall effect: Z = 1.48 (P = 0.14)								
**Total (95% CI)**			**646**			**577**	**100.0%**	**-0.24 [-0.47, -0.00]**
Heterogeneity: Tau² = 0.14; Chi² = 99.81, df = 25 (P < 0.00001); I² = 75%								
Test for overall effect: Z = 1.97 (P = 0.05)								
Test for subgroup differences: Chi² = 7.98, df = 3 (P = 0.05), I² = 62.4%								

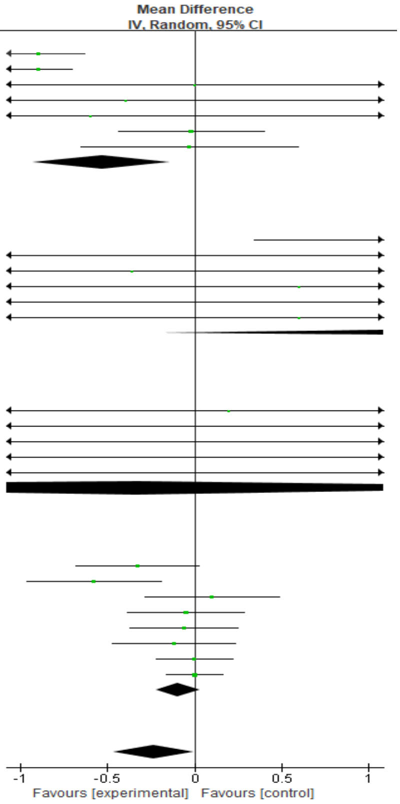

Supplementary Figure 4.3.4: Subgroup analyses of different measurement tools on restricted interests and repetitive behavior

EXCLI Journal 2025;24:542-557 – ISSN 1611-2156
Received: January 31, 2025, accepted: April 02, 2025, published: April 07, 2025

Study or Subgroup	Experimental Mean	Experimental SD	Experimental Total	Control Mean	Control SD	Control Total	Weight	Mean Difference IV, Random, 95% CI
**3.3.1 ABC**								
Momtazmanesh 2020 week 10 (ABC)	-3.5	0.87	30	-3.4	0.63	30	24.5%	-0.10 [-0.48, 0.28]
Momtazmanesh 2020 week 5 (ABC)	-2.57	0.7	30	-1.77	0.43	30	25.7%	-0.80 [-1.09, -0.51]
Politte 2020 week 12 (ABC)	0.3	24.99	24	-0.2	15.68	24	0.2%	0.50 [-11.30, 12.30]
Politte 2020 week 4 (ABC)	-0.5	24.99	24	0.5	15.68	24	0.2%	-1.00 [-12.80, 10.80]
Politte 2020 week 8 (ABC)	-0.5	24.99	24	0.5	15.68	24	0.2%	-1.00 [-12.80, 10.80]
Zimmerman 2021 week 15 (ABC)	-0.19	0.57	12	-0.33	1.06	12	19.8%	0.14 [-0.54, 0.82]
Zimmerman 2021 week 7 (ABC)	0.3	0.73	15	-0.38	0.89	15	21.4%	0.68 [0.10, 1.26]
**Subtotal (95% CI)**			**159**			**159**	**92.1%**	**-0.06 [-0.67, 0.54]**
Heterogeneity: $Tau^2 = 0.32$; $Chi^2 = 24.61$, df = 6 (P = 0.0004); $I^2 = 76\%$								
Test for overall effect: Z = 0.21 (P = 0.83)								
**3.3.2 SRS**								
Politte 2020 week 12 (SRS)	0	20.09	24	0	12.25	24	0.4%	0.00 [-9.41, 9.41]
Politte 2020 week 4 (SRS)	-0.7	20.09	24	0.7	12.25	24	0.4%	-1.40 [-10.81, 8.01]
Politte 2020 week 8 (SRS)	-0.9	20.09	24	0.9	12.25	24	0.4%	-1.80 [-11.21, 7.61]
Zimmerman 2021 week 15 (SRS)	-0.83	3.66	12	-2.5	3.94	12	3.1%	1.67 [-1.37, 4.71]
Zimmerman 2021 week 7 (SRS)	0.73	3.59	15	-1.47	4.13	15	3.7%	2.20 [-0.57, 4.97]
**Subtotal (95% CI)**			**99**			**99**	**7.9%**	**1.58 [-0.33, 3.50]**
Heterogeneity: $Tau^2 = 0.00$; $Chi^2 = 1.18$, df = 4 (P = 0.88); $I^2 = 0\%$								
Test for overall effect: Z = 1.62 (P = 0.11)								
**Total (95% CI)**			**258**			**258**	**100.0%**	**0.06 [-0.51, 0.63]**
Heterogeneity: $Tau^2 = 0.31$; $Chi^2 = 29.57$, df = 11 (P = 0.002); $I^2 = 63\%$								
Test for overall effect: Z = 0.21 (P = 0.83)								
Test for subgroup differences: $Chi^2 = 2.58$, df = 1 (P = 0.11), $I^2 = 61.3\%$								

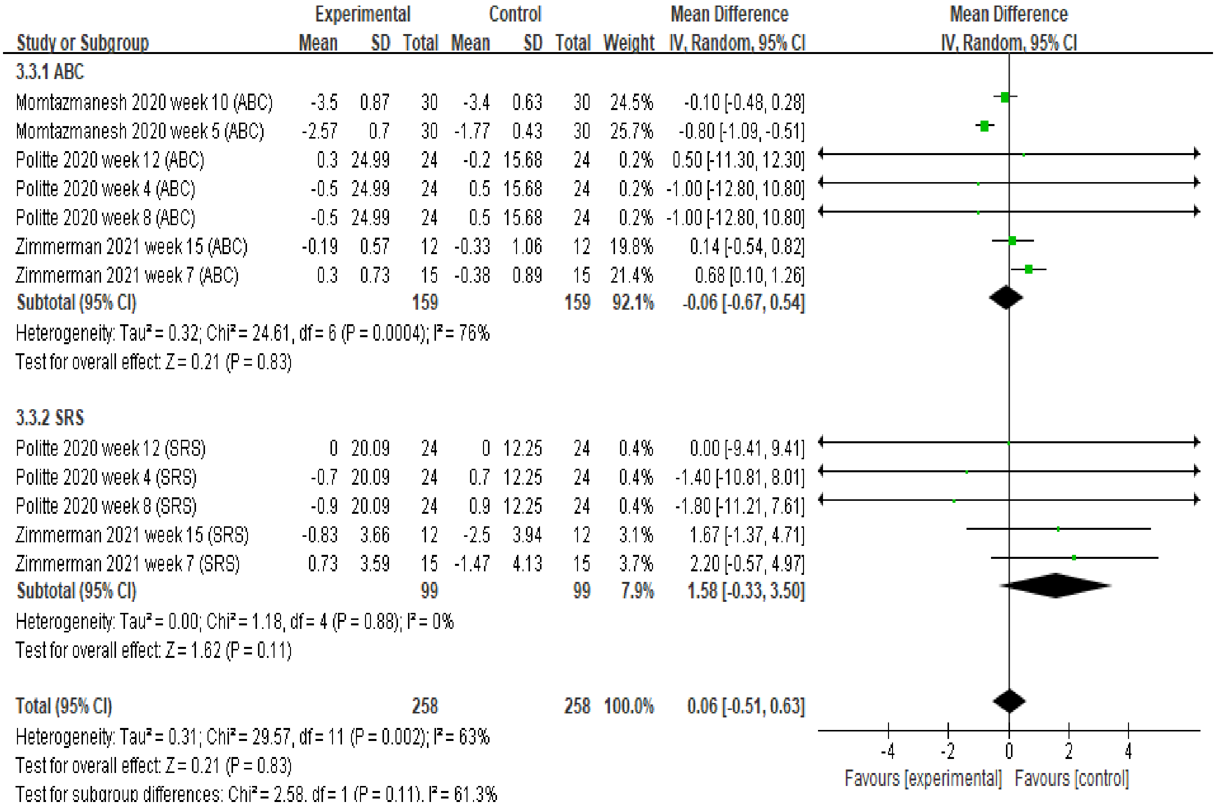

Supplementary Figure 4.3.5: Subgroup analyses of different measurement tools on social motivation

EXCLI Journal 2025;24:542-557 – ISSN 1611-2156
Received: January 31, 2025, accepted: April 02, 2025, published: April 07, 2025

Supplementary Figure 4.4: Subgroup analyses of different assessors of outcome indicators

Study or Subgroup	Experimental Mean	Experimental SD	Experimental Total	Control Mean	Control SD	Control Total	Weight	Std. Mean Difference IV, Random, 95% CI
**7.1.1 caregiver**								
Magner 2023 week 18 (ABC)	-4.9	16.27	15	-8.1	22.35	13	2.7%	0.16 [-0.58, 0.90]
Magner 2023 week 36 (ABC)	1.6	12.39	15	-1	14.06	13	2.7%	0.19 [-0.55, 0.94]
Singh 2014 week 10 (ABC)	-19.61	27.91	22	3.31	12.62	13	2.8%	-0.95 [-1.68, -0.23]
Singh 2014 week 18 (ABC)	-21.44	21.7	25	-2	14.52	10	2.6%	-0.95 [-1.72, -0.18]
Singh 2014 week 4 (ABC)	-12.69	21.26	26	0.77	6.63	13	3.0%	-0.74 [-1.43, -0.05]
Zimmerman 2021 week 15 (ABC)	0.29	0.59	17	0.24	0.56	17	3.1%	0.08 [-0.59, 0.76]
Zimmerman 2021 week 7 (ABC)	-2	18.68	15	-11	16.99	15	2.8%	0.49 [-0.24, 1.22]
**Subtotal (95% CI)**			**135**			**94**	**19.6%**	**-0.24 [-0.69, 0.21]**
Heterogeneity: Tau² = 0.23; Chi² = 16.14, df = 6 (P = 0.01); I² = 63%								
Test for overall effect: Z = 1.06 (P = 0.29)								
**7.1.2 professional**								
Ou 2022 week 12 (OACIS-I)	2.89	0.63	49	3.51	0.58	42	4.8%	-1.01 [-1.45, -0.57]
Ou 2022 week 12 (OACIS-S)	-0.17	0.71	51	0.01	0.79	43	5.0%	-0.24 [-0.65, 0.17]
Ou 2022 week 12 (OARS-4)	-0.33	0.36	51	-0.09	0.39	43	5.0%	-0.64 [-1.05, -0.22]
Ou 2022 week 4 (OACIS-I)	3.61	0.56	49	3.79	0.52	42	5.0%	-0.33 [-0.74, 0.09]
Ou 2022 week 4 (OACIS-S)	0.13	0.71	51	0.17	0.72	43	5.0%	-0.06 [-0.46, 0.35]
Ou 2022 week 4 (OARS-4)	-0.12	0.36	51	-0.01	0.33	43	5.0%	-0.31 [-0.72, 0.09]
Ou 2022 week 8 (OACIS-I)	3.26	0.56	49	3.71	0.52	42	4.8%	-0.82 [-1.25, -0.39]
Ou 2022 week 8 (OACIS-S)	0.1	0.71	51	0.09	0.72	43	5.0%	0.01 [-0.39, 0.42]
Ou 2022 week 8 (OARS-4)	-0.23	0.36	51	-0.03	0.33	43	5.0%	-0.57 [-0.99, -0.16]
Politte 2020 week 12 (OACIS-S)	-0.02	2.94	24	0	1.47	24	3.7%	-0.01 [-0.57, 0.56]
Politte 2020 week 4 (OACIS-S)	-0.04	2.94	24	0.04	1.47	24	3.7%	-0.03 [-0.60, 0.53]
Politte 2020 week 8 (OACIS-S)	-0.03	2.94	24	0.02	1.47	24	3.7%	-0.02 [-0.59, 0.54]
Singh 2014 week 10 (OACIS-I)	3.88	0.33	26	3.86	0.36	14	3.2%	0.06 [-0.59, 0.71]
Singh 2014 week 10 (OACIS-S)	-0.07	3.42	26	-0.1	2.99	14	3.2%	0.01 [-0.64, 0.66]
Singh 2014 week 18 (OACIS-I)	3.81	0.4	26	3.93	0.27	14	3.2%	-0.33 [-0.98, 0.33]
Singh 2014 week 18 (OACIS-S)	0.04	3.42	26	-0.03	2.81	14	3.2%	0.02 [-0.63, 0.67]
Singh 2014 week 4 (OACIS-I)	4.04	0.2	26	4.14	0.53	14	3.2%	-0.28 [-0.93, 0.37]
Singh 2014 week 4 (OACIS-S)	0	3.62	26	-0.17	2.96	14	3.2%	0.05 [-0.60, 0.70]
Zimmerman 2021 week 15 (OACIS-I)	-19.5	18.47	12	-2.92	16.1	12	2.3%	-0.92 [-1.77, -0.07]
Zimmerman 2021 week 7 (OACIS-I)	0.29	0.69	17	0.18	0.39	17	3.1%	0.19 [-0.48, 0.87]
**Subtotal (95% CI)**			**710**			**569**	**80.4%**	**-0.29 [-0.44, -0.13]**
Heterogeneity: Tau² = 0.06; Chi² = 34.77, df = 19 (P = 0.01); I² = 45%								
Test for overall effect: Z = 3.58 (P = 0.0003)								
**Total (95% CI)**			**845**			**663**	**100.0%**	**-0.27 [-0.42, -0.12]**
Heterogeneity: Tau² = 0.07; Chi² = 51.11, df = 26 (P = 0.002); I² = 49%								
Test for overall effect: Z = 3.56 (P = 0.0004)								
Test for subgroup differences: Chi² = 0.03, df = 1 (P = 0.86), I² = 0%								

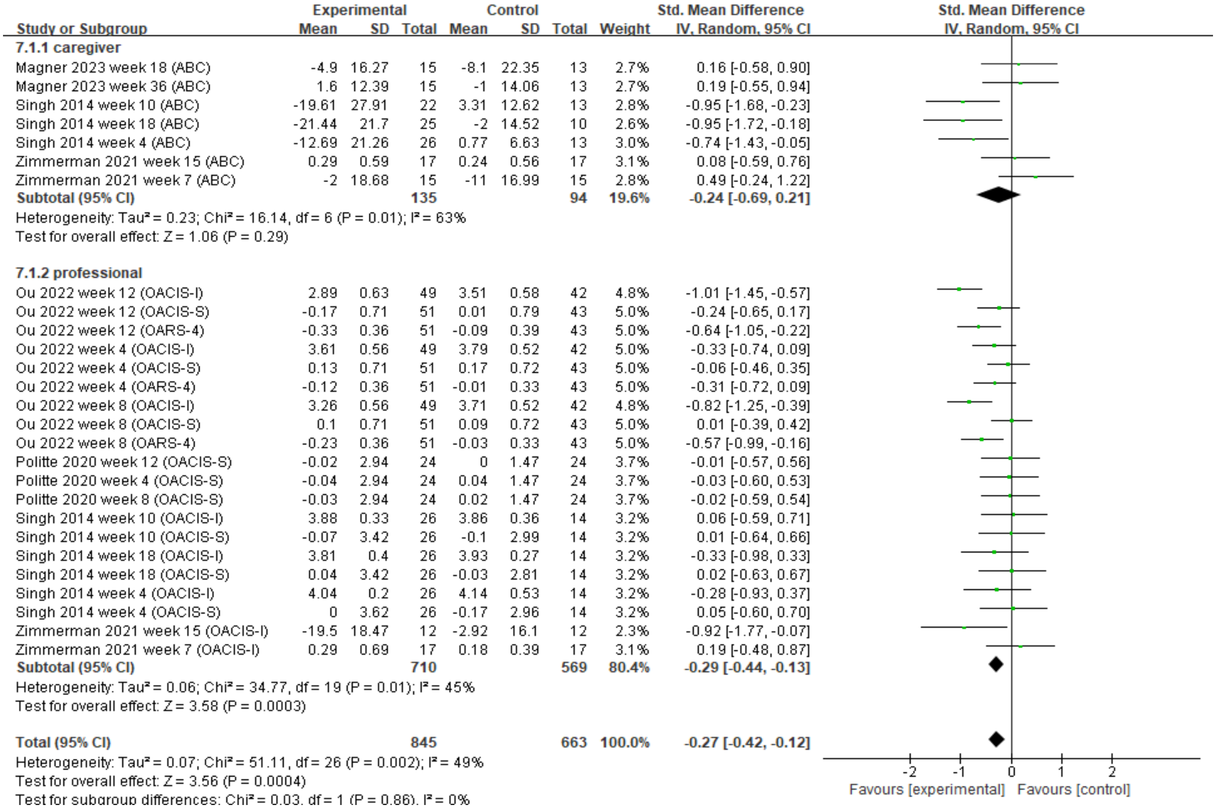

Supplementary Figure 4.4.1: Subgroup analyses of different assessors of outcome indicators on total symptoms

Study or Subgroup	Experimental Mean	Experimental SD	Experimental Total	Control Mean	Control SD	Control Total	Weight	Std. Mean Difference IV, Random, 95% CI
**7.2.1 caregiver**								
Momtazmanesh 2020 week 10 (ABC)	-7.7	1.06	30	-4.23	0.89	30	6.6%	-3.50 [-4.32, -2.68]
Momtazmanesh 2020 week 5 (ABC)	-3.97	0.51	30	-2.87	0.69	30	7.4%	-1.79 [-2.39, -1.18]
Politte 2020 week 12 (ABC)	-0.6	27.92	24	0.5	17.64	24	7.5%	-0.05 [-0.61, 0.52]
Politte 2020 week 4 (ABC)	-1.3	27.92	24	1.3	17.64	24	7.5%	-0.11 [-0.68, 0.46]
Politte 2020 week 8 (ABC)	-1.7	27.92	24	1.7	17.64	24	7.5%	-0.14 [-0.71, 0.42]
Zimmerman 2021 week 15 (ABC)	-8.75	10.12	12	-0.92	6.6	12	6.5%	-0.88 [-1.73, -0.04]
Zimmerman 2021 week 7 (ABC)	-2.07	7.13	15	-4.13	4.85	15	7.0%	0.33 [-0.39, 1.05]
**Subtotal (95% CI)**			**159**			**159**	**49.9%**	**-0.86 [-1.75, 0.03]**
Heterogeneity: Tau² = 1.32; Chi² = 78.02, df = 6 (P < 0.00001); I² = 92%								
Test for overall effect: Z = 1.90 (P = 0.06)								
**7.2.2 professional**								
Singh 2014 week 10 (OACIS-I)	3.46	0.81	26	3.71	0.61	14	7.2%	-0.33 [-0.98, 0.33]
Singh 2014 week 10 (OACIS-S)	-0.56	4.59	26	-0.33	4.23	14	7.2%	-0.05 [-0.70, 0.60]
Singh 2014 week 18 (OACIS-I)	3.5	0.86	26	3.93	0.62	14	7.2%	-0.54 [-1.20, 0.13]
Singh 2014 week 18 (OACIS-S)	-0.52	5.41	26	-0.11	3.74	14	7.2%	-0.08 [-0.73, 0.57]
Singh 2014 week 4 (OACIS-S)	-0.6	5.3	26	-0.04	3.59	14	7.2%	-0.11 [-0.76, 0.54]
Zimmerman 2021 week 15 (OACIS-I)	0.35	0.49	17	0.76	0.83	17	7.1%	-0.59 [-1.28, 0.10]
Zimmerman 2021 week 7 (OACIS-I)	0.24	0.44	17	0.59	0.71	17	7.1%	-0.58 [-1.27, 0.11]
**Subtotal (95% CI)**			**164**			**104**	**50.1%**	**-0.32 [-0.57, -0.07]**
Heterogeneity: Tau² = 0.00; Chi² = 3.09, df = 6 (P = 0.80); I² = 0%								
Test for overall effect: Z = 2.47 (P = 0.01)								
**Total (95% CI)**			**323**			**263**	**100.0%**	**-0.58 [-1.03, -0.13]**
Heterogeneity: Tau² = 0.62; Chi² = 85.46, df = 13 (P < 0.00001); I² = 85%								
Test for overall effect: Z = 2.54 (P = 0.01)								
Test for subgroup differences: Chi² = 1.33, df = 1 (P = 0.25), I² = 25.0%								

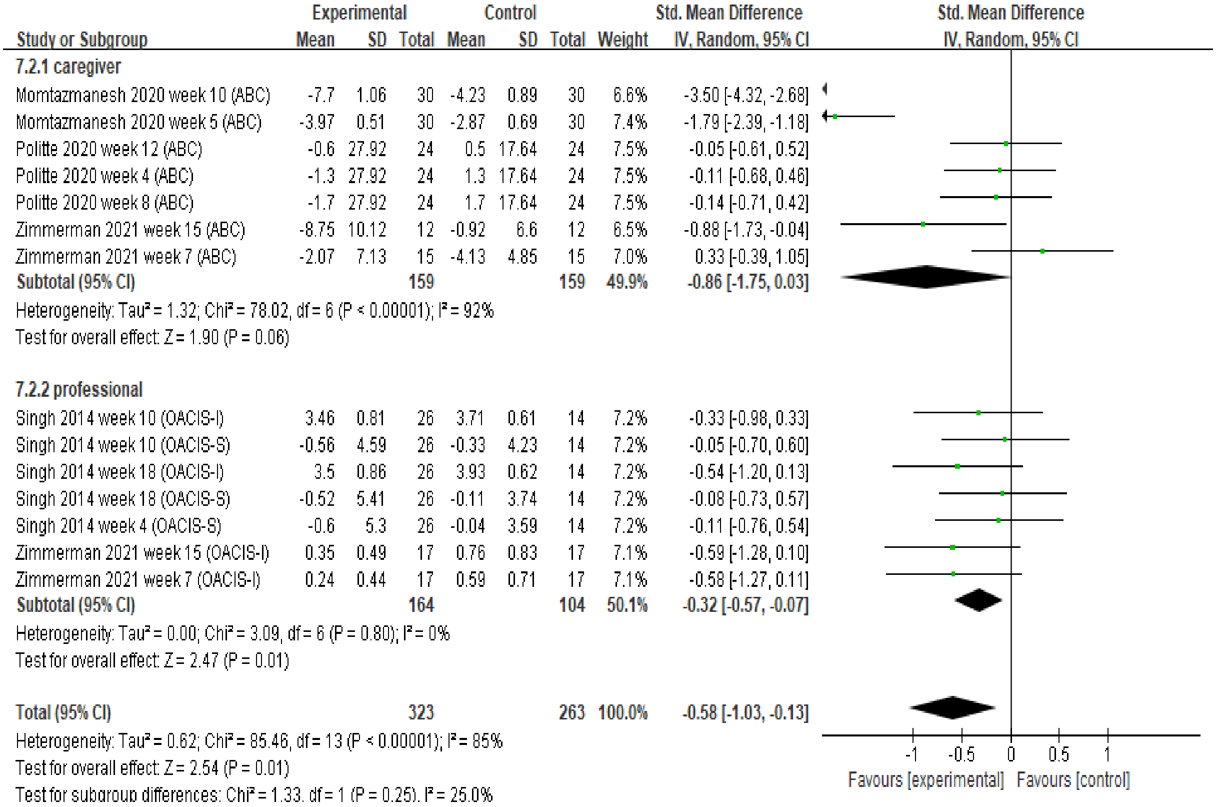

Supplementary Figure 4.4.2: Subgroup analyses of different assessors of outcome indicators on hyperactivity

EXCLI Journal 2025;24:542-557 – ISSN 1611-2156
Received: January 31, 2025, accepted: April 02, 2025, published: April 07, 2025

Study or Subgroup	Experimental Mean	SD	Total	Control Mean	SD	Total	Weight	Std. Mean Difference IV, Fixed, 95% CI
**7.4.1 caregiver**								
Politte 2020 week 12 (SRS)	0.1	32.33	24	0	20.09	24	4.2%	0.00 [-0.56, 0.57]
Politte 2020 week 4 (SRS)	-1.6	32.33	24	1.6	20.09	24	4.2%	-0.12 [-0.68, 0.45]
Politte 2020 week 8 (SRS)	-1.4	32.33	24	1.5	20.09	24	4.2%	-0.11 [-0.67, 0.46]
Zimmerman 2021 week 15 (SRS) C	-4.25	4.77	12	-3.92	7.27	12	2.1%	-0.05 [-0.85, 0.75]
Zimmerman 2021 week 15 (SRS) I	-9.75	12.34	12	-9	12.2	12	2.1%	-0.06 [-0.86, 0.74]
Zimmerman 2021 week 7 (SRS) C	0	5.62	15	-3.47	5.9	15	2.5%	0.59 [-0.15, 1.32]
Zimmerman 2021 week 7 (SRS) I	1.8	12.18	15	-7.07	11.68	15	2.5%	0.72 [-0.02, 1.47]
**Subtotal (95% CI)**			**126**			**126**	**21.9%**	**0.10 [-0.15, 0.34]**
Heterogeneity: Chi² = 5.87, df = 6 (P = 0.44); I² = 0%								
Test for overall effect: Z = 0.75 (P = 0.45)								
**7.4.2 professional**								
Magner 2023 week 18 (ADOS)	-0.7	1.16	15	-1.3	1.8	13	2.4%	0.39 [-0.36, 1.14]
Magner 2023 week 36 (ADOS)	0.2	1.55	15	-0.1	1.8	13	2.4%	0.17 [-0.57, 0.92]
Ou 2022 week 12 (OARS-4)	-0.37	0.5	51	-0.05	0.46	43	7.8%	-0.66 [-1.08, -0.24]
Ou 2022 week 4 (OARS-4)	-0.06	0.43	51	0.08	0.46	43	8.1%	-0.31 [-0.72, 0.10]
Ou 2022 week 8 (OARS-4)	-0.21	0.57	51	-0.01	0.46	43	8.1%	-0.38 [-0.79, 0.03]
Singh 2014 week 10 (OACIS-I) N	3.58	0.76	26	3.92	0.28	14	3.1%	-0.52 [-1.18, 0.14]
Singh 2014 week 10 (OACIS-I) V	3.16	0.83	26	3.36	0.84	14	3.2%	-0.24 [-0.89, 0.42]
Singh 2014 week 10 (OACIS-S) N	-0.37	4.69	26	-0.04	3.7	14	3.2%	-0.07 [-0.72, 0.58]
Singh 2014 week 10 (OACIS-S) V	-0.72	5.2	26	-0.32	6.21	14	3.2%	-0.07 [-0.72, 0.58]
Singh 2014 week 18 (OACIS-I) N	3.54	0.764	26	4.07	0.27	14	3.0%	-0.81 [-1.49, -0.14]
Singh 2014 week 18 (OACIS-I) V	2.96	0.92	26	3.86	0.53	14	2.8%	-1.09 [-1.79, -0.39]
Singh 2014 week 18 (OACIS-S) N	-0.41	5	26	0.16	3.55	14	3.2%	-0.12 [-0.77, 0.53]
Singh 2014 week 18 (OACIS-S) V	-0.91	5.25	26	0.18	5.05	14	3.2%	-0.21 [-0.86, 0.45]
Singh 2014 week 4 (OACIS-I) N	3.69	0.62	26	3.93	0.27	14	3.1%	-0.45 [-1.10, 0.21]
Singh 2014 week 4 (OACIS-I) V	3.15	0.92	26	3.57	0.76	14	3.1%	-0.47 [-1.13, 0.18]
Singh 2014 week 4 (OACIS-S) N	-0.25	4.59	26	0.02	3.67	14	3.2%	-0.06 [-0.71, 0.59]
Singh 2014 week 4 (OACIS-S) V	-0.72	5.76	26	-0.1	5.84	14	3.2%	-0.10 [-0.76, 0.55]
Zimmerman 2021 week 15 (OACIS-I) N	0.29	0.69	17	0.24	0.44	17	3.0%	0.08 [-0.59, 0.76]
Zimmerman 2021 week 15 (OACIS-I) V	0.41	0.71	17	0.79	0.77	17	2.9%	-0.50 [-1.19, 0.18]
Zimmerman 2021 week 7 (OACIS-I) N	0.18	0.53	17	0.29	0.47	17	3.0%	-0.21 [-0.89, 0.46]
Zimmerman 2021 week 7 (OACIS-I) V	0.24	0.56	17	0.59	0.62	17	2.9%	-0.58 [-1.27, 0.11]
**Subtotal (95% CI)**			**563**			**391**	**78.1%**	**-0.33 [-0.46, -0.20]**
Heterogeneity: Chi² = 20.17, df = 20 (P = 0.45); I² = 1%								
Test for overall effect: Z = 4.88 (P < 0.00001)								
**Total (95% CI)**			**689**			**517**	**100.0%**	**-0.24 [-0.35, -0.12]**
Heterogeneity: Chi² = 34.74, df = 27 (P = 0.15); I² = 22%								
Test for overall effect: Z = 3.96 (P < 0.0001)								
Test for subgroup differences: Chi² = 8.70, df = 1 (P = 0.003), I² = 88.5%								

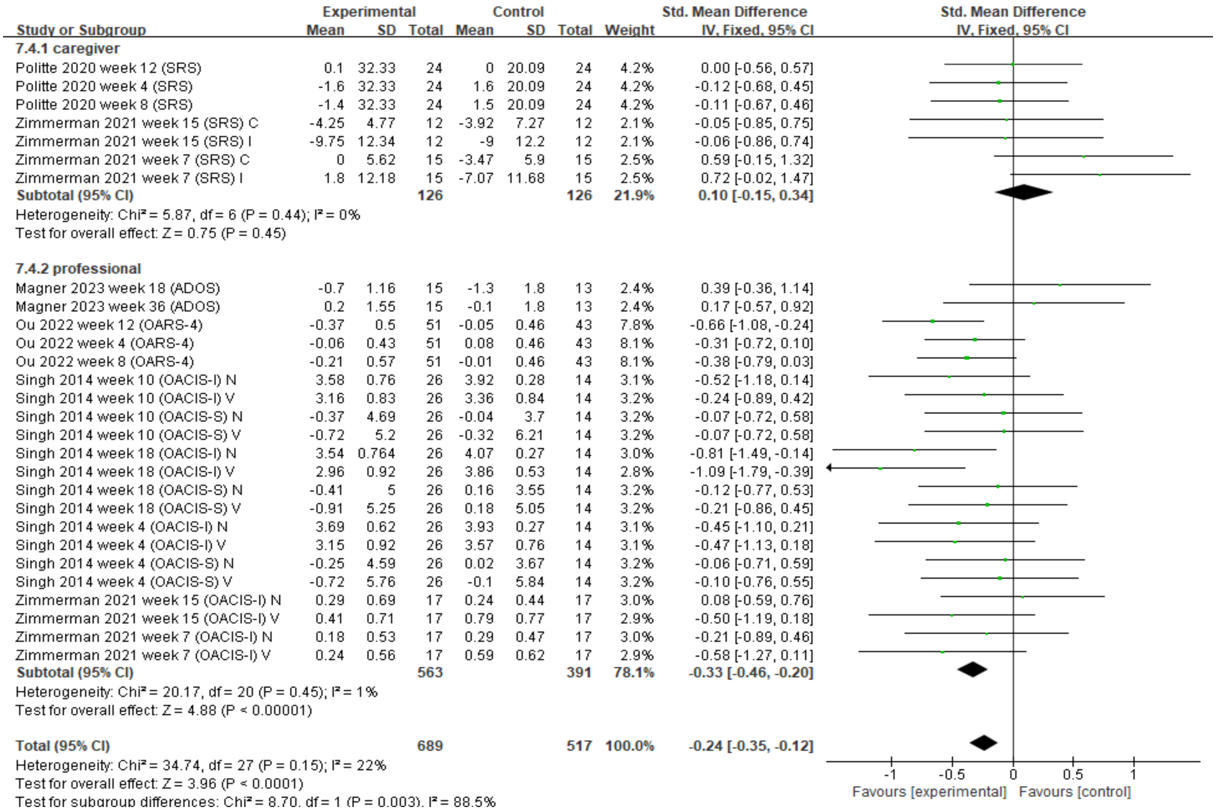

Supplementary Figure 4.4.3: Subgroup analyses of different assessors of outcome indicators on social communication

Study or Subgroup	Experimental Mean	SD	Total	Control Mean	SD	Total	Weight	Std. Mean Difference IV, Random, 95% CI
**7.3.1 caregiver**								
Momtazmanesh 2020 week 10 (ABC)	-2.87	0.6	30	-1.97	0.45	30	2.7%	-1.68 [-2.27, -1.08]
Momtazmanesh 2020 week 5 (ABC)	-2.13	0.45	30	-1.23	0.31	30	2.5%	-2.30 [-2.96, -1.64]
Ou 2022 week 12 (RBS-R)	-3.54	14.27	48	-9.82	14.22	41	3.5%	0.44 [0.02, 0.86]
Ou 2022 week 4 (RBS-R)	-3.23	10.88	48	-6.22	10.76	41	3.5%	0.27 [-0.15, 0.69]
Ou 2022 week 8 (RBS-R)	-4.65	13.37	48	-4.29	13.58	41	3.5%	-0.03 [-0.44, 0.39]
Politte 2020 week 12 (ABC)	0	15.68	24	0	9.8	24	2.9%	0.00 [-0.57, 0.57]
Politte 2020 week 12 (RBS-R)	0.3	68.59	24	-0.3	42.62	24	2.9%	0.01 [-0.56, 0.58]
Politte 2020 week 12 (SRS)	0.1	22.05	24	-0.1	13.72	24	2.9%	0.01 [-0.56, 0.58]
Politte 2020 week 4 (ABC)	-0.2	15.68	24	0.2	9.8	24	2.9%	-0.03 [-0.60, 0.54]
Politte 2020 week 4 (RBS-R)	-1.3	68.59	24	0.2	42.62	24	2.9%	-0.03 [-0.59, 0.54]
Politte 2020 week 4 (SRS)	-0.5	22.05	24	0.6	13.72	24	2.9%	-0.06 [-0.62, 0.51]
Politte 2020 week 8 (ABC)	-0.3	15.68	24	0.3	9.8	24	2.9%	-0.05 [-0.61, 0.52]
Politte 2020 week 8 (RBS-R)	0.3	68.59	24	-0.3	42.62	24	2.9%	0.01 [-0.56, 0.58]
Politte 2020 week 8 (SRS)	-0.9	22.05	24	0.9	13.72	24	2.9%	-0.10 [-0.66, 0.47]
Zimmerman 2021 week 15 (ABC)	-0.15	0.63	12	-0.13	0.39	12	2.1%	-0.04 [-0.84, 0.76]
Zimmerman 2021 week 15 (SRS)	-4.25	6.63	12	-0.75	4.79	12	2.0%	-0.58 [-1.40, 0.24]
Zimmerman 2021 week 7 (ABC)	-0.3	1.07	15	-0.27	0.62	15	2.3%	-0.03 [-0.75, 0.68]
Zimmerman 2021 week 7 (SRS)	-0.33	6.1	15	-2.67	5.17	15	2.3%	0.40 [-0.32, 1.13]
**Subtotal (95% CI)**			**474**			**453**	**49.9%**	**-0.19 [-0.49, 0.10]**
Heterogeneity: Tau² = 0.32; Chi² = 82.81, df = 17 (P < 0.00001); I² = 79%								
Test for overall effect: Z = 1.29 (P = 0.20)								
**7.3.2 professional**								
Magner 2023 week 18 (ADOS)	-0.2	1.94	15	0.08	2.16	13	2.2%	-0.13 [-0.88, 0.61]
Magner 2023 week 36 (ADOS)	0.5	1.94	15	0.7	1.44	13	2.2%	-0.11 [-0.86, 0.63]
Ou 2022 week 12 (OARS-4)	-0.04	1.57	51	0.37	1.51	43	3.5%	-0.26 [-0.67, 0.14]
Ou 2022 week 4 (OARS-4)	-0.14	1.14	51	-0.06	0.39	43	3.5%	-0.09 [-0.50, 0.32]
Ou 2022 week 8 (OARS-4)	0.07	1.14	51	0.19	1.05	43	3.5%	-0.11 [-0.51, 0.30]
Singh 2014 week 10 (OACIS-I) I	3.46	0.71	26	3.79	0.43	14	2.5%	-0.51 [-1.18, 0.15]
Singh 2014 week 10 (OACIS-S) B	-0.52	5.86	26	0.01	3.18	14	2.5%	-0.10 [-0.75, 0.55]
Singh 2014 week 10 (OACIS-S) I	-0.49	4.39	26	-0.33	3.26	14	2.5%	-0.04 [-0.69, 0.61]
Singh 2014 week 18 (OACIS-I) B	3.42	0.86	26	4	0.39	14	2.5%	-0.77 [-1.45, -0.10]
Singh 2014 week 18 (OACIS-S) B	-0.45	5.3	26	0.01	2.99	14	2.5%	-0.10 [-0.75, 0.55]
Singh 2014 week 18 (OACIS-S) I	-0.49	4.84	26	-0.12	3.07	14	2.5%	-0.08 [-0.73, 0.57]
Singh 2014 week 4 (OACIS-I) B	3.81	0.57	26	3.71	0.61	14	2.5%	0.17 [-0.48, 0.82]
Singh 2014 week 4 (OACIS-I) I	3.81	0.49	26	3.86	0.53	14	2.5%	-0.10 [-0.75, 0.55]
Singh 2014 week 4 (OACIS-S) B	-0.06	5.56	26	-0.27	3.7	14	2.5%	0.04 [-0.61, 0.69]
Singh 2014 week 4 (OACIS-S) I	-0.14	4.28	26	-0.26	2.73	14	2.5%	0.03 [-0.62, 0.68]
Zimmerman 2021 week 15 (OACIS-I) B	0.06	0.43	17	0.12	0.49	17	2.5%	-0.13 [-0.80, 0.55]
Zimmerman 2021 week 15 (OACIS-I) I	0.12	0.49	17	0.24	0.56	17	2.4%	-0.22 [-0.90, 0.45]
Zimmerman 2021 week 7 (OACIS-I) B	0.12	0.33	17	0.12	0.33	17	2.5%	0.00 [-0.67, 0.67]
Zimmerman 2021 week 7 (OACIS-I) I	0.06	0.24	17	0.06	0.24	17	2.5%	0.00 [-0.67, 0.67]
**Subtotal (95% CI)**			**511**			**363**	**50.1%**	**-0.14 [-0.27, 0.00]**
Heterogeneity: Tau² = 0.00; Chi² = 7.06, df = 18 (P = 0.99); I² = 0%								
Test for overall effect: Z = 1.95 (P = 0.05)								
**Total (95% CI)**			**985**			**816**	**100.0%**	**-0.16 [-0.31, -0.00]**
Heterogeneity: Tau² = 0.13; Chi² = 89.89, df = 36 (P < 0.00001); I² = 60%								
Test for overall effect: Z = 2.01 (P = 0.04)								
Test for subgroup differences: Chi² = 0.13, df = 1 (P = 0.72), I² = 0%								

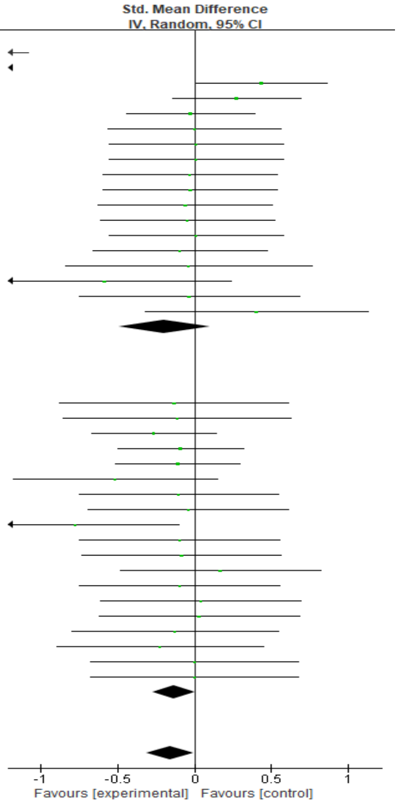

Supplementary Figure 4.4.4: Subgroup analyses of different assessors of outcome indicators on restricted interests and repetitive behavior

Supplementary Figure 4.5: Subgroup analyses of different intervention duration

Study or Subgroup	Experimental Mean	Experimental SD	Experimental Total	Control Mean	Control SD	Control Total	Weight	Std. Mean Difference IV, Random, 95% CI
**4.14.1 <10 weeks**								
Ou 2022 week 4 (OACIS-I)	3.61	0.56	49	3.79	0.52	42	5.0%	-0.33 [-0.74, 0.09]
Ou 2022 week 4 (OACIS-S)	0.13	0.71	51	0.17	0.72	43	5.0%	-0.06 [-0.46, 0.35]
Ou 2022 week 4 (OARS-4)	-0.12	0.36	51	-0.01	0.33	43	5.0%	-0.31 [-0.72, 0.09]
Ou 2022 week 8 (OACIS-I)	3.26	0.56	49	3.71	0.52	42	4.8%	-0.82 [-1.25, -0.39]
Ou 2022 week 8 (OACIS-S)	0.1	0.71	51	0.09	0.72	43	5.0%	0.01 [-0.39, 0.42]
Ou 2022 week 8 (OARS-4)	-0.23	0.36	51	-0.03	0.33	43	5.0%	-0.57 [-0.99, -0.16]
Politte 2020 week 4 (OACIS-S)	-0.04	2.94	24	0.04	1.47	24	3.7%	-0.03 [-0.60, 0.53]
Politte 2020 week 8 (OACIS-S)	-0.03	2.94	24	0.02	1.47	24	3.7%	-0.02 [-0.59, 0.54]
Singh 2014 week 4 (ABC)	-12.69	21.26	26	0.77	6.63	13	3.0%	-0.74 [-1.43, -0.05]
Singh 2014 week 4 (OACIS-I)	4.04	0.2	26	4.14	0.53	14	3.2%	-0.28 [-0.93, 0.37]
Singh 2014 week 4 (OACIS-S)	0	3.62	26	-0.17	2.96	14	3.2%	0.05 [-0.60, 0.70]
Zimmerman 2021 week 7 (ABC)	-2	18.68	15	-11	16.99	15	2.8%	0.49 [-0.24, 1.22]
Zimmerman 2021 week 7 (OACIS-I)	0.29	0.69	17	0.18	0.39	17	3.1%	0.19 [-0.48, 0.87]
**Subtotal (95% CI)**			**460**			**377**	**52.6%**	**-0.22 [-0.41, -0.03]**
Heterogeneity: Tau² = 0.05; Chi² = 21.46, df = 12 (P = 0.04); I² = 44%								
Test for overall effect: Z = 2.29 (P = 0.02)								
**4.14.2 ≥10 weeks**								
Magner 2023 week 18 (ABC)	-4.9	16.27	15	-8.1	22.35	13	2.7%	0.16 [-0.58, 0.90]
Magner 2023 week 36 (ABC)	1.6	12.39	15	-1	14.06	13	2.7%	0.19 [-0.55, 0.94]
Ou 2022 week 12 (OACIS-I)	2.89	0.63	49	3.51	0.58	42	4.8%	-1.01 [-1.45, -0.57]
Ou 2022 week 12 (OACIS-S)	-0.17	0.71	51	0.01	0.79	43	5.0%	-0.24 [-0.65, 0.17]
Ou 2022 week 12 (OARS-4)	-0.33	0.36	51	-0.09	0.39	43	5.0%	-0.64 [-1.05, -0.22]
Politte 2020 week 12 (OACIS-S)	-0.02	2.94	24	0	1.47	24	3.7%	-0.01 [-0.57, 0.56]
Singh 2014 week 10 (ABC)	-19.61	27.91	22	3.31	12.62	13	2.8%	-0.95 [-1.68, -0.23]
Singh 2014 week 10 (OACIS-I)	3.88	0.33	26	3.86	0.36	14	3.2%	0.06 [-0.59, 0.71]
Singh 2014 week 10 (OACIS-S)	-0.07	3.42	26	-0.1	2.99	14	3.2%	0.01 [-0.64, 0.66]
Singh 2014 week 18 (ABC)	-21.44	21.7	25	-2	14.52	10	2.6%	-0.95 [-1.72, -0.18]
Singh 2014 week 18 (OACIS-I)	3.81	0.4	26	3.93	0.27	14	3.2%	-0.33 [-0.98, 0.33]
Singh 2014 week 18 (OACIS-S)	0.04	3.42	26	-0.03	2.81	14	3.2%	0.02 [-0.63, 0.67]
Zimmerman 2021 week 15 (ABC)	-19.5	18.47	12	-2.92	16.1	12	2.3%	-0.92 [-1.77, -0.07]
Zimmerman 2021 week 15 (OACIS-I)	0.29	0.59	17	0.24	0.56	17	3.1%	0.08 [-0.59, 0.76]
**Subtotal (95% CI)**			**385**			**286**	**47.4%**	**-0.33 [-0.57, -0.09]**
Heterogeneity: Tau² = 0.11; Chi² = 28.32, df = 13 (P = 0.008); I² = 54%								
Test for overall effect: Z = 2.70 (P = 0.007)								
**Total (95% CI)**			**845**			**663**	**100.0%**	**-0.27 [-0.42, -0.12]**
Heterogeneity: Tau² = 0.07; Chi² = 51.11, df = 26 (P = 0.002); I² = 49%								
Test for overall effect: Z = 3.56 (P = 0.0004)								
Test for subgroup differences: Chi² = 0.49, df = 1 (P = 0.48), I² = 0%								

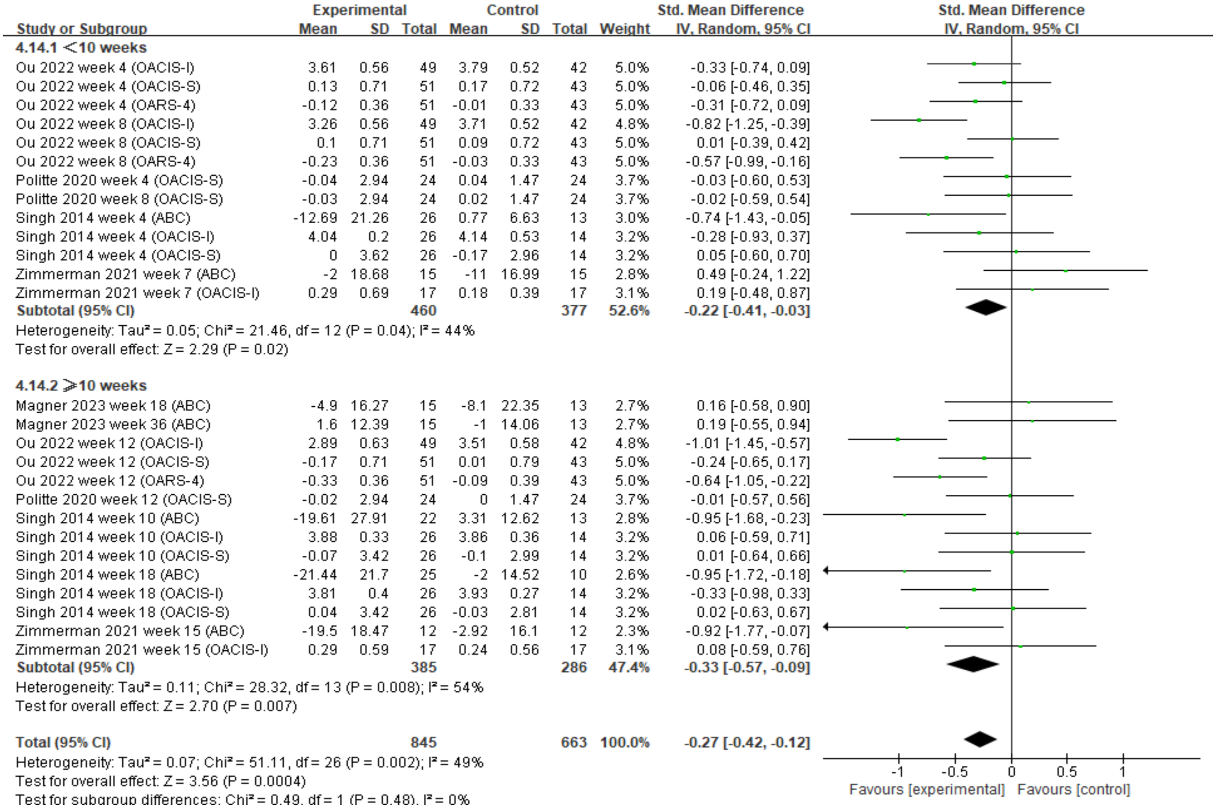

Supplementary Figure 4.5.1: Subgroup analyses of different intervention duration on total symptoms

Study or Subgroup	Experimental Mean	Experimental SD	Experimental Total	Control Mean	Control SD	Control Total	Weight	Std. Mean Difference IV, Fixed, 95% CI
**4.6.1 <10 weeks**								
Singh 2014 week 4 (OACIS-I)	3.42	0.99	26	3.86	0.66	14	12.8%	-0.48 [-1.14, 0.18]
Singh 2014 week 4 (OACIS-S)	-0.48	5.66	26	0.16	5.09	14	13.2%	-0.11 [-0.76, 0.54]
Zimmerman 2021 week 7 (OACIS-I)	0.06	0.43	17	0.24	0.56	17	12.1%	-0.35 [-1.03, 0.33]
**Subtotal (95% CI)**			**69**			**45**	**38.1%**	**-0.31 [-0.70, 0.07]**
Heterogeneity: Chi² = 0.63, df = 2 (P = 0.73); I² = 0%								
Test for overall effect: Z = 1.61 (P = 0.11)								
**4.6.2 ≥10 weeks**								
Singh 2014 week 10 (OACIS-I)	2.96	1	26	3.79	0.58	14	11.9%	-0.93 [-1.61, -0.24]
Singh 2014 week 10 (OACIS-S)	-0.94	5.02	26	0.09	4.68	14	13.1%	-0.21 [-0.86, 0.45]
Singh 2014 week 18 (OACIS-I)	2.88	0.99	26	3.93	0.73	14	11.4%	-1.13 [-1.83, -0.43]
Singh 2014 week 18 (OACIS-S)	-1.02	5.56	26	0.23	4.86	14	13.1%	-0.23 [-0.88, 0.42]
Zimmerman 2021 week 15 (OACIS-I)	0.24	0.44	17	0.29	0.69	17	12.3%	-0.08 [-0.76, 0.59]
**Subtotal (95% CI)**			**121**			**73**	**61.9%**	**-0.50 [-0.80, -0.20]**
Heterogeneity: Chi² = 7.52, df = 4 (P = 0.11); I² = 47%								
Test for overall effect: Z = 3.23 (P = 0.001)								
**Total (95% CI)**			**190**			**118**	**100.0%**	**-0.43 [-0.66, -0.19]**
Heterogeneity: Chi² = 8.68, df = 7 (P = 0.28); I² = 19%								
Test for overall effect: Z = 3.54 (P = 0.0004)								
Test for subgroup differences: Chi² = 0.53, df = 1 (P = 0.47), I² = 0%								

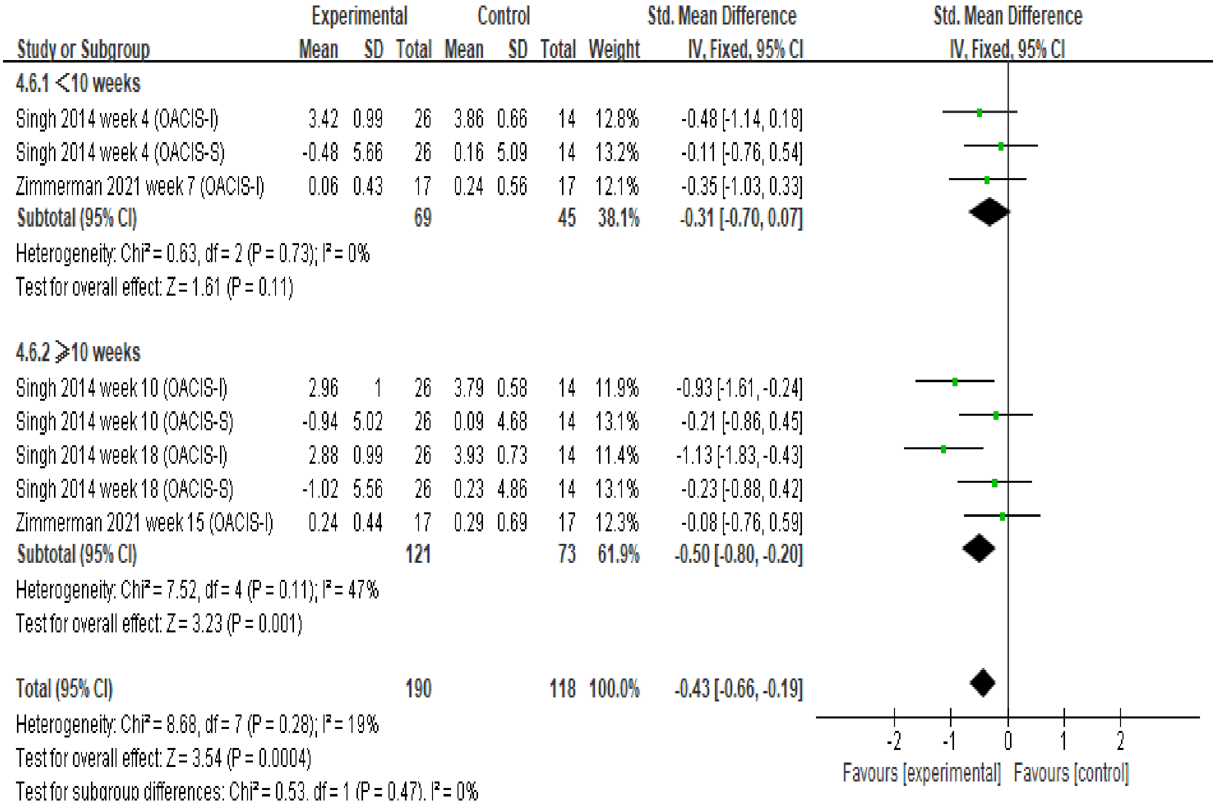

Supplementary Figure 4.5.2: Subgroup analyses of different intervention duration on aberrant behavior

EXCLI Journal 2025;24:542-557 – ISSN 1611-2156
Received: January 31, 2025, accepted: April 02, 2025, published: April 07, 2025

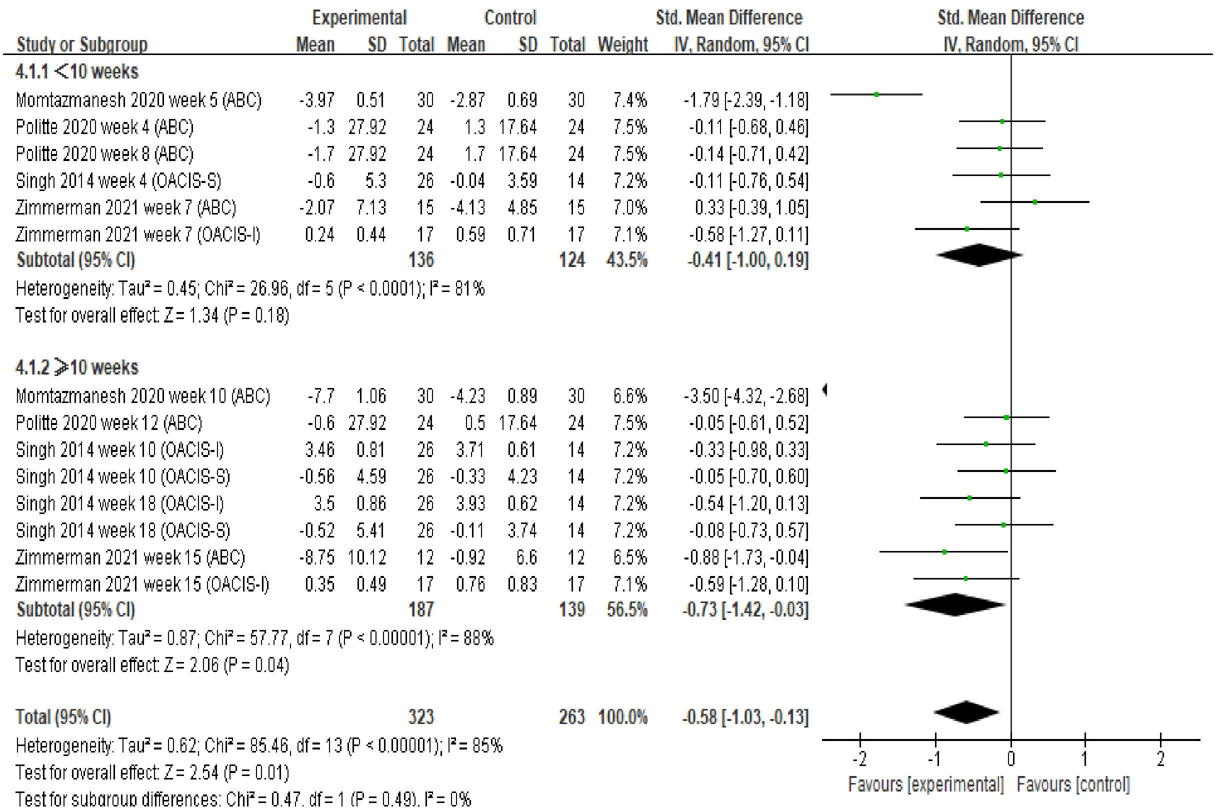

Supplementary Figure 4.5.3: Subgroup analyses of different intervention duration on hyperactivity

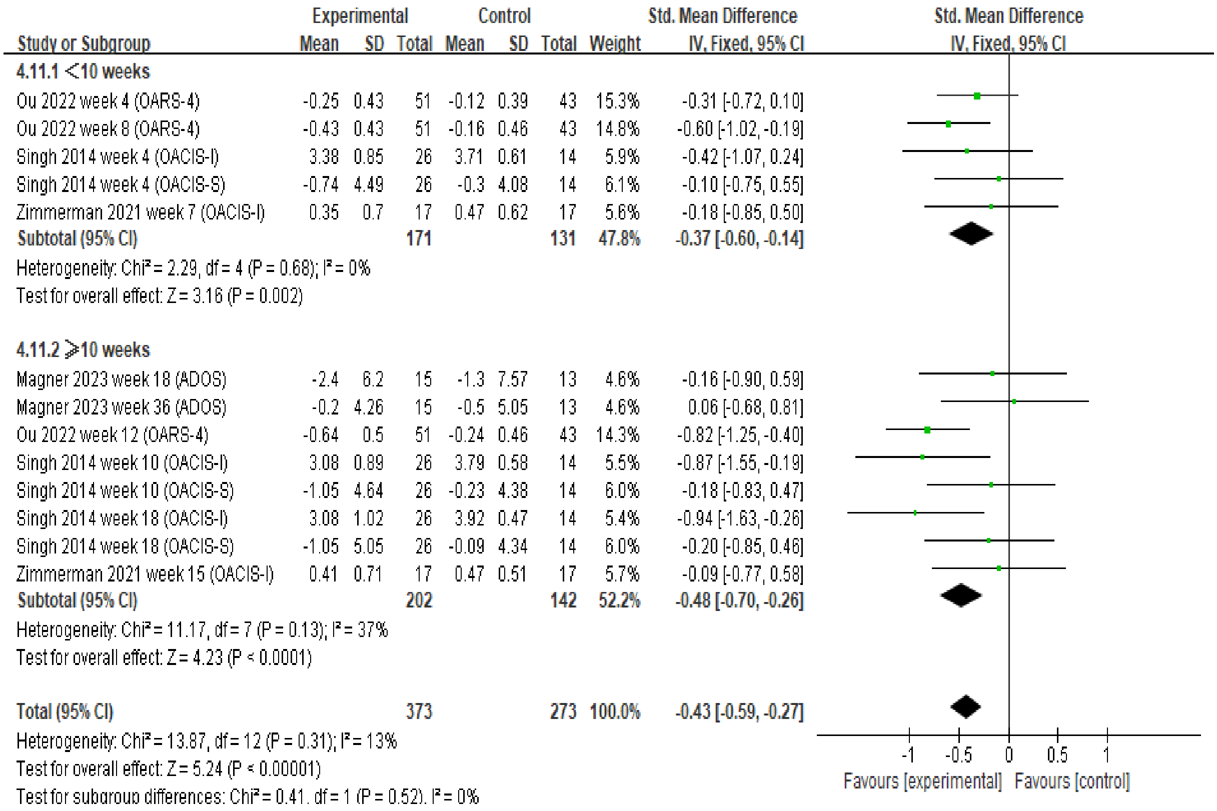

Supplementary Figure 4.5.4: Subgroup analyses of different intervention duration on social interaction

Study or Subgroup	Experimental Mean	Experimental SD	Experimental Total	Control Mean	Control SD	Control Total	Weight	Std. Mean Difference IV, Fixed, 95% CI
**4.12.1 <10 weeks**								
Ou 2022 week 4 (OARS-4)	-0.06	0.43	51	0.08	0.46	43	8.1%	-0.31 [-0.72, 0.10]
Ou 2022 week 8 (OARS-4)	-0.21	0.57	51	-0.01	0.46	43	8.1%	-0.38 [-0.79, 0.03]
Politte 2020 week 4 (SRS)	-1.6	32.33	24	1.6	20.09	24	4.2%	-0.12 [-0.68, 0.45]
Politte 2020 week 8 (SRS)	-1.4	32.33	24	1.5	20.09	24	4.2%	-0.11 [-0.67, 0.46]
Singh 2014 week 4 (OACIS-I) N	3.69	0.62	26	3.93	0.27	14	3.1%	-0.45 [-1.10, 0.21]
Singh 2014 week 4 (OACIS-I) V	3.15	0.92	26	3.57	0.76	14	3.1%	-0.47 [-1.13, 0.18]
Singh 2014 week 4 (OACIS-S) N	-0.25	4.59	26	0.02	3.67	14	3.2%	-0.06 [-0.71, 0.59]
Singh 2014 week 4 (OACIS-S) V	-0.72	5.76	26	-0.1	5.84	14	3.2%	-0.10 [-0.76, 0.55]
Zimmerman 2021 week 7 (OACIS-I) N	0.18	0.53	17	0.29	0.47	17	3.0%	-0.21 [-0.89, 0.46]
Zimmerman 2021 week 7 (OACIS-I) V	0.24	0.56	17	0.59	0.62	17	2.9%	-0.58 [-1.27, 0.11]
Zimmerman 2021 week 7 (SRS) C	0	5.62	15	-3.47	5.9	15	2.5%	0.59 [-0.15, 1.32]
Zimmerman 2021 week 7 (SRS) I	1.8	12.18	15	-7.07	11.68	15	2.5%	0.72 [-0.02, 1.47]
**Subtotal (95% CI)**			**318**			**254**	**48.1%**	**-0.19 [-0.35, -0.02]**
Heterogeneity: Chi² = 14.18, df = 11 (P = 0.22); I² = 22%								
Test for overall effect: Z = 2.18 (P = 0.03)								
**4.12.2 ≥10 weeks**								
Magner 2023 week 18 (ADOS)	-0.7	1.16	15	-1.3	1.8	13	2.4%	0.39 [-0.36, 1.14]
Magner 2023 week 36 (ADOS)	0.2	1.55	15	-0.1	1.8	13	2.4%	0.17 [-0.57, 0.92]
Ou 2022 week 12 (OARS-4)	-0.37	0.5	51	-0.05	0.46	43	7.8%	-0.66 [-1.08, -0.24]
Politte 2020 week 12 (SRS)	0.1	32.33	24	0	20.09	24	4.2%	0.00 [-0.56, 0.57]
Singh 2014 week 10 (OACIS-I) N	3.58	0.76	26	3.92	0.28	14	3.1%	-0.52 [-1.18, 0.14]
Singh 2014 week 10 (OACIS-I) V	3.16	0.83	26	3.36	0.84	14	3.2%	-0.24 [-0.89, 0.42]
Singh 2014 week 10 (OACIS-S) N	-0.37	4.69	26	-0.04	3.7	14	3.2%	-0.07 [-0.72, 0.58]
Singh 2014 week 10 (OACIS-S) V	-0.72	5.2	26	-0.32	6.21	14	3.2%	-0.07 [-0.72, 0.58]
Singh 2014 week 18 (OACIS-I) N	3.54	0.764	26	4.07	0.27	14	3.0%	-0.81 [-1.49, -0.14]
Singh 2014 week 18 (OACIS-I) V	2.96	0.92	26	3.86	0.53	14	2.8%	-1.09 [-1.79, -0.39]
Singh 2014 week 18 (OACIS-S) N	-0.41	5	26	0.16	3.55	14	3.2%	-0.12 [-0.77, 0.53]
Singh 2014 week 18 (OACIS-S) V	-0.91	5.25	26	0.18	5.05	14	3.2%	-0.21 [-0.86, 0.45]
Zimmerman 2021 week 15 (OACIS-I) N	0.29	0.69	17	0.24	0.44	17	3.0%	0.08 [-0.59, 0.76]
Zimmerman 2021 week 15 (OACIS-I) V	0.41	0.71	17	0.79	0.77	17	2.9%	-0.50 [-1.19, 0.18]
Zimmerman 2021 week 15 (SRS) C	-4.25	4.77	12	-3.92	7.27	12	2.1%	-0.05 [-0.85, 0.75]
Zimmerman 2021 week 15 (SRS) I	-9.75	12.34	12	-9	12.2	12	2.1%	-0.06 [-0.86, 0.74]
**Subtotal (95% CI)**			**371**			**263**	**51.9%**	**-0.28 [-0.44, -0.12]**
Heterogeneity: Chi² = 19.95, df = 15 (P = 0.17); I² = 25%								
Test for overall effect: Z = 3.39 (P = 0.0007)								
**Total (95% CI)**			**689**			**517**	**100.0%**	**-0.24 [-0.35, -0.12]**
Heterogeneity: Chi² = 34.74, df = 27 (P = 0.15); I² = 22%								
Test for overall effect: Z = 3.96 (P < 0.0001)								
Test for subgroup differences: Chi² = 0.61, df = 1 (P = 0.43), I² = 0%								

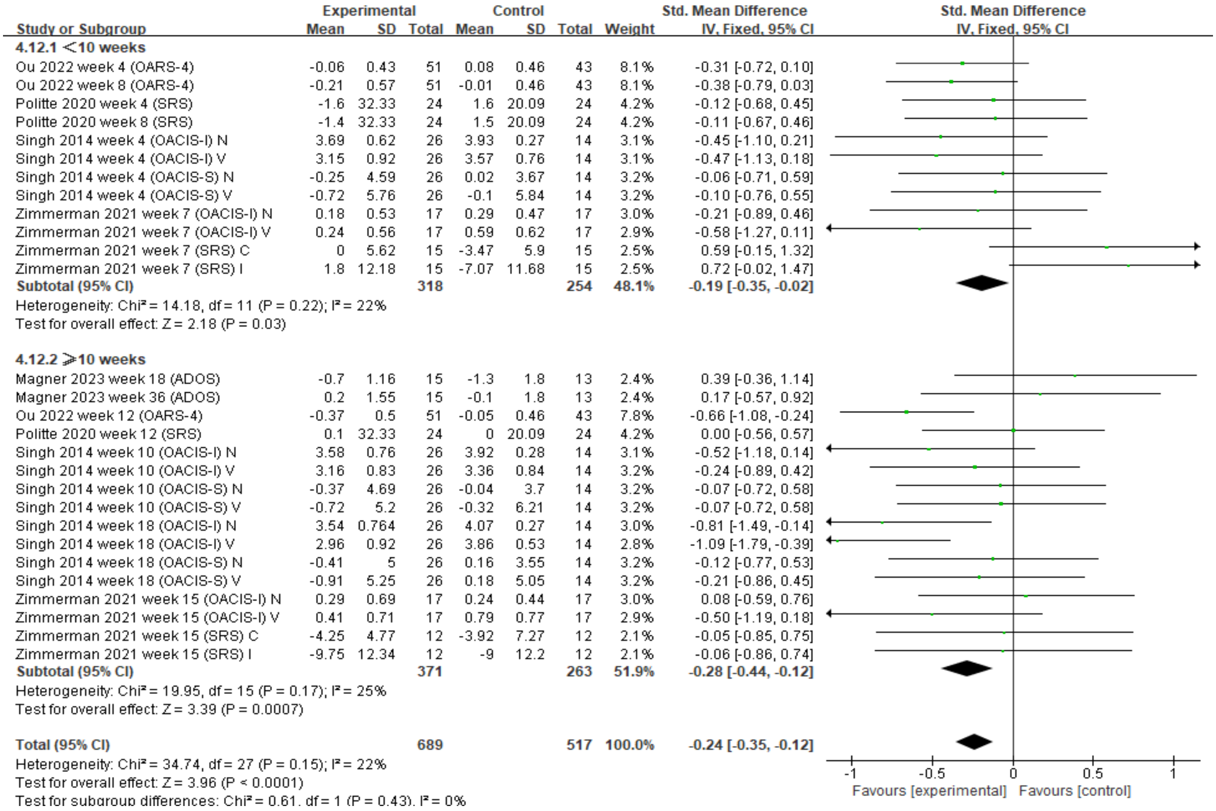

Supplementary Figure 4.5.5: Subgroup analyses of different intervention duration on social communication

Study or Subgroup	Experimental Mean	Experimental SD	Experimental Total	Control Mean	Control SD	Control Total	Weight	Std. Mean Difference IV, Random, 95% CI
**4.15.1 <10 weeks**								
Momtazmanesh 2020 week 5 (ABC)	-2.13	0.45	30	-1.23	0.31	30	2.5%	-2.30 [-2.96, -1.64]
Ou 2022 week 4 (OARS-4)	-0.14	1.14	51	-0.06	0.39	43	3.5%	-0.09 [-0.50, 0.32]
Ou 2022 week 4 (RBS-R)	-3.23	10.88	48	-6.22	10.76	41	3.5%	0.27 [-0.15, 0.69]
Ou 2022 week 8 (OARS-4)	0.07	1.14	51	0.19	1.05	43	3.5%	-0.11 [-0.51, 0.30]
Ou 2022 week 8 (RBS-R)	-4.65	13.37	48	-4.29	13.58	41	3.5%	-0.03 [-0.44, 0.39]
Politte 2020 week 4 (ABC)	-0.2	15.68	24	0.2	9.8	24	2.9%	-0.03 [-0.60, 0.54]
Politte 2020 week 4 (RBS-R)	-1.3	68.59	24	0.2	42.62	24	2.9%	-0.03 [-0.59, 0.54]
Politte 2020 week 4 (SRS)	-0.5	22.05	24	0.6	13.72	24	2.9%	-0.06 [-0.62, 0.51]
Politte 2020 week 8 (ABC)	-0.3	15.68	24	0.3	9.8	24	2.9%	-0.05 [-0.61, 0.52]
Politte 2020 week 8 (RBS-R)	0.3	68.59	24	-0.3	42.62	24	2.9%	0.01 [-0.56, 0.58]
Politte 2020 week 8 (SRS)	-0.9	22.05	24	0.9	13.72	24	2.9%	-0.10 [-0.66, 0.47]
Singh 2014 week 4 (OACIS-I) B	3.81	0.57	26	3.71	0.61	14	2.5%	0.17 [-0.48, 0.82]
Singh 2014 week 4 (OACIS-I) I	3.81	0.49	26	3.86	0.53	14	2.5%	-0.10 [-0.75, 0.55]
Singh 2014 week 4 (OACIS-S) B	-0.06	5.56	26	-0.27	3.7	14	2.5%	0.04 [-0.61, 0.69]
Singh 2014 week 4 (OACIS-S) I	-0.14	4.28	26	-0.26	2.73	14	2.5%	0.03 [-0.62, 0.68]
Zimmerman 2021 week 7 (ABC)	-0.3	1.07	15	-0.27	0.62	15	2.3%	-0.03 [-0.75, 0.68]
Zimmerman 2021 week 7 (OACIS-I) B	0.12	0.33	17	0.12	0.33	17	2.5%	0.00 [-0.67, 0.67]
Zimmerman 2021 week 7 (OACIS-I) I	0.06	0.24	17	0.06	0.24	17	2.5%	0.00 [-0.67, 0.67]
Zimmerman 2021 week 7 (SRS)	-0.33	6.1	15	-2.67	5.17	15	2.3%	0.40 [-0.32, 1.13]
**Subtotal (95% CI)**			**540**			**462**	**53.2%**	**-0.10 [-0.31, 0.12]**
Heterogeneity: Tau² = 0.14; Chi² = 48.91, df = 18 (P = 0.0001); I² = 63%								
Test for overall effect: Z = 0.88 (P = 0.38)								
**4.15.2 ≥10 weeks**								
Magner 2023 week 18 (ADOS)	-0.2	1.94	15	0.08	2.16	13	2.2%	-0.13 [-0.88, 0.61]
Magner 2023 week 36 (ADOS)	0.5	1.94	15	0.7	1.44	13	2.2%	-0.11 [-0.86, 0.63]
Momtazmanesh 2020 week 10 (ABC)	-2.87	0.6	30	-1.97	0.45	30	2.7%	-1.68 [-2.27, -1.08]
Ou 2022 week 12 (OARS-4)	-0.04	1.57	51	0.37	1.51	43	3.5%	-0.26 [-0.67, 0.14]
Ou 2022 week 12 (RBS-R)	-3.54	14.27	48	-9.82	14.22	41	3.5%	0.44 [0.02, 0.86]
Politte 2020 week 12 (ABC)	0	15.68	24	0	9.8	24	2.9%	0.00 [-0.57, 0.57]
Politte 2020 week 12 (RBS-R)	0.3	68.59	24	-0.3	42.62	24	2.9%	0.01 [-0.56, 0.58]
Politte 2020 week 12 (SRS)	0.1	22.05	24	-0.1	13.72	24	2.9%	0.01 [-0.56, 0.58]
Singh 2014 week 10 (OACIS-I) I	3.46	0.71	26	3.79	0.43	14	2.5%	-0.51 [-1.18, 0.15]
Singh 2014 week 10 (OACIS-S) B	-0.52	5.86	26	0.01	3.18	14	2.5%	-0.10 [-0.75, 0.55]
Singh 2014 week 10 (OACIS-S) I	-0.49	4.39	26	-0.33	3.26	14	2.5%	-0.04 [-0.69, 0.61]
Singh 2014 week 18 (OACIS-I) B	3.42	0.86	26	4	0.39	14	2.5%	-0.77 [-1.45, -0.10]
Singh 2014 week 18 (OACIS-S) B	-0.45	5.3	26	0.01	2.99	14	2.5%	-0.10 [-0.75, 0.55]
Singh 2014 week 18 (OACIS-S) I	-0.49	4.84	26	-0.12	3.07	14	2.5%	-0.08 [-0.73, 0.57]
Zimmerman 2021 week 15 (ABC)	-0.15	0.63	12	-0.13	0.39	12	2.1%	-0.04 [-0.84, 0.76]
Zimmerman 2021 week 15 (OACIS-I) B	0.06	0.43	17	0.12	0.49	17	2.5%	-0.13 [-0.80, 0.55]
Zimmerman 2021 week 15 (OACIS-I) I	0.12	0.49	17	0.24	0.56	17	2.4%	-0.22 [-0.90, 0.45]
Zimmerman 2021 week 15 (SRS)	-4.25	6.63	12	-0.75	4.79	12	2.0%	-0.58 [-1.40, 0.24]
**Subtotal (95% CI)**			**445**			**354**	**46.8%**	**-0.23 [-0.45, -0.00]**
Heterogeneity: Tau² = 0.13; Chi² = 39.46, df = 17 (P = 0.002); I² = 57%								
Test for overall effect: Z = 1.99 (P = 0.05)								
**Total (95% CI)**			**985**			**816**	**100.0%**	**-0.16 [-0.31, -0.00]**
Heterogeneity: Tau² = 0.13; Chi² = 89.89, df = 36 (P < 0.00001); I² = 60%								
Test for overall effect: Z = 2.01 (P = 0.04)								
Test for subgroup differences: Chi² = 0.69, df = 1 (P = 0.41), I² = 0%								

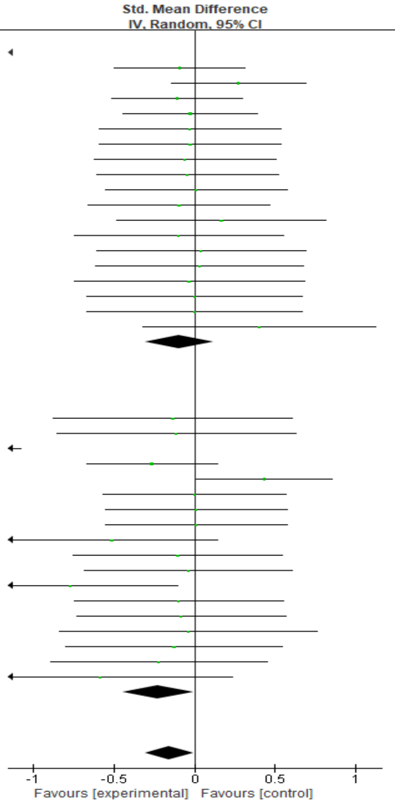

Supplementary Figure 4.5.6: Subgroup analyses of different intervention duration on restricted interests and repetitive behavior

EXCLI Journal 2025;24:542-557 – ISSN 1611-2156
Received: January 31, 2025, accepted: April 02, 2025, published: April 07, 2025

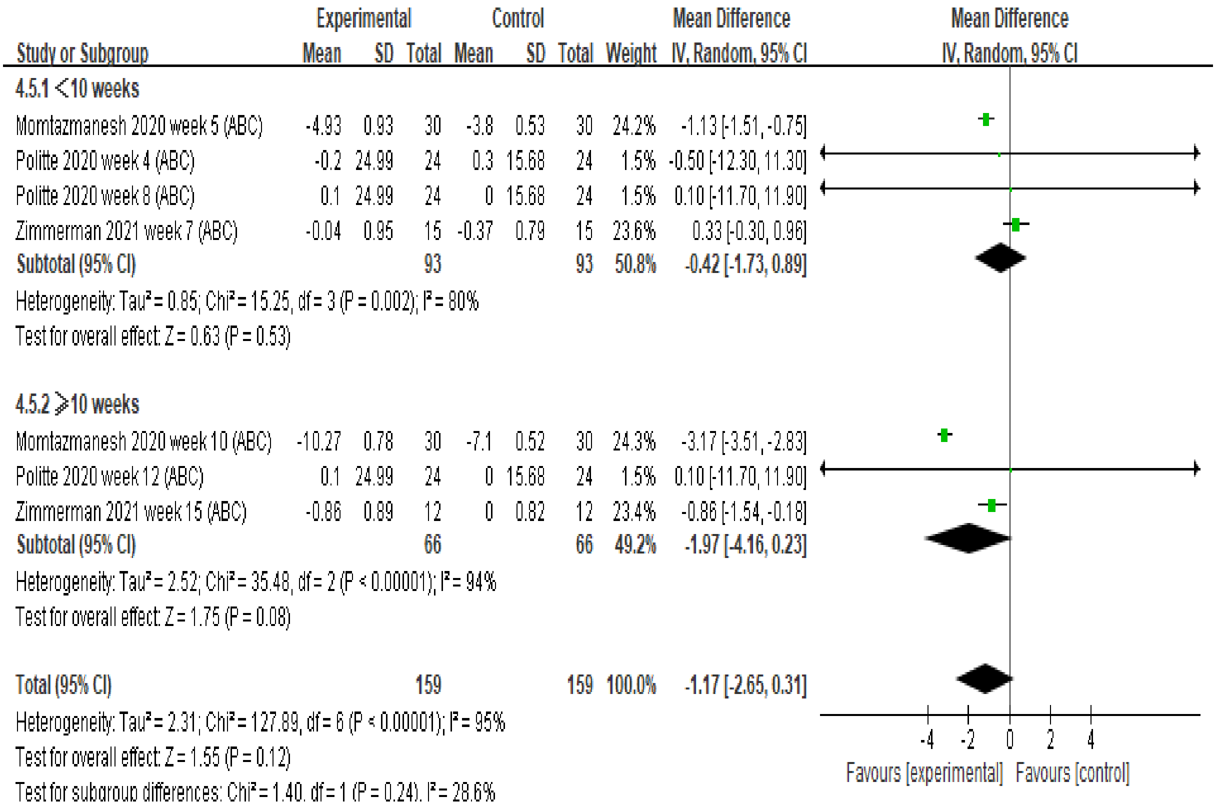

Supplementary Figure 4.5.7: Subgroup analyses of different intervention duration on irritability

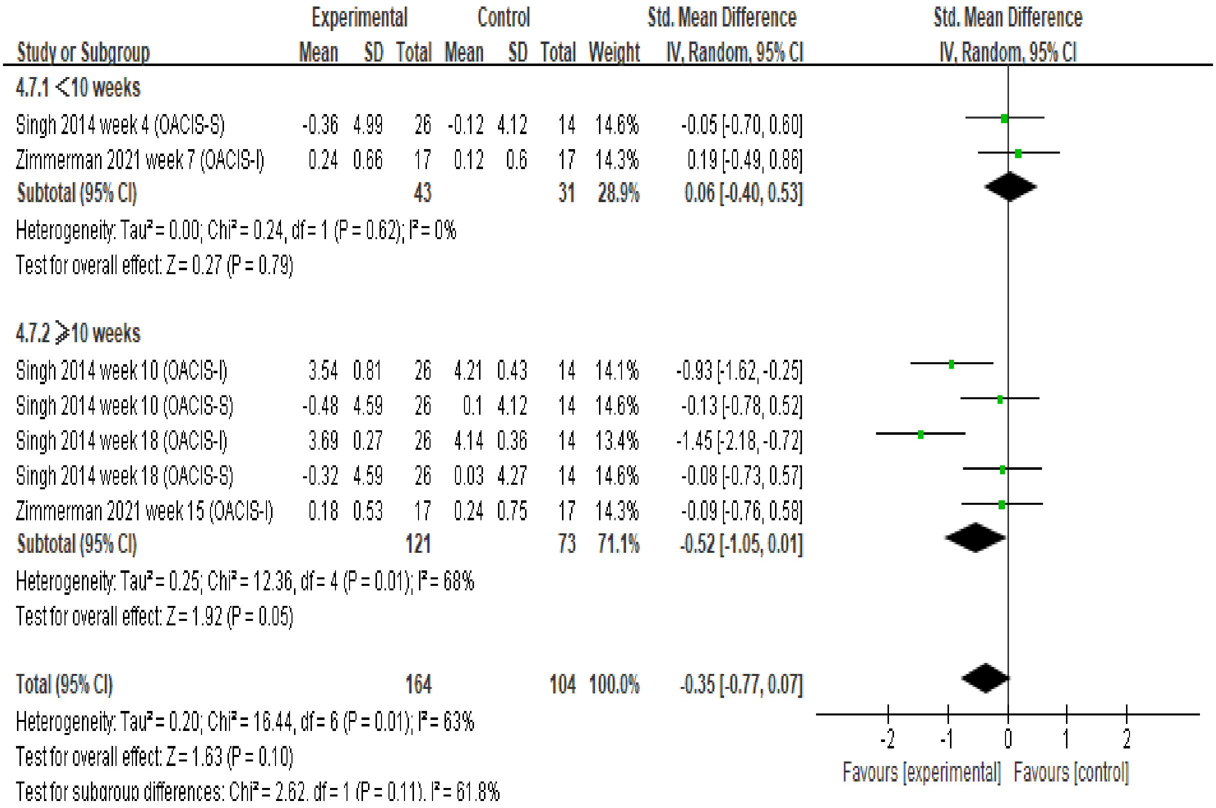

Supplementary Figure 4.5.8: Subgroup analyses of different intervention duration on anxiety

EXCLI Journal 2025;24:542-557 – ISSN 1611-2156
Received: January 31, 2025, accepted: April 02, 2025, published: April 07, 2025

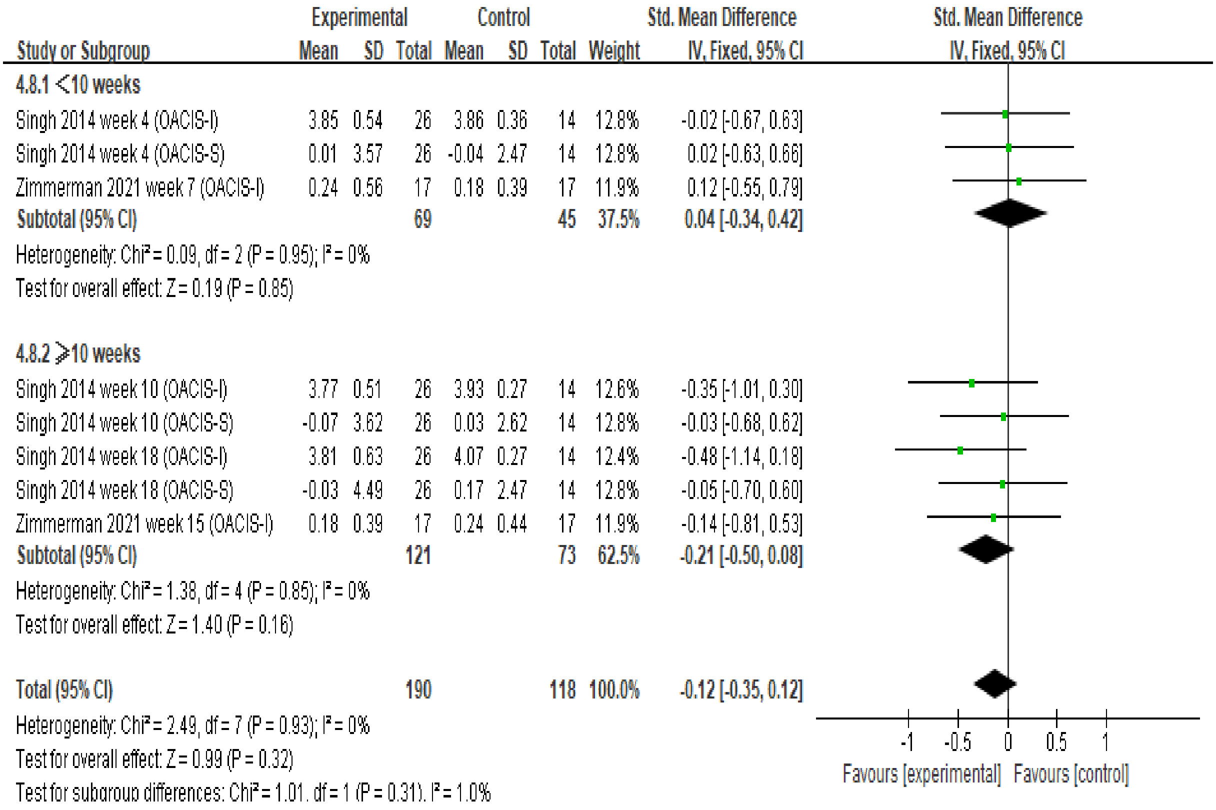

Supplementary Figure 4.5.9: Subgroup analyses of different intervention duration on sensory sensitivity

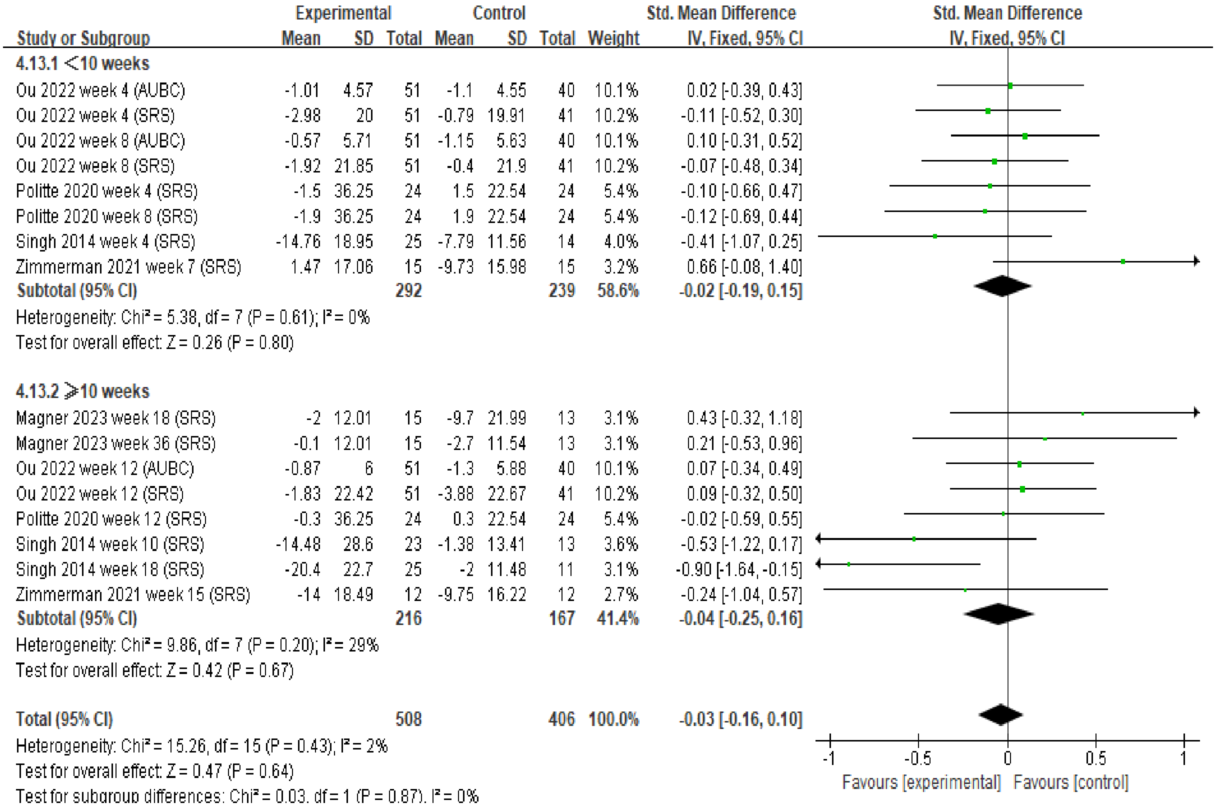

Supplementary Figure 4.5.10: Subgroup analyses of different intervention duration on total social skills

Study or Subgroup	Experimental Mean	Experimental SD	Experimental Total	Control Mean	Control SD	Control Total	Weight	Mean Difference IV, Fixed, 95% CI
**4.9.1 <10 weeks**								
Politte 2020 week 4 (SRS)	-0.4	12.25	24	0.4	7.84	24	4.0%	-0.80 [-6.62, 5.02]
Politte 2020 week 8 (SRS)	-0.3	12.25	24	0.4	7.84	24	4.0%	-0.70 [-6.52, 5.12]
Zimmerman 2021 week 7 (SRS)	-0.13	2.47	15	-0.13	1.77	15	56.8%	0.00 [-1.54, 1.54]
**Subtotal (95% CI)**			**63**			**63**	**64.8%**	**-0.09 [-1.53, 1.35]**
Heterogeneity: Chi² = 0.11, df = 2 (P = 0.95); I² = 0%								
Test for overall effect: Z = 0.13 (P = 0.90)								
**4.9.2 ≥10 weeks**								
Politte 2020 week 12 (SRS)	-0.1	12.25	24	0.1	7.84	24	4.0%	-0.20 [-6.02, 5.62]
Zimmerman 2021 week 15 (SRS)	-2.25	2.66	12	-1	2.52	12	31.3%	-1.25 [-3.32, 0.82]
**Subtotal (95% CI)**			**36**			**36**	**35.2%**	**-1.13 [-3.08, 0.82]**
Heterogeneity: Chi² = 0.11, df = 1 (P = 0.74); I² = 0%								
Test for overall effect: Z = 1.14 (P = 0.26)								
**Total (95% CI)**			**99**			**99**	**100.0%**	**-0.46 [-1.62, 0.70]**
Heterogeneity: Chi² = 0.93, df = 4 (P = 0.92); I² = 0%								
Test for overall effect: Z = 0.77 (P = 0.44)								
Test for subgroup differences: Chi² = 0.71, df = 1 (P = 0.40), I² = 0%								

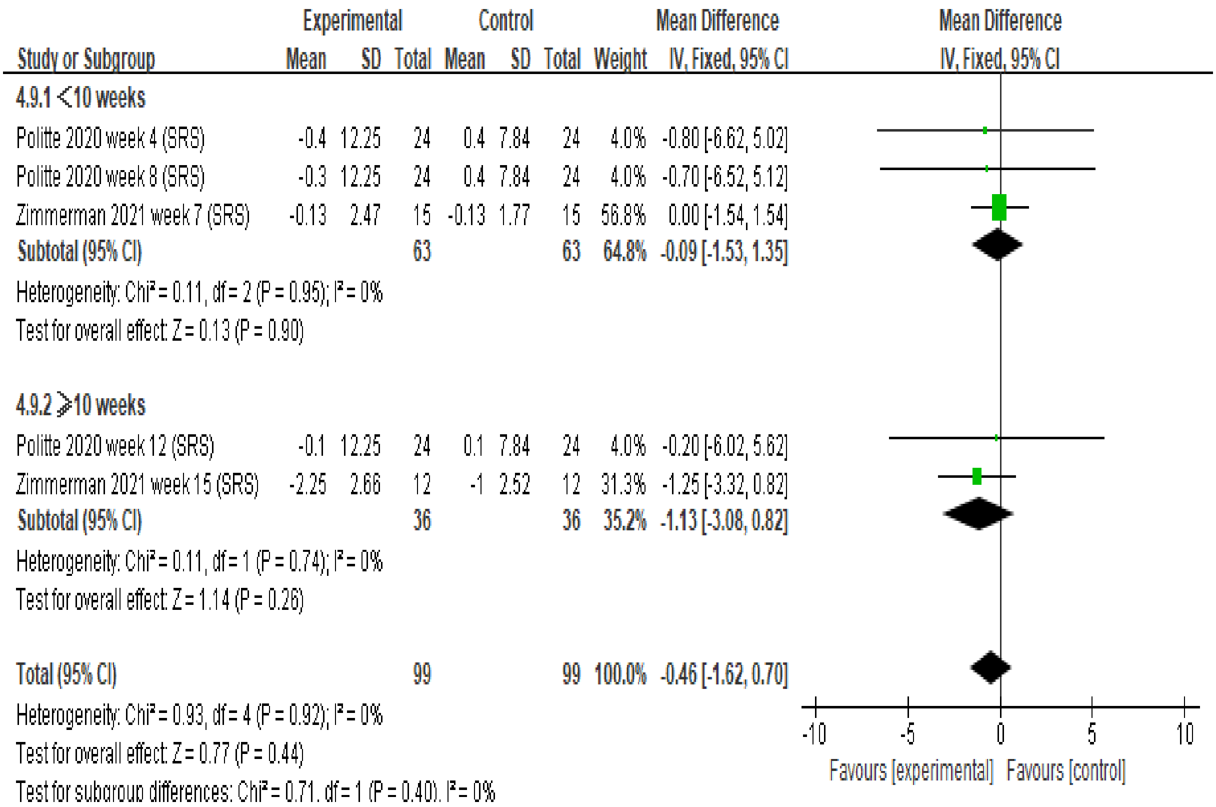

Supplementary Figure 4.5.11: Subgroup analyses of different intervention duration on social awareness

Study or Subgroup	Experimental Mean	Experimental SD	Experimental Total	Control Mean	Control SD	Control Total	Weight	Mean Difference IV, Fixed, 95% CI
**4.10.1 <10 weeks**								
Politte 2020 week 4 (SRS)	-0.8	20.09	24	-0.8	12.74	24	4.9%	0.00 [-9.52, 9.52]
Politte 2020 week 8 (SRS)	-0.9	20.09	24	1	12.74	24	4.9%	-1.90 [-11.42, 7.62]
Zimmerman 2021 week 7 (SRS)	1.2	5.58	15	-2	3.07	15	42.9%	3.20 [-0.02, 6.42]
**Subtotal (95% CI)**			**63**			**63**	**52.7%**	**2.43 [-0.48, 5.33]**
Heterogeneity: Chi² = 1.26, df = 2 (P = 0.53); I² = 0%								
Test for overall effect: Z = 1.64 (P = 0.10)								
**4.10.2 ≥10 weeks**								
Politte 2020 week 12 (SRS)	-0.4	20.09	24	0.5	12.74	24	4.9%	-0.90 [-10.42, 8.62]
Zimmerman 2021 week 15 (SRS)	-2.42	4.5	12	-1.58	3.55	12	42.4%	-0.84 [-4.08, 2.40]
**Subtotal (95% CI)**			**36**			**36**	**47.3%**	**-0.85 [-3.92, 2.22]**
Heterogeneity: Chi² = 0.00, df = 1 (P = 0.99); I² = 0%								
Test for overall effect: Z = 0.54 (P = 0.59)								
**Total (95% CI)**			**99**			**99**	**100.0%**	**0.88 [-1.23, 2.99]**
Heterogeneity: Chi² = 3.57, df = 4 (P = 0.47); I² = 0%								
Test for overall effect: Z = 0.82 (P = 0.41)								
Test for subgroup differences: Chi² = 2.30, df = 1 (P = 0.13), I² = 56.5%								

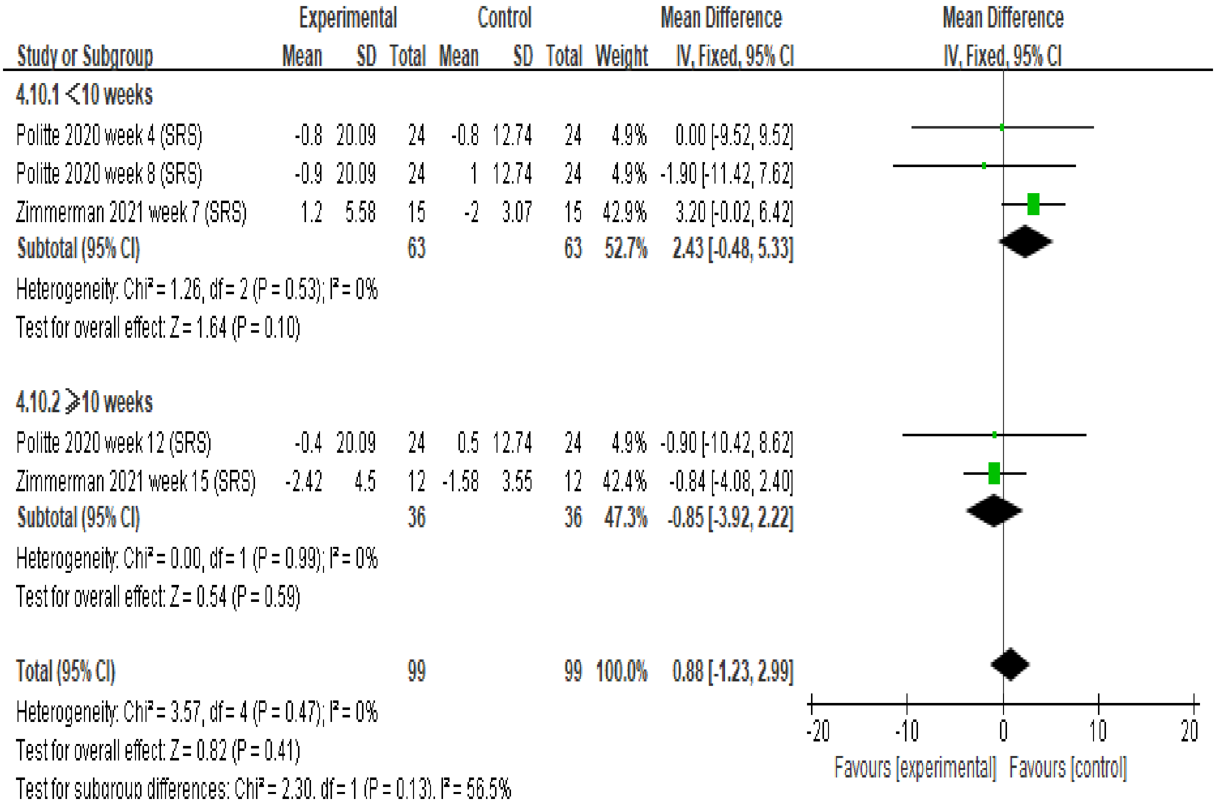

Supplementary Figure 4.5.12: Subgroup analyses of different intervention duration on social cognition

Study or Subgroup	Experimental Mean	SD	Total	Control Mean	SD	Total	Weight	Std. Mean Difference IV, Random, 95% CI
**4.3.1 <10 weeks**								
Momtazmanesh 2020 week 5 (ABC)	-2.57	0.7	30	-1.77	0.43	30	9.0%	-1.36 [-1.92, -0.79]
Politte 2020 week 4 (ABC)	-0.5	24.99	24	0.5	15.68	24	8.9%	-0.05 [-0.61, 0.52]
Politte 2020 week 4 (SRS)	-0.7	20.09	24	0.7	12.25	24	8.9%	-0.08 [-0.65, 0.48]
Politte 2020 week 8 (ABC)	-0.5	24.99	24	0.5	15.68	24	8.9%	-0.05 [-0.61, 0.52]
Politte 2020 week 8 (SRS)	-0.9	20.09	24	0.9	12.25	24	8.9%	-0.11 [-0.67, 0.46]
Zimmerman 2021 week 7 (ABC)	0.3	0.73	15	-0.38	0.89	15	7.2%	0.81 [0.06, 1.56]
Zimmerman 2021 week 7 (SRS)	0.73	3.59	15	-1.47	4.13	15	7.3%	0.55 [-0.18, 1.28]
**Subtotal (95% CI)**			**156**			**156**	**59.2%**	**-0.07 [-0.56, 0.43]**
Heterogeneity: Tau² = 0.35; Chi² = 27.81, df = 6 (P = 0.0001); I² = 78%								
Test for overall effect: Z = 0.26 (P = 0.79)								
**4.3.2 ≥10 weeks**								
Momtazmanesh 2020 week 10 (ABC)	-3.5	0.87	30	-3.4	0.63	30	9.6%	-0.13 [-0.64, 0.38]
Politte 2020 week 12 (ABC)	0.3	24.99	24	-0.2	15.68	24	8.9%	0.02 [-0.54, 0.59]
Politte 2020 week 12 (SRS)	0	20.09	24	0	12.25	24	8.9%	0.00 [-0.57, 0.57]
Zimmerman 2021 week 15 (ABC)	-0.19	0.57	12	-0.33	1.06	12	6.7%	0.16 [-0.64, 0.96]
Zimmerman 2021 week 15 (SRS)	-0.83	3.66	12	-2.5	3.94	12	6.6%	0.42 [-0.39, 1.23]
**Subtotal (95% CI)**			**102**			**102**	**40.8%**	**0.03 [-0.24, 0.31]**
Heterogeneity: Tau² = 0.00; Chi² = 1.40, df = 4 (P = 0.84); I² = 0%								
Test for overall effect: Z = 0.25 (P = 0.80)								
**Total (95% CI)**			**258**			**258**	**100.0%**	**-0.02 [-0.31, 0.27]**
Heterogeneity: Tau² = 0.17; Chi² = 30.11, df = 11 (P = 0.002); I² = 63%								
Test for overall effect: Z = 0.13 (P = 0.89)								
Test for subgroup differences: Chi² = 0.12, df = 1 (P = 0.73), I² = 0%								

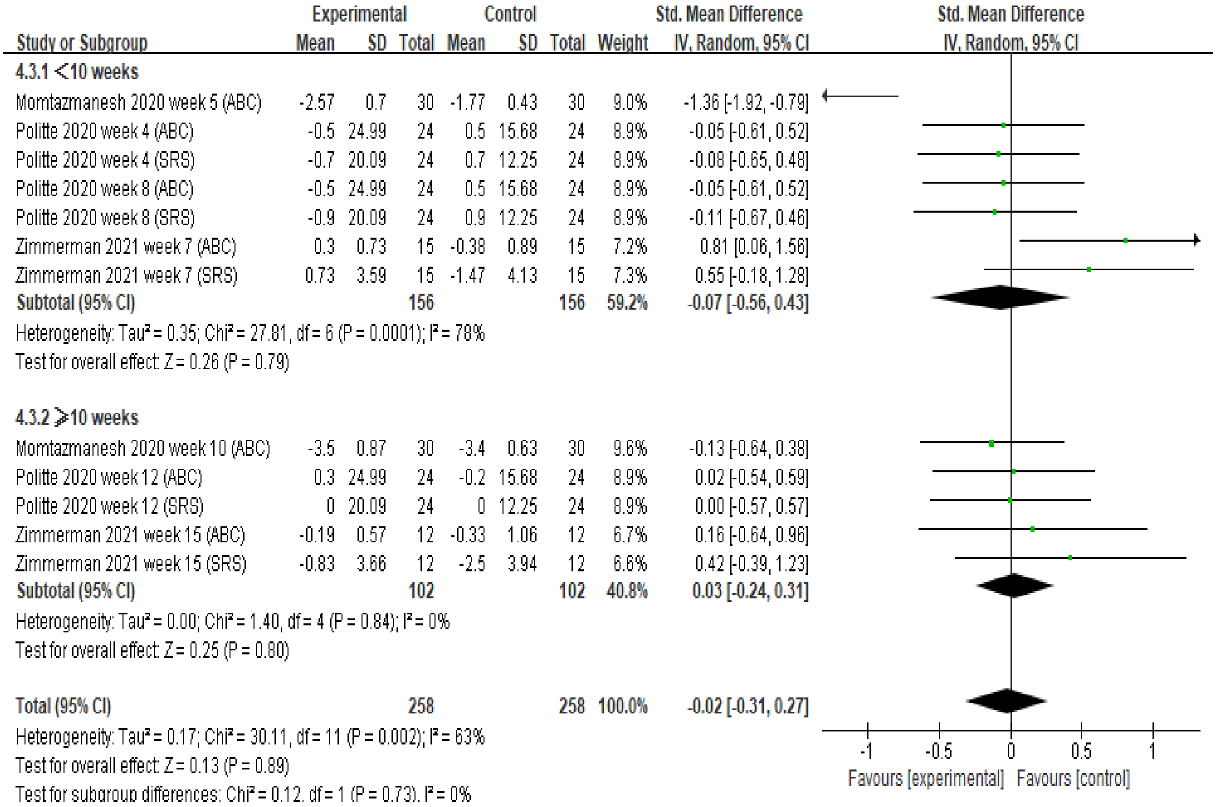

Supplementary Figure 4.5.13: Subgroup analyses of different intervention duration on social motivation
